# Biogenesis and molecular characteristics of serum hepatitis B virus RNA

**DOI:** 10.1371/journal.ppat.1008945

**Published:** 2020-10-20

**Authors:** Sheng Shen, Zhanglian Xie, Dawei Cai, Xiaoyang Yu, Hu Zhang, Elena S. Kim, Bin Zhou, Jinlin Hou, Xiaoyong Zhang, Qi Huang, Jian Sun, Haitao Guo

**Affiliations:** 1 Department of Infectious Diseases, Nanfang Hospital, Southern Medical University, Guangzhou, China; 2 Department of Microbiology and Immunology, Indiana University School of Medicine, Indianapolis, IN, United States of America; 3 Department of Microbiology and Molecular Genetics, University of Pittsburgh School of Medicine, Pittsburgh, PA, United States of America; 4 Assembly Biosciences, Inc., South San Francisco, CA, United States of America; 5 Cancer Virology Program, UPMC Hillman Cancer Center, University of Pittsburgh School of Medicine, Pittsburgh, PA, United States of America; The Research Institute at Nationwide Children's Hospital, UNITED STATES

## Abstract

HBV is an enveloped DNA virus that replicates its DNA genome *via* reverse transcription of a pregenomic (pg) RNA intermediate in hepatocytes. Interestingly, HBV RNA can be detected in virus-like particles in chronic hepatitis B (CHB) patient serum and has been utilized as a biomarker for intrahepatic cccDNA activity in treated patients. However, the biogenesis and molecular characteristics of serum HBV RNA remain to be fully defined. In this study, we found that the encapsidated serum HBV RNA predominately consists of pgRNA, which are detergent- and ribonuclease-resistant. Through blocking HBV DNA replication without affecting pgRNA encapsidation by using the priming-defective HBV mutant Y63D or 3TC treatment, we demonstrated that the cell culture supernatant contains a large amount of pgRNA-containing nonenveloped capsids and a minor population of pgRNA-containing virions. The formation of pgRNA-virion requires both capsid assembly and viral envelope proteins, which can be inhibited by capsid assembly modulators and an envelope–knockout mutant, respectively. Furthermore, the pgRNA-virion utilizes the multivesicular body pathway for egress, in a similar way as DNA-virion morphogenesis. Northern blotting, RT-PCR, and 3’ RACE assays revealed that serum/supernatant HBV pgRNA are mainly spliced and devoid of the 3’-terminal sequences. Furthermore, pgRNA-virion collected from cells treated with a reversible HBV priming inhibitor L-FMAU was unable to establish infection in HepG2-NTCP cells. In summary, serum HBV RNA is secreted in noninfectious virion-like particle as spliced and poly(A)-free pgRNA. Our study will shed light on the molecular biology of serum HBV RNA in HBV life cycle, and aid the development of serum HBV RNA as a novel biomarker for CHB diagnosis and treatment prognosis.

## Author summary

Although increasing evidence supports the presence of extracellular HBV RNA (or serum HBV RNA) species and their potentials to be a new marker for monitoring chronic HBV infection, the origin and molecular forms of serum HBV RNA remain ill-defined. In addition to the infectious DNA virions, HBV is known to produce a number of incomplete viral particles extracellularly, including subviral particles (HBsAg), naked capsids, and empty virions, during its replication cycle. Here, we demonstrated that HBV pregonomic (pg) RNA-containing virion-like-particles exist in cell culture fluid and patient sera, which are secreted along with mature DNA virions *via* the multivesicular body (MVB) secretory route. Moreover, virion-derived HBV RNAs are predominantly spliced and 3’ truncated forms, and HBV RNA virions are unable to initiate a new round of infection in cell cultures. These findings provide new insights into a better understanding of serum HBV RNA biology and a conceptual basis for further widespread clinical applications of serum HBV RNA as a biomarker in chronic hepatitis B patients.

## Introduction

Hepatitis B virus (HBV) causes both acute and chronic infections. Chronic hepatitis B (CHB) remains a major public health problem affecting over 257 million people [1]. HBV is an enveloped DNA virus belonging to the *Hepadnaviridae* family. The virion contains a 3.2-kb partially double-stranded (ds) relaxed circular (rc) DNA genome. Upon infection of a hepatocyte through the hepatocyte-specific receptor NTCP, the viral rcDNA is transported into the nucleus to form an episomal covalently closed circular DNA (cccDNA), which exists in a form of minichromosome. By utilizing the host RNA polymerase II, cccDNA transcribes five mRNAs with overlapping 3’ end, namely the 3.5-kb precore (pc) and pregenomic (pg) RNA, 2.4/2.1-kb surface mRNAs, and 0.7-kb X mRNA. HBV replicates its DNA genome *via* protein-primed reverse transcription of pgRNA, catalyzed by viral polymerase, in the cytoplasmic nucleocapsid. The newly synthesized rcDNA-containing nucleocapsid is enveloped by viral surface proteins and secreted through the cellular multivesicular body (MVB) secretory pathway to yield progeny virion. In addition to the DNA-containing virions, a surplus of nucleocapsid-free subviral particles self-assembled in the endoplasmic reticulum (ER) lumen are secreted through the constitutive secretory pathway, which accumulate extracellularly as HBsAg [2, 3]. Furthermore, a large number of genome-free empty virions and nonenveloped “naked” capsids can also be detected extracellularly [4].

Except for the empty virion, it has been a long-standing dogma that only the mature, dsDNA-containing HBV nucleocapsid can be enveloped for virion secretion, while the immature nucleocapsids possess a putative single strand blocking signal for envelopment [5]. Interestingly, circulating HBV RNA has been detected in CHB patient blood since more than two decades ago, which may represent an exception to the dogma [4, 6]. Recently, one study reported that HBV RNA in patient serum or cell culture fluid is the encapsidated pgRNA present in virion. However, it did not rule out the contamination from naked capsids, which contain all types of HBV replicative intermediates [4, 7]. In line with this, another study showed that the extracellular HBV RNA are predominantly associated with naked capsids in cell cultures, but exist in capsid immunocomplexes and virions in patient sera [8]. Thus, the physical presence of serum HBV RNA remains to be defined.

Currently, nucleot(s)ide analogues (NAs) are prescribed for CHB, which could potently suppress HBV replication to even undetectable level, improve long-term outcome and reduce risks of liver cancer [9]. However, HBV infection cannot be completely eliminated due to the paucity of direct antiviral effect of NAs on cccDNA [10]. Given that NAs have no or little effect on the transcriptional activity of cccDNA, HBV RNAs are continuously produced under NA treatment, even if HBV DNA level becomes undetectable. Thus, HBV RNA, especially the pgRNA, may be a potential new marker for monitoring cccDNA activity in CHB [6]. Recent studies have reported that serum HBV RNA level was associated with responses to pegylated interferon (peg-IFN) or NAs, and may guide the discontinuation of NA-therapy [7, 11–13]. However, the nature of serum HBV RNA remains elusive. It has been reported that extracellular HBV RNA contains pgRNA and spliced RNA variants in the supernatant of HBV-infected cell cultures and in CHB patient serum [14, 15]. Another study reported that the serum HBV RNA are of heterogeneous lengths, ranging from the full-length pgRNA to a few hundred nucleotides with 3’ end truncations [8]. Therefore, the molecular characteristics of serum HBV RNA species needs to be fully defined and a standardized detection method is warranted. Moreover, if the encapsidated pgRNA could be enveloped, it is interesting to know how the HBV RNA virions are released from hepatocytes, and whether these RNA virions are infectious.

Here, we report that HBV pgRNA-containing virion-like-particles (VLPs) exist in culture fluids and patient sera, which are assembled intracellularly through pgRNA encapsidation and envelopment, and secreted *via* the MVB secretory route. Moreover, virion-derived HBV RNAs are predominantly spliced pgRNA devoid of the 3’-terminal sequences, and HBV RNA virions are unable to initiate a new round of infection in cell cultures. Our study thus provides new insights into a better understanding of serum HBV RNA biology and the further clinical applications of serum HBV RNA as a biomarker for persistent HBV infection.

## Results

### Evidence of HBV RNA-VLPs in cell cultures

**Fig 1:**
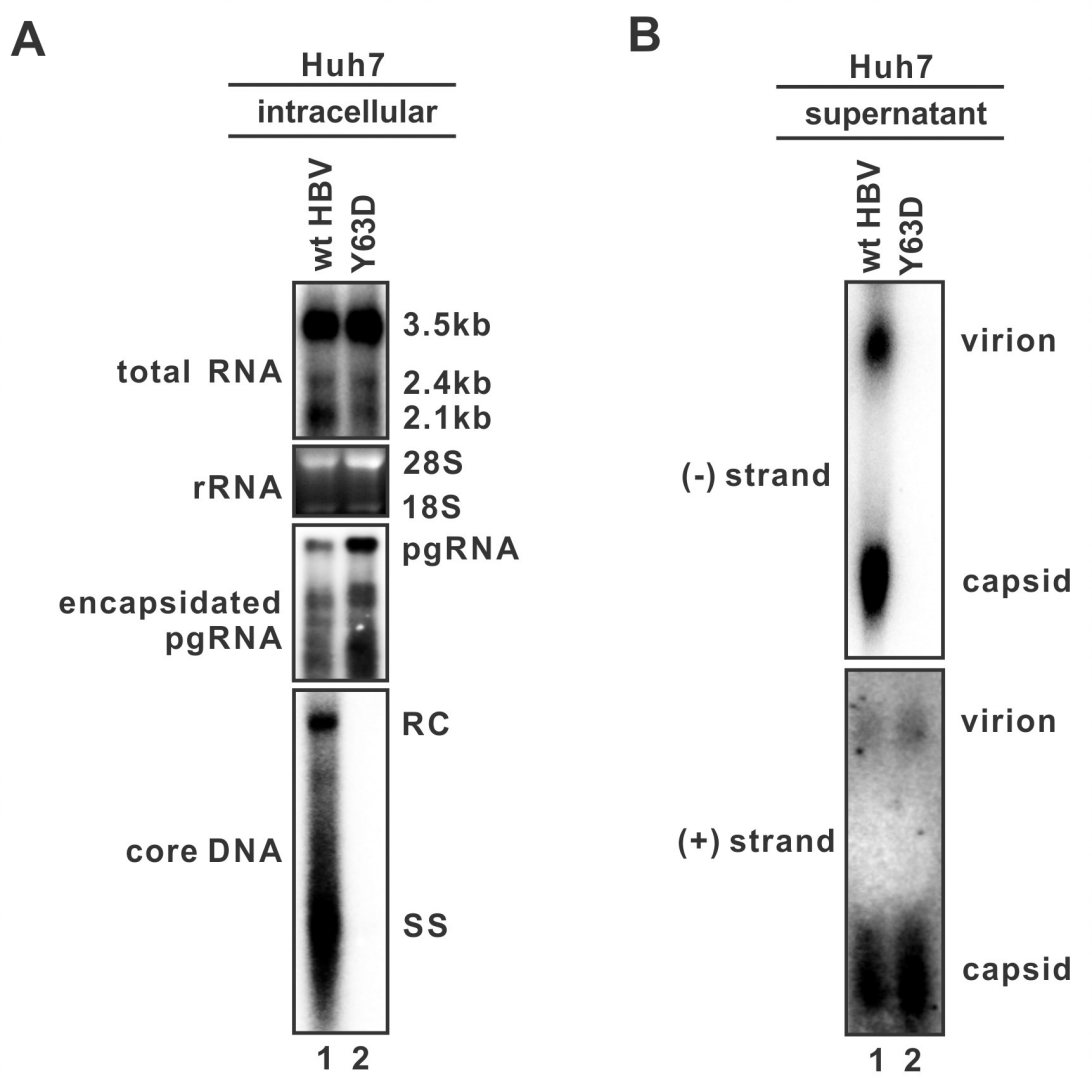
The presence of HBV RNA virion-like-particles (VLPs) in cell culture fluid. (A) Huh7 cells in 6-well plate were transfected with 3.2 μg of pCMVHBV (wt HBV) or pCMVHBV-Y63D (Y63D mutant). Cells were harvested at day 5 post-transfection and the intracellular viral total RNA, encapsidated pgRNA, and core DNA were analyzed by Northern bot (upper panels) and Southern blot (lower panel), respectively. Ribosomal RNAs (28S and 18S) served as total RNA loading control, and HBV 3.5 kb, 2.4 kb, and 2.1 kb RNAs were hybridized with a plus (+) strand-specific full-length HBV riboprobe. HBV core DNA was probed with a minus (-) strand-specific full-length HBV riboprobe. The positions of relaxed circular (RC), single-stranded (SS) DNAs are indicated. (B) HBV particles in culture fluids of transfected cells at day 5 post-transfection were analyzed by a particle gel assay. HBV virion DNA and nonenveloped naked capsid DNA were detected by (-) strand-specific riboprobe (upper panel). HBV RNA in virion and capsid were detected by (+) strand-specific riboprobe (lower panel). The positions of HBV virion and naked capsid are indicated.

Previous studies have suggested that serum HBV RNA is virion-associated based on evidence from immunoprecipitation and RT-PCR, however, the existence of HBV RNA virion has not been rigorously validated by the conventional particle gel assay [6, 7]. To this end, we determined the RNA virion secretion capacity of a HBV mutant pCMVHBV-Y63D, which bears a Y63D substitution in the terminal protein domain of HBV polymerase to block DNA priming and reverse transcription but still allows pgRNA encapsidation [5]. As shown in Fig 1A, transfection of wildtype (wt) HBV or Y63D mutant into Huh7 cells resulted in viral RNA transcription and pgRNA encapsidation, however, only the wt HBV, but not the Y63D mutant, synthesized viral core DNA. Native particle gel assay demonstrated that no DNA-containing virion or naked capsid was produced in the supernatant by the Y63D mutant (Fig 1B, top panel). However, after hybridizing with (+) strand-specific HBV probe, both RNA-containing VLPs and naked capsids were detected in the supernatant of Y63D-transfected cells, with signal intensity being slightly higher than that from wt HBV (Fig 1B, bottom panel). The results indicated that HBV RNA can be released in particles as VLPs and naked capsids prior to viral DNA synthesis, which can be highlighted when viral reverse transcription is completely blocked.

The above notion was further supported by inhibiting HBV DNA replication with reverse transcriptase inhibitor 3TC. As shown in S1 Fig, upon HBV induction in HepAD38 cells, 3TC treatment completely inhibited viral core DNA synthesis and DNA-containing particle production, resulting in an accumulation of intracellular encapsidated pgRNA. Interestingly, the time-dependent release of HBV RNA-containing VLPs and capsids from 3TC-treated HepAD38 cells exhibited a similar kinetics with the viral particle production from untreated cells. The results further demonstrate that the supernatant HBV RNA are associated with VLPs and naked capsids, and the levels of such viral RNA particles can be modestly enhanced through blocking intracellular viral DNA replication by a reverse transcription inhibitor.

### The production of HBV RNA-VLP requires pgRNA encapsidation

**Fig 2:**
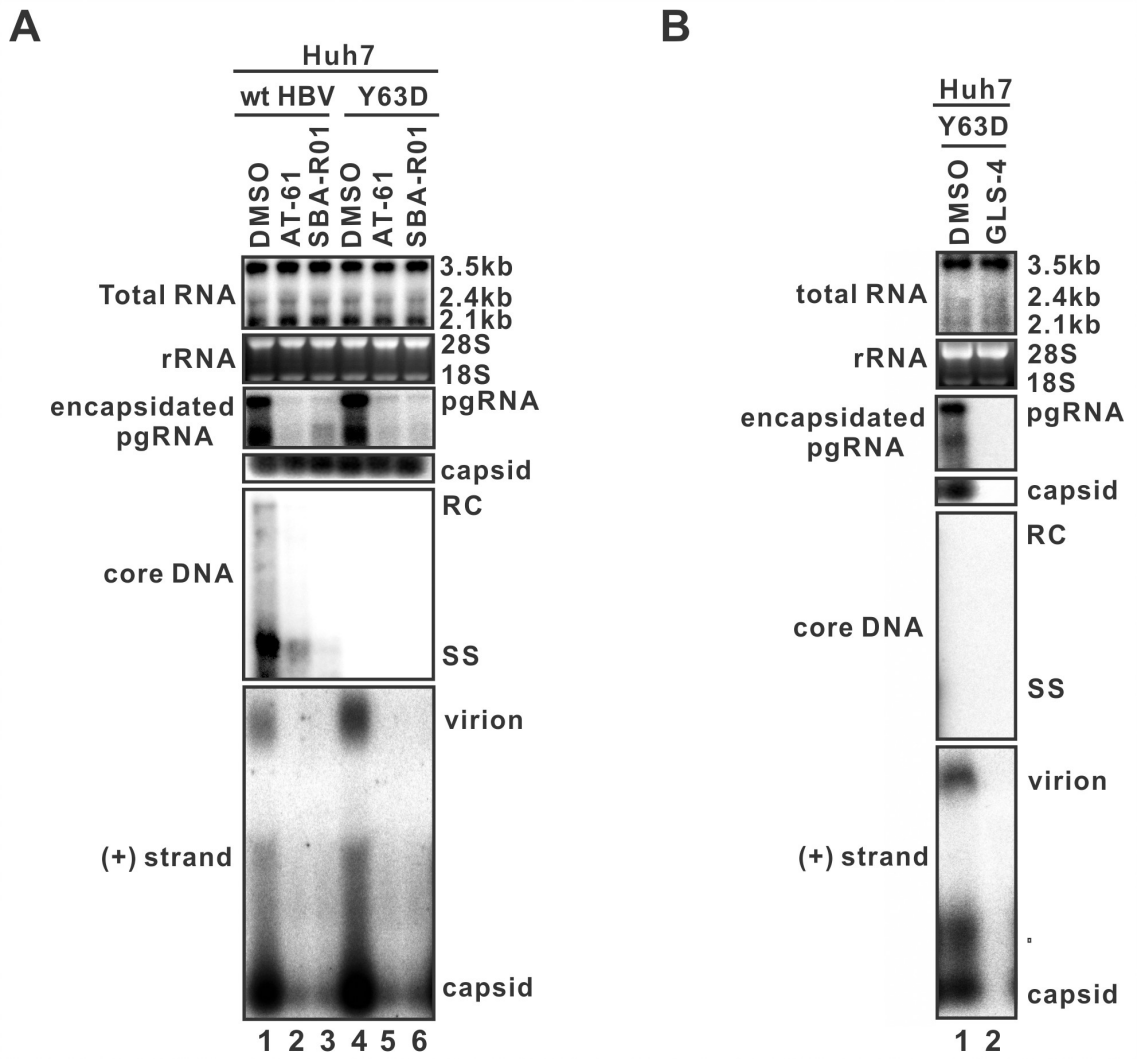
The production of HBV RNA-VLP requires pgRNA encapsidation. Huh7 cells in 6-well plate were transfected with pCMVHBV (wt HBV) or pCMVHBV-Y63D (Y63D) and treated with solvent control (DMSO), AT-61, SBA-R01 (A) or GLS-4 (B). 5 days later, the intracellular total viral RNA, encapsidated pgRNA, capsid and core DNA were analyzed by Northern blot, capsid enzyme immunoassay (EIA) and Southern blot assays. HBV particles in culture fluids were partitioned in native particle gel and the encapsulated viral RNA were detected by (+) stand-specific HBV probe.

Given the fact that the HBV RNA-VLPs migrated at the DNA-virion position in particle gel, we hypothesized that RNA-VLP contains HBV nucleocapsid. To test the hypothesis, the virion secretion capacity of wt HBV and Y63D mutant was assessed in cells treated with capsid assembly modulator (CpAM) AT-61, SBA-R01 or GLS4. While GLS-4 is a Class-I CpAM that induces non-capsid HBV core polymer for degradation, AT-61 and SBA-R01 belong to the Class-II CpAMs that allow HBV capsid formation devoid of pgRNA [16]. As shown in Fig 2A, AT-61 and SBA-R01 treatment did not affect HBV RNA transcription or capsid formation but significantly inhibited pgRNA encapsidation in both wt HBV- and Y63D mutant-transfected cells. Consequently, the amounts of intracellular HBV core DNA, extracellular virions and naked capsids were remarkably reduced in wt HBV-transfected cells. More importantly, the Class-II CpAMs dramatically inhibited the production of HBV RNA-containing naked capsids and VLPs from Y63D mutant-transfected cells. In addition, the Class-I CpAM GLS4 also abolished the release of HBV RNA-VLP and RNA-capsid from Y63D-transfected cells through blocking capsid assembly (Fig 2B). The above results thus imply that, like HBV DNA-virions, pgRNA encapsidation is a premise for the production of HBV RNA-VLPs.

### The production of HBV RNA-VLP requires envelopment

**Fig 3:**
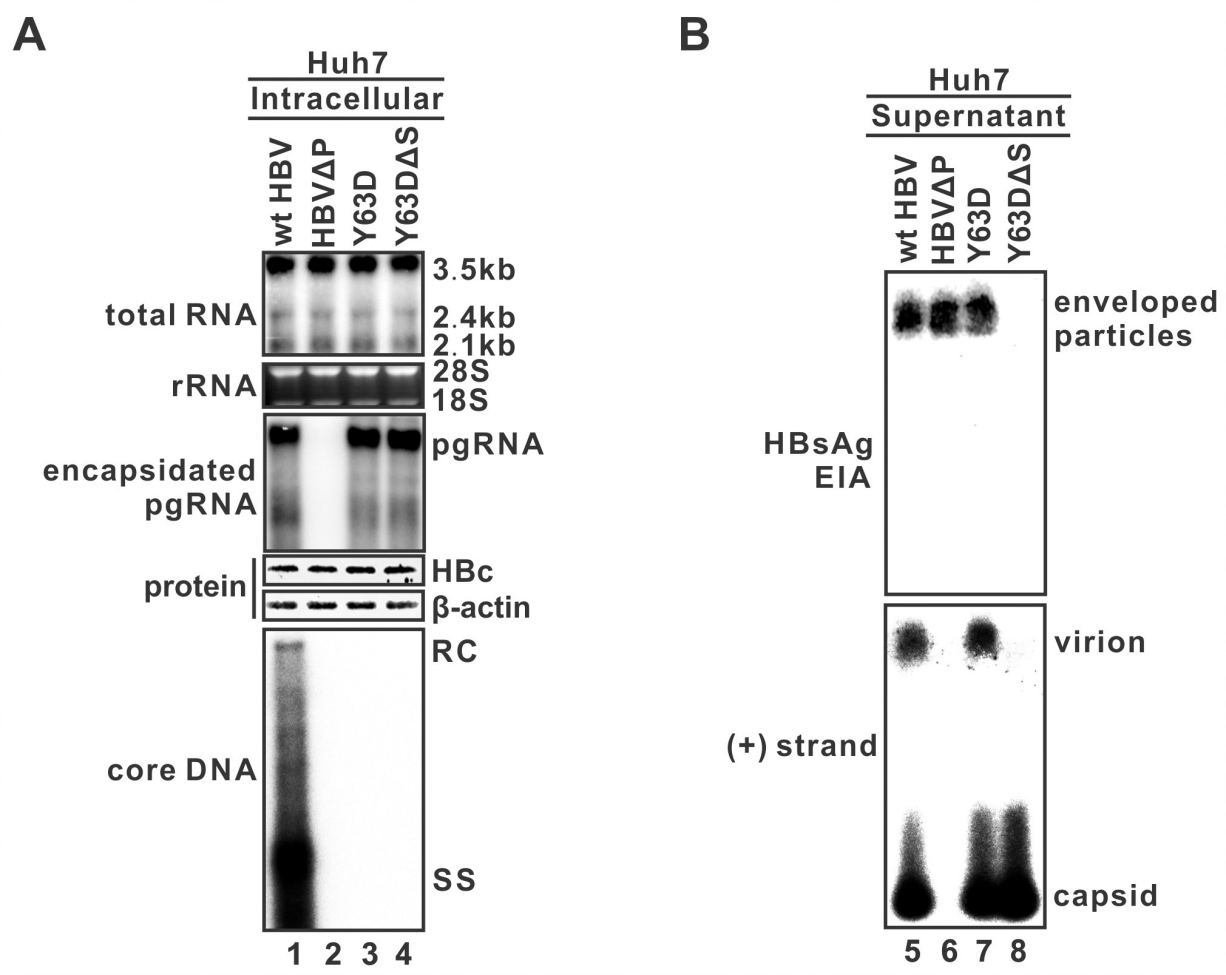
The production of HBV RNA-VLP requires envelopment. Huh7 cells in 6-well plate were transfected with pCMVHBV (wt HBV), Pol-null mutant pCMVHBVΔPol (HBVΔP), pCMVHBV-Y63D (Y63D), and envelope-null mutant pCMVHBV-Y63DΔS (Y63DΔS), respectively, for 5 day. (A) The intracellular total viral RNA, encapsidated pgRNA, core protein, and core DNA were analyzed by Northern, Western and Southern blot assay, respectively. Ribosomal RNA and β-actin served as loading controls for total RNA and protein, respectively. (B) HBV particles in culture fluids were analyzed by particle gel assay. Enveloped HBV particles, including virions and subviral particles (HBsAg), were revealed by EIA using HBsAb (upper panel). HBV RNA in virions and naked capsids were detected by (+) strand-specific probe (lower panel).

To further test whether the secretion of RNA-VLP needs envelopment, we determined the virion secretion capacity of four HBV constructs, specifically pCMVHBV, pCMVHBVΔPol, pCMVHBV-Y63D and pCMVHBV-Y63DΔS, of which the ΔPol mutant is defective in polymerase expression and unable to form pgRNA-containing nucleocapsid, whereas ΔS mutant prevents the expression of three HBV envelope proteins. As shown in Fig 3A, transfection of the above individual plasmids in Huh7 cells exhibited comparable HBV RNA transcription and core protein expression, pgRNA was not found in intracellular capsids from ΔPol mutant, and no viral DNA was detected in capsids from the ΔPol, Y63D and Y63DΔS mutants. Furthermore, enveloped HBV particles were detected in the supernatant of cells transfected with wt HBV, Y63D or ΔPol mutant, but not the Y63DΔS mutant, by particle gel HBsAg EIA (Fig 3B). When probed with (+) strand-specific HBV riboprobe, consistent with the results in Fig 2, hybridization signals were detected in naked capsids and VLPs derived from the wt HBV and Y63D mutant, but not the ΔPol mutant (Fig 3B. bottom panel). Furthermore, abolishing viral envelope protein expression eliminated RNA signal at the VLP position from Y63DΔS mutant without affecting the RNA-containing capsids, indicating that the secretion of the HBV RNA-VLPs, similar to DNA-containing virions, is dependent upon the viral envelope proteins. Hence, we conclude that the HBV RNA-VLP is a new type of HBV virion with enveloped pgRNA-containing nucleocapsid.

### HBV RNA-virion utilizes MVB pathway for egress

**Fig 4:**
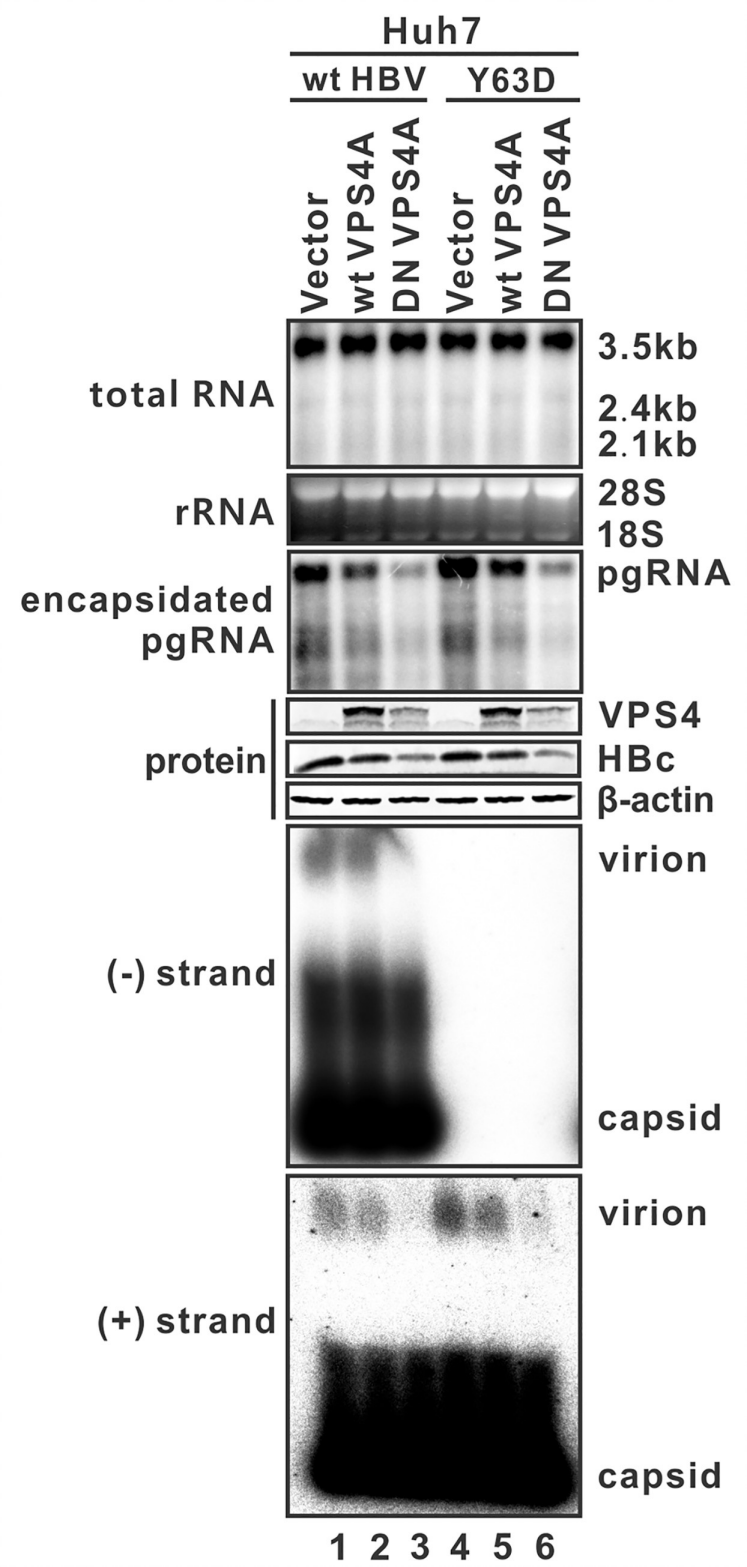
HBV RNA-virion utilizes MVB pathway for egress. Huh7 cells in 6-well plate were cotransfected with the pCMVHBV (wt HBV) or pCMVHBV-Y63D (Y63D) and control vector, or GFP/DsRed-tagged wildtype (wt) VPS4A or dominant-negative (DN) version of VPS4A at a 9:1 DNA weight ratio (2.7:0.3 μg). Cells were harvested at day 5 post-transfection and the intracellular total viral RNA, encapsidated pgRNA, HBc and VPS4A proteins were analyzed by Northern and Western blot, respectively. HBV particles in culture fluids were analyzed by particle gel assay. HBV DNA/RNA in virions and naked capsids were detected by (-) strand- and (+) strand-specific probes.

It is known that HBV DNA-virion usurps the cellular ESCRT-dependent MVB secretory pathway for egress [17]. Next, we set out to test whether the secretion of HBV RNA-virions shares the MVB secretory route with DNA-virions. To impair the MVB pathway, we employed an ATP hydrolysis-deficient, dominant-negative (DN) mutant of VPS4A, to examine its effect on the release of HBV RNA-virions. VPS4A is an ATPase required to dissociate and recycle all three ESCRT complexes from the endosomal membrane for further use, the DN VPS4A mutant locks the ESCRT machinery on the endosomal surface, blocks general endosomal protein and lipid transport and recycling, deranges endosomal membrane morphology [18]. Huh7 cells were cotransfected with wt HBV or Y63D mutant and the wt or DN VPS4A. As shown in Fig 4, the expression of wt or DN VPS4A had no obvious effect on HBV RNA transcription or the release of naked capsids. The slight reduction of extracted intracellular encapsidated pgRNA and core protein by DN VPS4A was due to that the inhibition of VPS4A entrapped the viral core particles in detergent-insoluble membrane structures [19]. However, consistent with previous studies [19, 20], expression of DN VPS4A reduced the secretion of both HBV DNA- and RNA-virions to nearly undetectable level compared to control and wt VPS4A, but did not apparently affect the levels of RNA-containing naked capsids. Collectively, these results indicate that, similar to HBV DNA-virion, the egress of RNA-virion requires the MVB secretory machinery.

### Extracellular HBV pgRNA are shorter species with heterogeneous length

**Fig 5:**
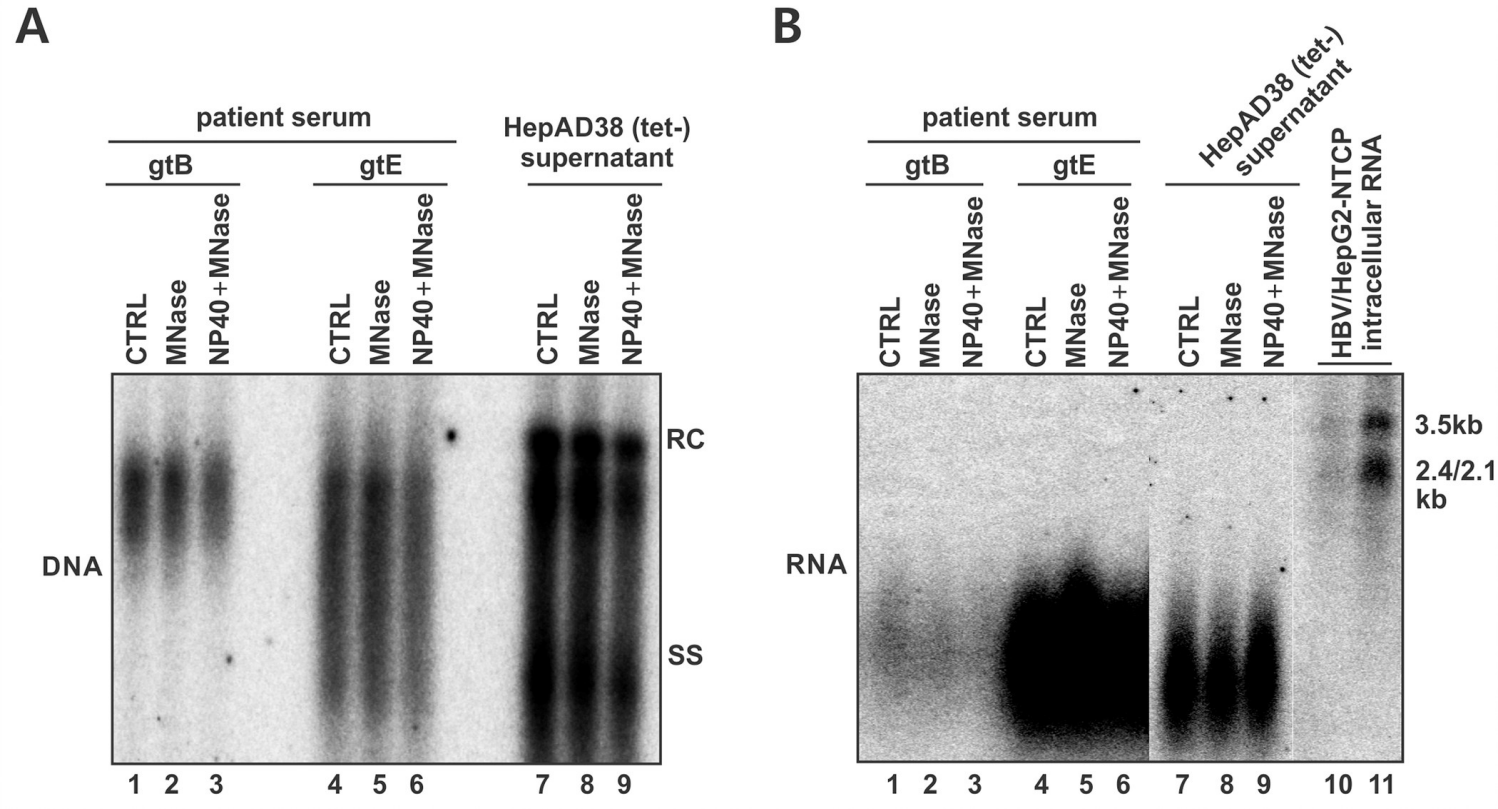
Supernatant/serum HBV RNA are short heterogeneous RNA species. Naïve HBV genotype (gt) B and E patient sera and the supernatant of induced HepAD38 (tet-) were either untreated or treated with NP40 (final concentration of 0.5%) for 10 min at room temperature, followed by micrococcal nuclease (MNase, 20 units/μl) digestion in the presence of 5mM CaCl2 for 15 min at 37°C or remain untreated. Viral DNA/RNA were co-purified by QIAamp MinElute Virus Spin Kit and analyzed by Southern blotting (A). After DNase I digestion, the samples were subjected to Northern blot analysis (B). 100 ng and 500 ng total RNA from gtD HBV infected HepG2-NTCP cells (MOI of 100 viral genome equivalent (vge), 4 days post-infection) served as positive control of intracellular HBV RNA. Southern blot and Northern blot were hybridized with (-) strand- and (+) strand-specific HBV probe, respectively.

The requirement of intracellular HBV pgRNA encapsidation for RNA-virion formation indicates that HBV RNA-virion contains pgRNA (Fig 2). To ascertain the profile of extracellular HBV RNA, we examined the viral nucleic acids from CHB patient sera and culture supernatant of HepAD38 cells by conventional Southern and Northern blotting. As shown in Fig 5, the serum HBV DNA from genotype B and E patients exhibited a smeared band pattern as partial double-stranded DNA, whereas the supernatant HBV DNA from HepAD38 cells has an additional single-stranded DNA band, which was from the naked capsids [5, 21]. Pretreatment of serum/supernatant with MNase in the absence or presence of detergent NP40 did not markedly reduce the viral DNA signals, confirming that the DNA are encapsulated in viral particles. Interestingly, comparing to the intracellular HBV RNA from HBV-infected HepG2-NTCP cells, the serum/supernatant HBV RNA were detected as shorter species with heterogeneous lengths on Northern blot (Fig 5B). Consistent with serum/supernatant HBV DNA, the resistance of serum/supernatant HBV RNA to detergent and nuclease pretreatment indicated their existence in viral particles. The RNA nature of these low molecule weight nucleic acids was further confirmed by RNaseA digestion (S2 Fig).

### HBV RNA-virions mainly contain spliced pgRNA

**Fig 6:**
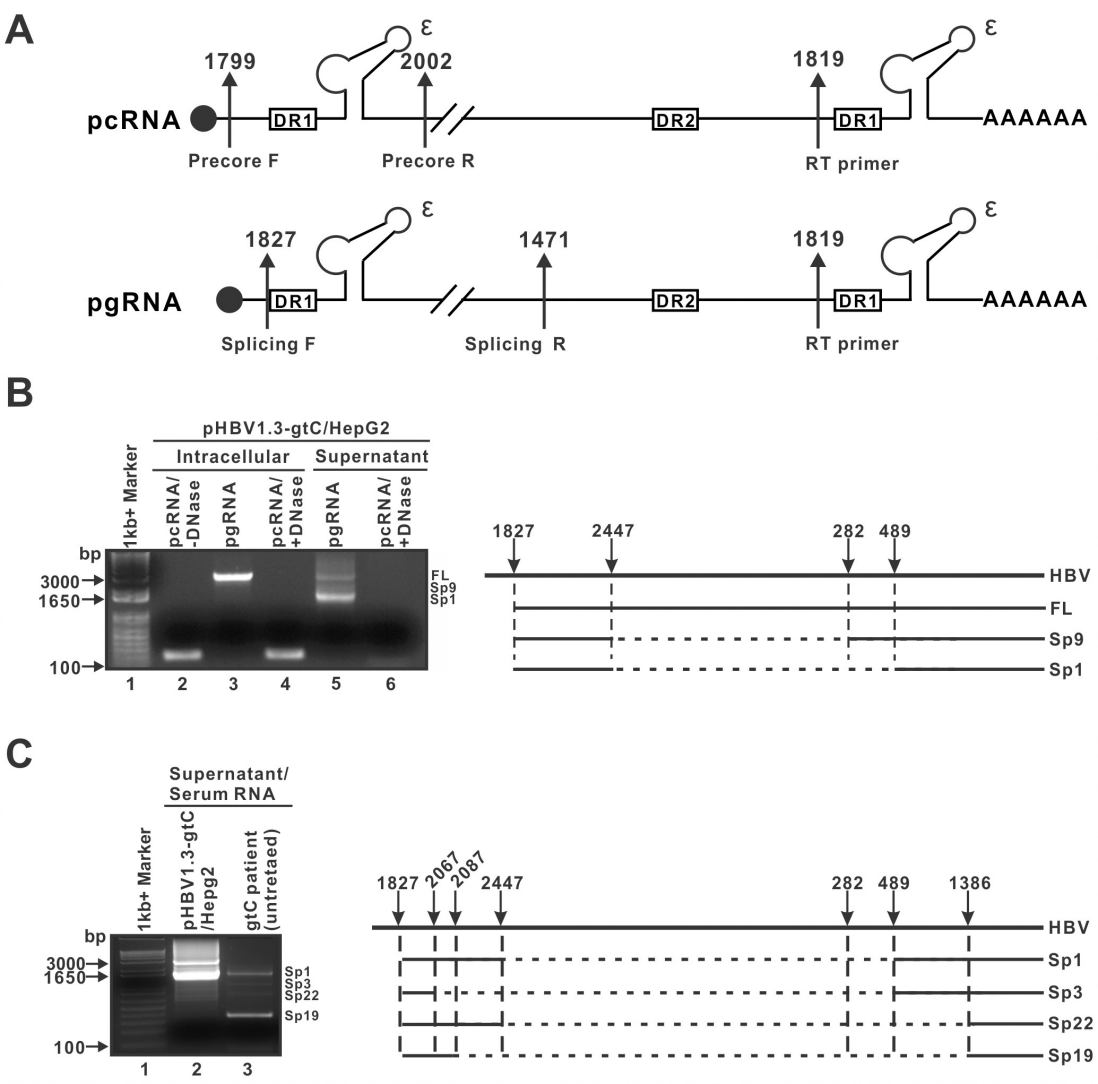
Supernatant/serum HBV RNA are mainly spliced pgRNA. (A) Schematic illustration of the precore mRNA (pcRNA) and pgRNA. The 5’ cap and 3’ poly(A) tail are denoted with a solid dot and a string of adenosines, respectively. The positions of epsilon (ε), direct repeat (DR) 1 and DR2, primers for RT-PCR are indicated. The gap indicates an omitted sequence fragment of intron. (B) HBV RNA from the supernatant of pHBV1.3-gtC transfected cells were amplified by primer sets specific for detecting pcRNA and spliced pgRNA. Intracellular HBV RNA was analyzed alongside extracellular RNA to compare the size and migration patterns of the amplicons (left panel). The intense amplicon bands lower than full-length (FL) amplicon from unspliced pgRNA were sequenced and aligned schematically (right panel). The locations of introns (dashed lines) and exon (solid lines) were indicated. The spliced variant (Sp) numbering was according to S3A Fig. (C) HBV RNA from genotype C CHB patient serum were amplified by RT-PCR using pgRNA splicing-specific primers. Extracellular HBV RNA from supernatant of pHBV1.3-gtC transfected cells were analyzed in the same way for comparison. The distinct migrating bands from patient serum RNA sample were sequenced and the corresponding spliced variants were determined.

HBV pgRNA undergoes posttranscriptional mRNA splicing event at the 5’ portion to generate a series of spliced pgRNA variants [14, 22, 23](S3A Fig). Previous studies reported that the spliced pgRNAs also represent a species of extracellular HBV RNAs [14, 15, 24]. To assess whether the observed heterogeneous length of extracellular HBV RNA was due to RNA splicing (Fig 5), the supernatant RNA from pHBV1.3-gtC-transfected HepG2 cells were reversed transcribed into cDNA by an HBV-specific RT primer, followed by PCR amplification with primers specifically targeting pcRNA, unspliced and spliced pgRNA, respectively (Fig 6A). The intracellular HBV RNA was analyzed alongside extracellular RNA to compare the size and migration patterns of the amplicons. As shown in Fig 6B, the cDNA templates from intracellular HBV RNA generated full-length pcRNA- and pgRNA-specific amplicons. The same intensity of pcRNA RT-PCR amplicon obtained with or without DNase I pretreatment of RNA samples ruled out a potential contamination of RNA samples by HBV core DNA. Moreover, the absence of pcRNA amplicon in supernatant RNA RT-PCR further confirmed that the unencapsidated HBV RNA is incompetent for secretion. Interestingly, besides the full-length pgRNA-specific amplicons, two shorter than full-length amplicons emerged from the supernatant RNA RT-PCR. Sequence analysis showed that these pgRNA variants contained spliced introns as previously reported (S3A Fig), including the spliced variant 1 (Sp1) and Sp9 (Fig 6B). Furthermore, pgRNA splicing variants with different patterns were found in the supernatant of genotype D HBV transiently and stably transfected HepG2 cells, and 3TC treatment did not significantly alter the pattern or ratio of extracellular spliced pgRNA in HepAD38 cells (S3B and S3C Fig).

In order to determine whether the extracellular spliced pgRNA also exists *in vivo*, HBV RNA from genotype C CHB patient serum was assessed. As shown in Fig 6C, the RT-PCR amplicons from patient sample demonstrated that the serum HBV RNA were predominantly spliced pgRNA, but exhibited a different splicing pattern compared to that from HBV-transfected cells. Specifically, the serum HBV RNA comprised of four distinct migrating bands corresponding to Sp1, Sp3, Sp19 and Sp22, respectively. Sp22 is a novel putative splicing variant that has not been reported before.

### HBV virion-pgRNA are devoid of poly(A) tail

**Fig 7:**
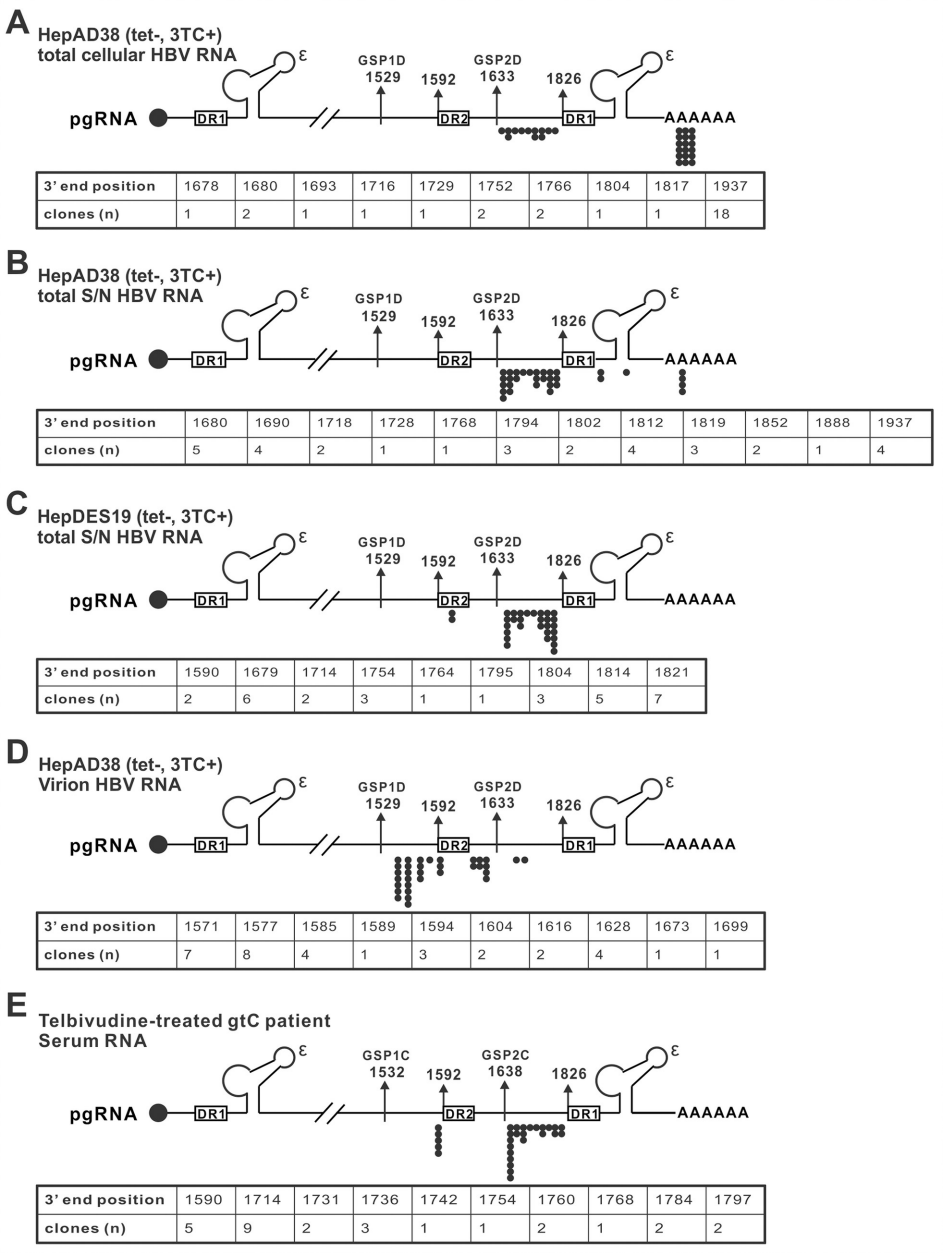
Supernatant/serum HBV RNA are predominately 3’ terminally truncated. HepAD38 and HepDES19 cells were cultured in the absence of tet and treated with 3TC for 18 days. The intracellular and extracellular total HBV RNA from HepAD38 cells (A-B), extracellular HBV capsid RNA from HepDES19 cells (C), and purified virion RNA from HepAD38 cells (D) were analyzed by 3’ RACE and clone sequencing as described in Materials and Methods. The nucleotide positions and numbers of mapped 3’ termini of each HBV RNA samples are indicated with solid dots underneath the illustrated full-length pgRNA and listed in tables. The RNA molecules with mapped 3’ end position at nt1937 are polyadenylated. The positions of DR1, DR2, and gtD HBV gene-specific primers for nested PCR (GSPD1 and GSPD2) are indicated. (E) The serum RNA of a telbivudine-treated gtC patient were analyzed by 3’ RACE using gtC-specific primers (GSPC1 and GSPC2) in the similar way as above described.

The lengths of intracellular spliced pgRNA species vary from 1kb to 2.5kb [22]. However, the serum/supernatant pgRNA are even shorter than the 2.4/2.1kb HBV subgenomic RNA as shown on the Northern blot (Fig 5B), indicating that the HBV virion-pgRNA may be further fragmented at 3’ end. Thus, we set out to map the exact 3’ termini of the supernatant pgRNA species by 3’ RACE and sequencing (approximately 30 clones were sequenced for each RACE assay). The induced HepAD38 cells under 3TC treatment were used to enrich the RNA-virion production (S1 Fig). Firstly, the 3’ termini of half of the total intracellular HBV RNA were mapped to the poly(A) tail of the pgRNA sequence, the other half were mapped to the pgRNA sequence from nt 1652 to 1825 between DR2 and DR1 (Fig 7A). Next, the 3’ termini of total supernatant HBV RNA were predominately mapped to a region between DR2 and DR1 as the intracellular RNA (26 out of 33), with few being scattered downstream of DR1 or in the poly(A) tail (Fig 7B), indicating that the supernatant HBV RNA are mainly 3’ terminally truncated forms originated intracellularly. Considering that the total supernatant HBV RNA exist in both virions (minor) and naked capsids (major) (Figs 1–3 and S1 Fig), we further analyzed the supernatant HBV RNA from induced HepDES19 cells under 3TC treatment, which contain exclusively naked capsid-associated HBV RNA (S4 Fig). Consistently, the 3’ termini of the majority of the naked capsid RNA (28 out of 30) were mapped to the pgRNA sequence between nt 1652 to 1825 (Fig 7C).

Next, RNA-virions from the supernatant of induced, 3TC-treated HepAD38 cells were purified by sucrose gradient centrifugation (S5 Fig), and virion RNA were analyzed by 3’ RACE. As summarized in Fig 7D, the mapped 3’ termini of the virion RNA were mainly clustered in a region surrounding DR2, indicating that the virion RNA possess further recessed 3’ termini compared to naked capsid RNA in cell cultures.

To determine the 3’ termini of serum pgRNA, naïve genotype C HBV patient serum RNA were subjected to RT-PCR as designed in S6A Fig. Unlike the amplicons from HBV-specific RT primer reverse-transcribed samples, no specific amplicons were obtained when using oligo(dT) as RT primer (S6B Fig), indicating that the poly(A) tail might be missing from serum HBV RNA. Furthermore, the 3’ termini of serum HBV RNA from a telbivudine-treated genotype C CHB patient were determined by 3’ RACE. Among the termini of 28 cloned sequences, 23 are spanning between DR2 and DR1, and the remaining are all located at an upstream position (nt1590) adjacent to DR2 (Fig 7E).

Collectively, the virion HBV pgRNA predominately carries a recessed 3’ end upstream of DR1, which is the reverse transcription initiation site of HBV DNA. We reasoned that the 3’-terminal deletion of pgRNA is catalyzed by the RNaseH activity of HBV polymerase after the minus strand DNA elongation being initiated at DR1. To test this hypothesis, we made use of the Y63D HBV mutant, which is defective in DNA priming activity and thus the RNaseH activity is inactivated due to the absence of viral DNA/RNA duplex formation. The 3’ RACE analysis of the supernatant RNA from Y63D-transfected cells revealed that the 3’ end of extracellular HBV RNA sequence is located between 3’ DR1 and poly(A) tail (S7 Fig). In contrast to the wt HBV (Fig 7), none of the 3’ ends of viral RNA was located upstream of 3’ DR1 under Y63D condition (S7 Fig), suggesting a role of viral RNaseH in trimming the 3’ end of HBV pgRNA at the upstream positions adjacent to 3’ DR1 during NA-arrested reverse transcription.

Interestingly, the supernatant Y63D HBV RNA possess 3’ truncations up to the 3’ end of DR1 (S7 Fig), which is reminiscent of a previous study showing that the encapsidated duck hepatitis B virus (DHBV) pgRNA has an underrepresented 3’ end region downstream of the 3’ DR1, regardless of MNase being used in the capsid RNA extraction or not [25]. We have also observed that the longest HBV capsid pgRNA species is slightly shorter than the 3.5kb HBV RNA on Northern blot (S8 Fig). Collectively, the results indicate that, similar to DHBV, the 3’ end of HBV pgRNA sequence downstream of DR1 is not packed into the interior of viral capsid or not well protected by the capsid from cellular ribonuclease digestion.

### HBV RNA-virion is unable to establish infection *in vitro*

Considering the presence of RNA-virion in HBV replication cycle, it is interesting to assess whether the RNA-virion is able to initiate a new round of infection. In addition to the *bona fide* infectious DNA-virion, a number of extracellular HBV nucleic acids-containing viral particles have been identified, including the DNA- and pgRNA-containing capsids, and RNA-virions. Due to the technical impracticality of separating RNA-virions from DNA-virions, we first examined the infectivity of total viral particles and 3TC-arrested RNA-containing viral particles collected from HepAD38 cells, and the total naked capsids and 3TC-arrested RNA-containing naked capsids collected from HepDES19 cells, in HepG2-NTCP cells. As shown in S9 Fig, while the DNA-virion successfully infected HepG2-NTCP cells as evidenced by HBc immunofluorescence and HBeAg ELISA, the naked capsids, without viral envelope for NTCP binding, failed to establish infection; Consistent with a previous report [26], the 3TC-arrested RNA-virion was noninfectious due to the irreversible DNA chain termination activity of the incorporated nucleotide analogue.

**Fig 8:**
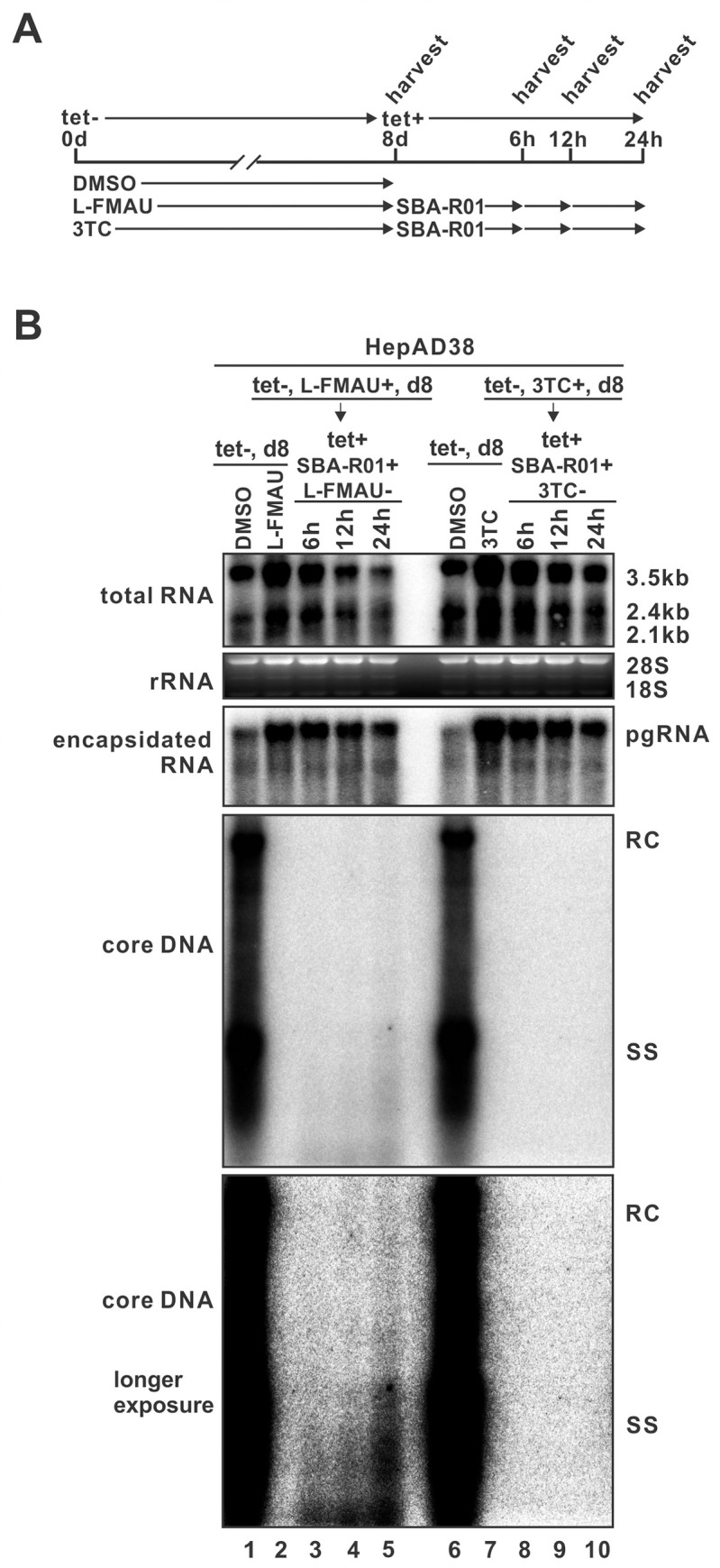
The reversible inhibition of HBV DNA replication by L-FMAU. As depicted in panel (A), HepAD38 cells were treated with solvent DMSO or L-FMAU (40 μM) or 3TC (40 μM) for 8 days after removal of tet from medium. On day 8, L-FMAU or 3TC treatment was ceased and switched to SBA-R01 (10 μM) treatment in the presence of tet. The cells were harvested at the indicated time points. (B) The intracellular total RNA, encapsidated pgRNA and core DNA were analyzed by Northern and Southern blot hybridization, respectively.

In order to obtain replication-competent HBV RNA-virion, we utilized another nucleoside RT inhibitor (NRTI) clevudine (L-FMAU). Unlike other NRTIs, L-FMAU non-competitively inhibits HBV DNA priming and elongation without being incorporated into viral DNA [27]. To assess a potential reversible inhibition of HBV replication by L-FMAU, HepAD38 cells were induced and treated with L-FMAU or 3TC to accumulate cytoplasmic pgRNA-containing capsids, followed by NRTI withdrawal and SBA-R01 treatment in the presence of tet to block *de novo* pgRNA encapsidation (Fig 8A). As shown in Fig 8B, HBV ssDNA replication in L-FMAU-arrested nucleocapsids was resumed rapidly after 6 hours, and partially double-stranded DNA was synthesized within 24 hours. In contrast, 3TC-arrested nucleocapsids failed to resume HBV DNA replication due to 3TC-mediated irreversible chain termination. Furthermore, the cytoplasmic nucleocapsids from untreated, 3TC- or L-FMAU-treated HepAD38 cells were purified and applied to endogenous polymerase reaction (EPR). As shown in S10 Fig, while 3TC-arrested nucleocapsids were EPR-negative, HBV DNA synthesis occurred in L-FMAU-arrested nucleocapsids, though the efficiency was slightly less than the untreated control. These results suggested that L-FMAU treatment could be used to produce replication-competent HBV RNA-containing particles.

**Fig 9:**
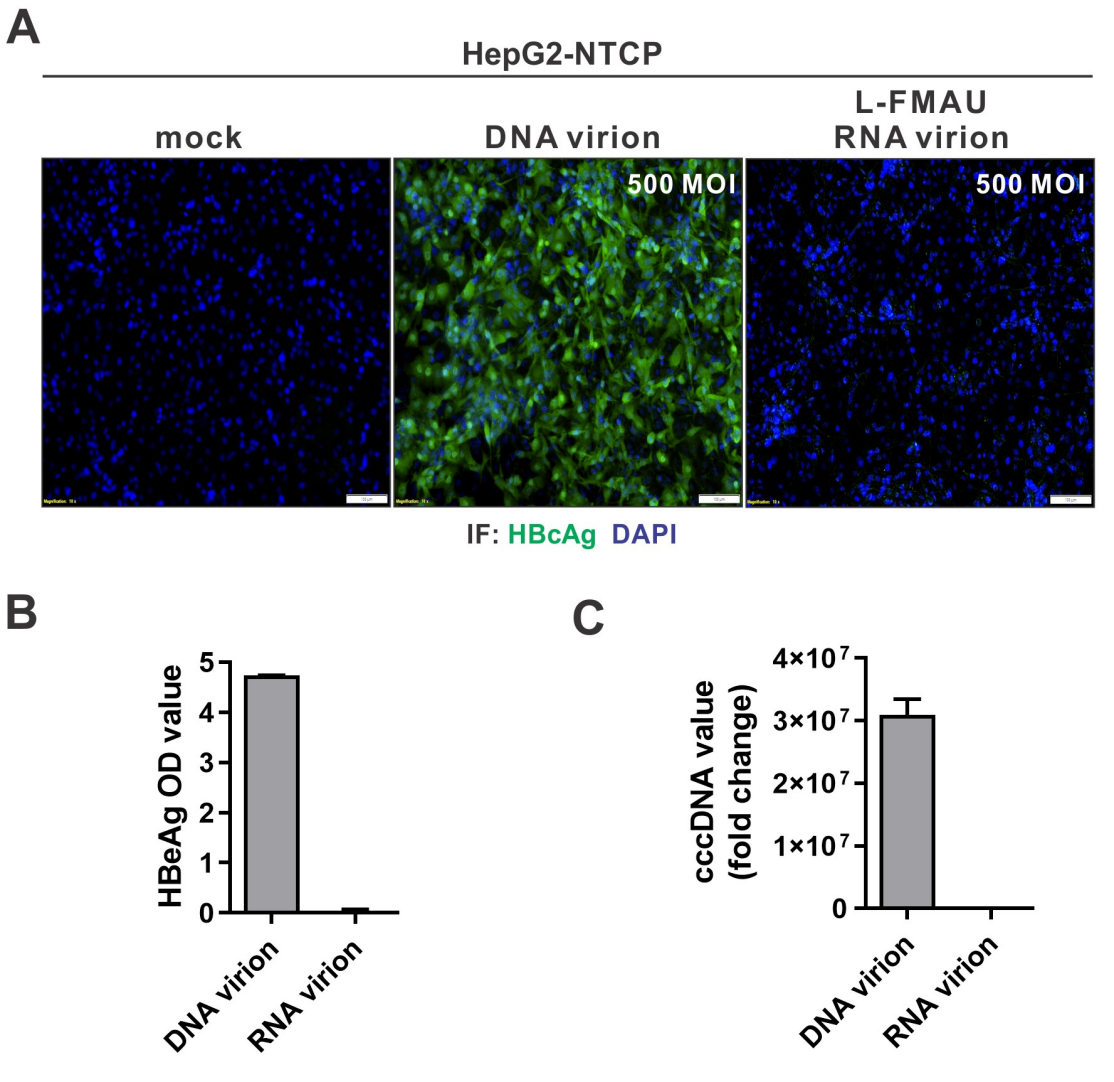
L-FMAU-arrested HBV RNA-virions are non-infectious. HepG2-NTCP12 cells in 96-well plates were infected with the wild-type HBV virions (DNA virions) and the RNA virions collected from L-FMAU-treated HepAD38 cells, respectively, at MOI of 500 vge/cell. Uninfected cells served as mock control. 10 days later, the infection was assessed by (A) intracellular HBcAg immunofluorescence, (B) supernatant HBeAg ELISA, and (C) HBV cccDNA qPCR. DAPI staining of cell nuclei served as counter staining. The intracellular cccDNA copy numbers were normalized by mitochondrial DNA and plotted as relative levels of control (fold change of 1) (mean±SD, n = 3).

Next, we collected HBV RNA-virions from induced HepAD38 cells under L-FMAU treatment (S11 Fig), and their infectivity was assessed in HepG2-NTCP cells. As shown in Fig 9, compared to HBV DNA-virions, the RNA-virions did not establish a productive infection, as evidenced by the absence of HBc, HBeAg, and cccDNA. Thus, we conclude that HBV RNA-virions are deficient for infection *in vitro*.

## Discussion

**Fig 10:**
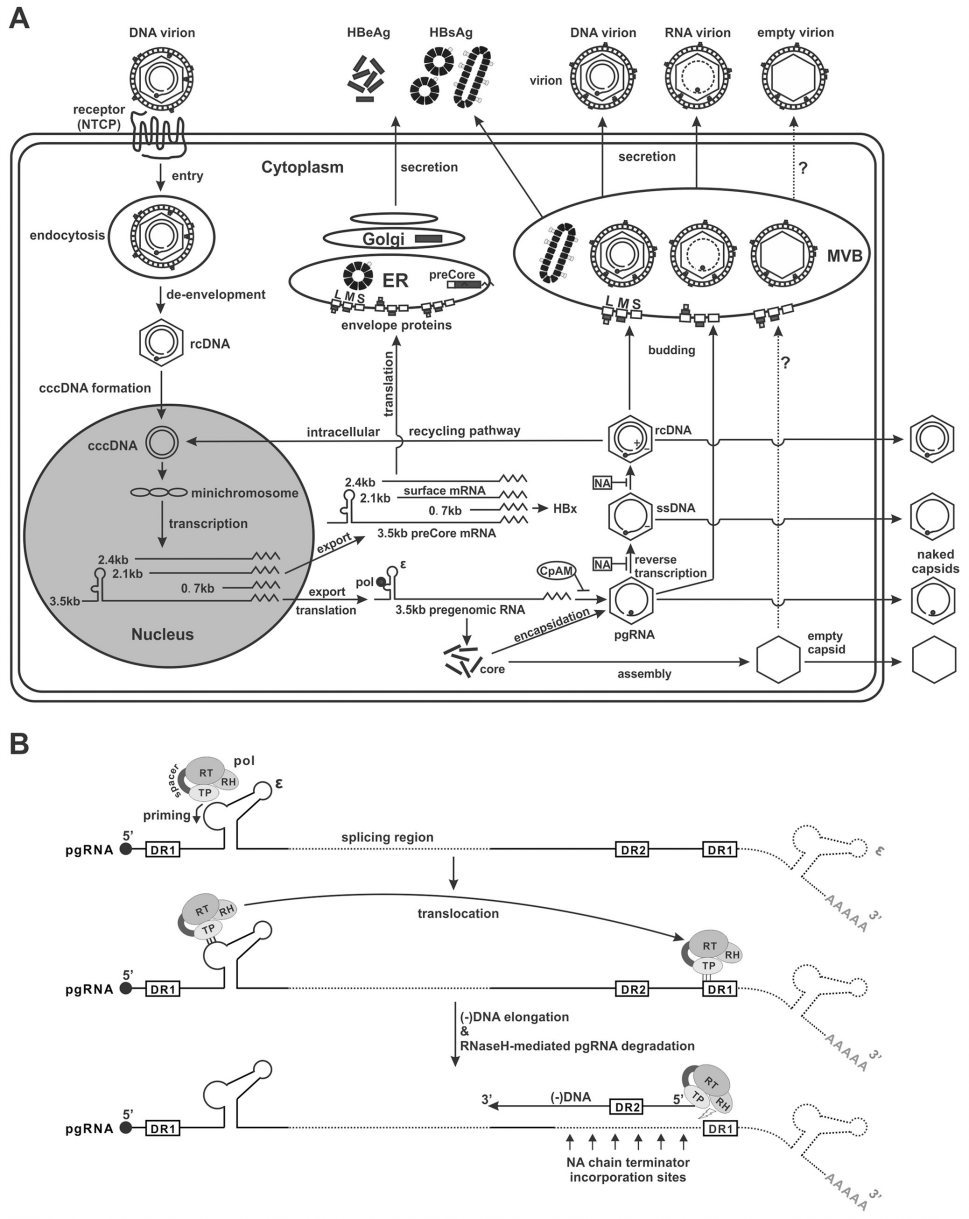
The proposed model for serum HBV RNA biogenesis. (A) The formation and secretion of HBV pgRNA-containing capsid and virion. See Introduction for a brief description of the canonical HBV replication cycle. The cytoplasmic pgRNA is encapsidated with viral polymerase (pol) into capsid, which is enveloped and secreted through cellular MVB pathway to yield the infection-deficient RNA virion, or release to the extracellular environment as naked capsid *via* an undefined secretory mechanism. Blocking intracellular viral nucleocapsid formation by CpAM diminishes the production of pgRNA-containing particles; the inhibition of viral DNA replication by NA results in intracellular accumulation of pgRNA-containing capsid and may enhance the production of extracellular RNA virions and capsids. (B) The proposed mechanism for generating truncated serum/supernatant HBV pgRNA. Viral polymerase is schematically illustrated and the four major function domains are indicated, specifically the terminal protein (TP) domain, spacer domain, reverse transcriptase (RT) domain, and RNaseH (RH) domain. The unspliced and spliced pgRNA (indicated by internal dotted line) carrying an intact 5’ epsilon motif are packaged into the capsid, with the 3’ portion downstream of DR1 being possibly left outside of the capsid and subsequently removed by cellular ribonuclease(s). Upon DNA priming catalyzed by viral polymerase at 5’ epsilon of the encapsidated pgRNA, the polymerase carries nascent DNA and translocates to the 3’ DR1 to initiate the minus-strand DNA elongation. The reverse transcription of pgRNA generates DNA:RNA hybrid and the RNA moiety is degraded by the RNaseH endonuclease activity of viral polymerase. The NA treatment stops viral DNA synthesis at various corresponding nucleotide positions downstream of DR1 due to its chain terminator effect, which results in further trimming of the 3’ end of capsid pgRNA at positions upstream of 3’ DR1 by HBV RNaseH. See text for more details.

HBV is a para-retrovirus, whose virion contains a viral DNA genome derived from reverse transcription of an intracellular RNA pregenome (pgRNA) [2]. However, HBV RNA has been found in the serum of CHB patients since the 1990s. Recent studies have suggested that the circulating HBV RNA is the pgRNA encapsidated in virion, which may serve as a new serological marker for HBV infection, treatment and prognosis [6]. In this study, we systematically studied the biogenesis and molecular characteristics of serum HBV RNA to assist the better understanding and proper application of this potential novel HBV biomarker. A model of serum HBV RNA biogenesis is proposed for the following discussion (Fig 10).

HBV replicating cells shed a plethora of viral particles with different structural and genetic components, including the infectious DNA-virions, genome-free virions, envelope-only subviral particles, and nonenveloped naked capsids (Fig 10) [4]. Based on their different physical and chemical properties, the enveloped and nonenveloped particles can be separated by the native agarose gel electrophoresis [28]. In the present study, we have provided multiple, complementary lines of evidence to delineate the origin and nature of extracellular HBV RNA. First, by making use of the HBV Y63D mutant, which is defective in viral DNA replication without affecting pgRNA encapsidation, RNA-containing virions and naked capsids were detected in the culture fluids (Fig 1). Moreover, HBV RT inhibitor 3TC also induced the secretion of the HBV RNA-virions (S1 Fig). Second, the formation of HBV RNA-virion requires both pgRNA encapsidation and viral envelop proteins, as confirmed by CpAM treatment and an envelope-null HBV mutant, respectively (Figs 2 and 3). Third, HBV RNA-virion utilizes the MVB pathway for egress, as the secretion of both the DNA- and RNA-virions can be inhibited by the DN VPS4A (Fig 4). Fourth, serum/supernatant HBV RNA are detergent- and ribonuclease-resistant (Fig 5). In conjunction with previous studies [7, 8], our results further suggest that RNA-virion is a new species of extracellular HBV particles (Fig 10).

The secretion of HBV RNA virion is an exception to the prevailing “single strand blocking” model for hepadnavirus morphogenesis, in which the genome-free capsid or mature dsDNA-containing capsid possesses a “secretion signal” to efficiently interact with viral envelope protein for empty or complete virion formation, respectively, whereas the immature ssDNA-containing capsid lacks the “secretion signal” or possesses a “blocking signal” to prevent virion formation [4, 5]. The “secretion signal” and “blocking signal” have not been well defined, but may be unrelated to the different states of capsid phosphorylation [4, 29]. However, this classical model does not apply to snow goose HBV, which secrets ssDNA-containing virion as efficiently as virions containing mature dsDNA [30]. The HBV RNA virion thus represents another exception to this existing model. It is possible that pgRNA-containing capsid may possess a similar “packaging signal” with the dephosphorylated mature DNA capsid, as a recent study suggested that capsid dephosphorylation occurs during pgRNA encapsidation [31]. Interestingly, blocking HBV reverse transcription by Y63D mutant could enhance the production of RNA-virion (Figs 1 and 2), which indicates that the accumulation of pgRNA-containing capsid may overcome the spatial or temporal restriction of “single strand blocking” mechanism, leading to RNA-virion morphogenesis and subsequent MVB-dependent egress (Fig 4). Further study is needed to delineate the detailed structural and molecular profile of pgRNA-containing capsid and its interaction with envelope proteins. In addition, the secretion efficiency of pgRNA-virion should be determined and compared to DNA virion.

Besides RNA-virion, the supernatant HBV RNA exists predominantly in naked capsid in cell cultures (Figs 1–4 and S1 Fig). The naked capsids in cell culture fluids are empty or contain all types of HBV replicative intermediates [5, 21, 32, 33]. The release of naked capsid is independent of ESCRT/MVB secretory pathway (Fig 4), but previous studies have suggested that an ESCRT-0 component HGS and the ESCRT-III-binding protein Alix could direct the egress of naked capsids in cell cultures [21, 33]. However, the detailed secretion pathway for naked capsid remains elusive. The free naked capsid has not been detected in HBcAb-negative CHB patient serum [34], however, a recent study demonstrated that serum HBV RNA were predominantly present in the form of capsid-antibody complexes (CACs) [8]. With such controversies, it is of interest to study whether naked capsids could be secreted spontaneously *in vivo*, whether the capsid in CACs is derived from de-enveloped virion, and why the circulating CACs are not rapidly eliminated by dendritic cells and macrophages. On the other hand, the persistence of RNA-virions in CHB patients, similar to DNA-virions, is likely due to the lack of neutralizing surface antibodies.

Consistent with previous studies [8, 14, 15, 24], Northern blot assay confirmed that the serum/supernatant HBV RNA are predominantly short pgRNA species of heterogeneous lengths (Fig 5). Profiling of HBV RNA by RT-PCR further demonstrated that a substantial amount of spliced pgRNA variants exist in both the culture supernatant and patient serum samples. Sequencing analysis showed that these variants contained internal deletions of various lengths (Fig 6 and S3 Fig), which match the reported intracellular spliced pgRNA [22, 23]. Interestingly, the splicing pattern varies between cell culture supernatant and patient serum RNA samples and between samples from HBV transiently- and stably-transfected cells. Such discrepancies might be due to the genetic difference between HBV genotypes and/or host backgrounds. Time is likely another regulatory factor as unspliced supernatant pgRNA was only found in HBV transiently-transfected HepG2 cells but not in HepAD38 cells with longer induction (S3 Fig). Nonetheless, the Sp1 variant, which lacks an intron starting within the core gene and ending in the middle of the S gene on pgRNA, was found in both culture supernatant and patient serum (Fig 6 and S3 Fig). The role of spliced pgRNA variants in HBV lifecycle is largely unknown. Previous studies have shown that the encapsidated Sp1 can be reverse transcribed into a dsDNA and secreted in virion form as a defective HBV particle [35]; and two novel proteins encoded by Sp1, specifically HBSP and a N-terminally truncated polymerase, negatively regulate IFN-α signaling [23]. The mechanism for the selective secretion of spliced pgRNA-containing virion awaits investigation. A plausible explanation could be that, the spliced pgRNA, if encapsidated, forms different secondary or tertiary structure and reverse transcribes less efficiently than the unspliced pgRNA, resulting in premature nucleocapsid envelopment and secretion as RNA-virion. This notion is consistent with the above explanation for the enhanced production of RNA-virion by inhibiting HBV polymerase activity.

In addition to pgRNA splicing, 3’ truncations also contribute to the short heterogeneous lengths of supernatant/serum HBV RNA. Using 3’ RACE analysis, we have mapped the 3’ termini of NA-arrested supernatant/serum HBV pgRNA predominately at a clustered region upstream of 3’ DR1, the minus strand DNA elongation initiation site after polymerase priming (Fig 7). The truncation of pgRNA at the heterogeneous sites upstream of 3’ DR1 is attributed to the degradation of viral RNA in the RNA:DNA duplex catalyzed by the RNaseH activity of HBV polymerase during minus strand DNA synthesis (S7 Fig and Fig 10B). The above reactions are fast-initiated, highly efficient and interlocking, which result in a great heterogeneity of pgRNA 3’ termini during reverse transcription [8]. In the presence of NAs, the incorporation of nucleotide analogue into the elongating chain of HBV minus strand DNA at the base pairing positions corresponding to pgRNA template terminates DNA synthesis and blocks further RNaseH-mediated degradation of pgRNA. However, NAs do not efficiently block minus strand DNA synthesis at the early stage [36], giving rise to truncated virion pgRNA with 3’ ends located between DR1 and DR2 or even upstream of DR2 (Figs 7–10). Interestingly, the 3TC-arrested virion RNA are slightly shorter than the 3TC-arrested naked capsid RNA (Fig 7C and 7D), which might be due to that the envelopment of nucleocapsid blocks the influx of 3TC-triphosphate during virion morphogenesis. In addition, the supernatant HBV RNA of replication-defective Y63D mutant possess heterogeneous 3’ truncations up to the 3’ DR1 in the absence of RNaseH activity (S7 Fig), indicating that, similar to DHBV [25], the 3’ end of HBV capsid pgRNA sequence downstream of DR1, which is not used as template for reverse transcription, is excluded from being packed into the interior of capsid and thus susceptible to intracellular and/or extracellular host ribonuclease(s). In this regard, host ribonuclease(s) may also be involved in processing the 3’ end of serum HBV RNA, though the effect is likely masked by the viral RNaseH activity (Fig 10). Further study is needed to precisely define the spatial location and characteristics of 3’ end of HBV pgRNA in the process of pgRNA encapsidation.

Considering that pgRNA is directly and exclusively transcribed from cccDNA, theoretically, the serum HBV pgRNA represents a better serological marker for cccDNA activity than viral DNA, especially in the NA-suppressed CHB patients. Indeed, serum HBV RNA has been used in clinical studies to monitor intrahepatic cccDNA activity and guide the cessation of NA treatment [6, 37], and we have recently reported that the dynamics of signature-mutations of serum pgRNA could serve as a biomarker for cccDNA turnover in NA-treated patients [38]. The developed detection means of serum HBV RNA are mainly RT-PCR-based [6]. Considering that the supernatant/serum HBV RNA are predominantly spliced and 3’ truncated, to achieve a better specificity and coverage of serum HBV RNA species with RT-PCR, the consolidated results from previous studies and the present study infer that the RT primer should target the sequence upstream of DR2, and the PCR amplicon should be located between the 3’ splicing site and RT primer site. In addition, single molecule direct sequencing approach is needed to completely profile the complexity of serum HBV RNA species and aid the development of detections with better coverage.

The existence of extracellular HBV RNA-virion has raised up a valid question on whether it is an infectious particle. Due to the similar protein composition and density between HBV RNA-virion and DNA-virion, it is technically challenging to purify RNA virions. Though inhibition of HBV polymerase could selectively enrich the production of RNA-virions in cell cultures (Fig 1 and S1 Fig), the 3TC- or entecavir-arrested RNA virions are replication-incompetent and therefore noninfectious due to the irreversible DNA chain termination effect of drugs (Fig 8 and S9 and S10 Figs) [24, 26]. In this study, we used L-FMAU, a thymidine analogue that inhibits HBV DNA replication mechanistically distinct from other NAs [39], to produce RNA virions. L-FMAU blocks both HBV protein priming and DNA elongation in a non-competitive manner and without being incorporated into the viral DNA [27], rendering a reversible replication capacity of the arrested HBV nucleocapsids in cells and *in vitro* (Fig 8 and S10 Fig). However, the L-FMAU-arrested RNA-virions remain incapable of infecting HepG2-NTCP cells even at high MOI (Fig 9). While it is possible that there were residual L-FMAU molecules in the virion, it is also conceivable that the spliced pgRNA variants might not be efficiently converted into rcDNA and cccDNA. Furthermore, the suboptimal efficiency of *in vitro* HBV infection system might have limited the assessment of infectivity of L-FMAU-arrested RNA-virions. It remains possible that, under *in vivo* natural infection, a small portion of HBV pgRNA virions, especially those containing unspliced pgRNA, may be infectious but unlikely contribute significantly to virus spread. Nonetheless, the current results suggest that the HBV RNA-virion does not essentially blur the distinction between para-retroviruses and conventional retroviruses. Future study should be carried out to specifically evaluate the infectivity of unspliced pgRNA- and spliced pgRNA-containing virions *in vitro* and *in vivo*, and to determine the biological functions of these RNA-virions, including the HBx mRNA-containing virions [15, 40], in HBV lifecycle and virus-host interactions.

## Materials and methods

### Cell lines

Huh7 and HepG2 cells were cultured in DMEM/F12 medium (Corning) supplemented with 10% fetal bovine serum, 100 U/ml penicillin and 100 μg/ml streptomycin. The HepG2-based, tetracycline-inducible HBV stable cell lines HepAD38 and HepDES19 were established previously [41, 42], and maintained in the same way as HepG2, but with the addition of 1 μg/ml tetracycline (tet). To induce HBV replication and virus particle secretion in HepAD38 or HepDES19 cells, tet was withdrawn from the culture medium, and the cells were cultured for the indicated time periods. The HepG2-NTCP12 cell line supporting HBV infection was described previously [43].

### HBV inhibitors

AT-61 was synthesized by Pharmabridge. SBA-R01 and GLS-4 were kindly provided by Arbutus Biopharma. Lamivudine (3TC) and clevudine (L-FMAU) were kindly provided by Dr. William Mason (Fox Chase Cancer Center).

### HBV serum samples

Genotype B, C and E HBV-positive, treatment-naive patient sera were purchased from SeraCare. Telbivudine-treated genotype C HBV patient serum was previously collected from a single patient during the EFFORT study (registration number NCT00962533) [44].

### Ethics statement

All samples from HBV-infected patients used in this study were previously collected and de-identified. This study was conducted in compliance with the ethical guidelines of the 1975 Declaration of Helsinki and was approved by the IRB Committee of Indiana University (IRB# 1808003516).

### Plasmids and transfection

HBV replication-competent plasmids, pHBV1.3 (genotype C and D) and pCMVHBV (genotype D), which the transcription of pgRNA is governed by the viral core promoter and human cytomegalovirus immediate-early (CMV-IE) promoter, respectively, were described previously [45]. Plasmid pCMVHBVΔPol contains a start codon mutation (ATG to ACG) of the pol ORF in pCMVHBV backbone to block the expression of pol. Plasmid pCMVHBV-Y63D bears a Y63D substitution in the terminal protein domain of HBV pol gene to block DNA synthesis but still allows pgRNA encapsidation (a gift from Dr. Jianming Hu) [5]. To obtain HBV genome that is unable to produce the three envelope proteins, two tandem stop codons (217TTGTTG222 to 217TAGTAG222; mutations are underlined) were introduced into the amino terminus of the small (S) envelope protein ORF in plasmid pCMVHBV-Y63D by using the Q5 Site-Directed Mutagenesis Kit (NEB) to yield plasmid pCMVHBV-Y63DΔS. The plasmids expressing C-terminally GFP-tagged wild-type (wt) and dominant-negative (DN) mutant (E228Q) human VPS4A protein were provided by Drs. Wesley Sundquist and Reinhild Prange. Cells were transfected with indicated plasmid(s) by Lipofectamine 3000 (Life Technologies) according to the manufacturer's directions.

### HBV nucleic acids analyses

HBV total RNA, cytoplasmic encapsidated HBV pgRNA and core DNA were extracted and subjected to Northern and Southern blotting as described previously [46]. Hybridization signals were recorded on a phosphorimager screen and scanned by the Typhoon FLA-7000 imager (GE Healthcare). HBV cccDNA was extracted by a modified Hirt DNA extraction method [47], and quantified by qPCR and normalized to mitochondrial DNA according to our previous publication [48].

### HBV particle gel assay

The intracellular HBV capsids were analyzed by capsid gel assay as previously described [32, 49]. The extracellular HBV particles (virions, subviral particles, and naked capsids) and their HBV DNA content were analyzed by particle gel assay according to a published protocol [28]. To detect viral particle-associated HBV RNA, the particle gel membrane was transiently treated with the denaturing buffer containing 0.5 M NaOH and 1.5 M NaCl for 20 s, followed by neutralization with a solution containing 1 M Tris-HCl (pH 7.4) and 1.5 M NaCl for 5 min, and hybridized with an [α-^32^P]-UTP-labeled plus-strand-specific full-length HBV riboprobe.

### Western blot assay

Whole cell lysate samples prepared by Laemmli buffer was resolved in a 12% SDS-PAGE and proteins were transferred onto Immobilon PVDF-FL membrane (Millipore). The membranes were blocked with Western Breeze blocking buffer (Life Technologies) and probed with antibodies against HBc [50], VPS4 (Santa Cruz, clone C-12), or β-actin (Santa Cruz). Bound antibodies were revealed by IRDye secondary antibodies. The immunoblot signals were visualized and quantified with the Li-COR Odyssey system.

### Sucrose density gradient centrifugation and dot blot

The supernatant of 3TC-treated HepAD38 cells was mixed with PEG-8000 powder (final concentration (wt/vol) of 10%) and gently rotated at 4°C overnight, HBV particles were then precipitated by centrifugation at 1,000×g for 30 min at 4°C and re-dissolved in 1×TNE buffer. Gradients were formed by overlaying 3 ml of 5%, 10%, 15%, 20%, 25%, 30%, 35%, 40%, 45%, 50%, and 55% (wt/vol) sucrose solutions in TNE buffer and incubated at 4°C for 10 h to allow gradient to become continuous. Then, five milliliters of the prepared HBV particle solution were overlaid on the gradients. Samples were centrifuged in Beckman SW28 rotor at 26,000 rpm for 16 h at 4°C. Twenty-two fractions of approximately 1.7 ml were collected from the bottom of the tube.

To detect HBcAg and HBsAg, 100 μl of each fraction was dot-blotted on nitrocellulose membrane and probed with the corresponding antibody (Dako), the bound antibody was detected with IRDye secondary antibodies by using Li-COR Odyssey Fc System. For detection of particle-associated HBV DNA, the dot blot membrane was soaked in denaturing solution containing 0.5 N NaOH and 1.5M NaCl for 15min, followed by neutralization with a solution containing 1 M Tris-HCl (pH7.6) and 1.5 M NaCl for 5 min. HBV DNA was detected by hybridization with an [α-^32^P]-UTP-labeled HBV minus-strand-specific riboprobe. For detection of particle-associated HBV RNA, the dot blot membrane was transiently treated by the above-mentioned denaturing buffer for 20 s, followed by neutralization for 5 min. HBV RNA was detected by hybridization with an [α-^32^P]-UTP-labeled HBV plus-strand-specific riboprobe.

### Multiple identification PCR

Total RNA were extracted from cell and supernatant/serum samples by TRIzol and then digested with or without DNase I, followed by purification with RNA Clean & Concentrator (Zymo Research). To ensure that the residual HBV DNA, if any, was completely eliminated by DNase I digestion, all the DNase I-treated samples were confirmed to be HBV DNA PCR-negative without the reverse transcription step. The purified RNA was reverse transcribed using an HBV-specific RT primer or oligo(dT)20. The primers for amplifying HBV pcRNA, pgRNA, and spliced pgRNA are listed in S1 Table. The PCR was performed by using LA-Taq DNA polymerase (TaKaRa) in the following conditions: the reaction mixture was denatured at 98°C for 1 min, followed by 35 cycles at 98°C for 30 s, 55°C for 15 s, 68°C for 2 min 30 s, and a final extension at 68°C for 2 min. PCR DNA fragments of varying lengths were resolved by a 1.5% agarose gel. When indicated, the PCR products were purified by using QIAquick Gel Extraction Kit (Qiagen) and cloned into the StrataClone PCR Cloning Vector (Agilent) for Sanger sequencing.

### 3’ RACE of HBV RNA

HepAD38 and HepDES19 cells were seeded into T75 flask at a density of 9.0×10^6^ cells per flask and cultured for an additional two days. Cells were then treated with 10 μM of 3TC for 18 days in the absence of tet. The cells were lysed with TRIzol reagent and total cellular RNA was extracted according to the standard protocol. The HBV patient serum RNA, total cell culture supernatant RNA, and the cell culture-derived virion RNA collected by sucrose gradient separation were extracted with TRIzol LS reagent. The extracted RNA were digested by DNase I and further purified by RNA Clean & Concentrator (Zymo Research), and the flow through RNA were polyadenylated with a poly(A) Tailing Kit (Thermo Scientific) by following the manufacturer's direction. The resulting RNA was reverse-transcribed into cDNA by using SuperScript III Reverse Transcriptase with oligo(dT) adaptor primer (AP, Invitrogen) 5’-GGCCACGCGTCGACTAGTAC(dT)17-3’. The primers for nested PCR amplification of the DNA complementary to the 3’ terminal fragments of the HBV RNA are user-designed Gene-Specific Primer 1 (GSP1, 1^st^ round), GSP2 (2^nd^ round) and the Abridged Universal Amplification Primer (AUAP, Invitrogen) (S1 Table). The PCR was performed by using LA-Taq DNA Polymerase (TaKaRa) in the following conditions: 98°C, 1 min; 98°C, 30 s, 58°C 15 s, 68°C 30 s, 30 cycles; 68°C 2 min. The PCR products were purified by using QIAquick Gel Extraction Kit and cloned into the StrataClone PCR Cloning Vector. Approximately 30 positive viral cDNA clones were sequenced to determine the 3’ terminus of the encapsidated viral RNA.

### HBV infection

HBV wild-type infectious particles were collected from the supernatant of induced HepAD38 cells, HBV RNA particles were collected from the 3TC- or L-FMAU-treated HepAD38 cells upon induction, HBV naked capsids were collected from the supernatant of untreated and 3TC-treated HepDES19 cells upon induction. The infection of HepG2-NTCP12 cells, intracellular HBc immunofluorescence microscopy and HBeAg ELISA were conducted as previously published [51].

### HBV endogenous polymerase reaction (EPR)

The EPR of cytoplasmic HBV nucleocapsid was conducted according to a published literature with modifications [50]. Briefly, untreated and NA-treated HepAD38 cells (tet-) in 12-well-plate were lysed by 200 μl of lysis buffer containing 10 mM Tris-HCl (pH 8.0), 10 mM EDTA, 1% NP-40, and 2% sucrose at 37°C for 10 min, cell debris and nuclei were removed by centrifugation. The HBV capsids in clarified cytoplasmic lysate were precipitated down by 10% PEG-8000, the pellet was dissolved in 200 μl TNE buffer and mixed with 200 μl of 2× EPR buffer which consisted of 0.3 M NaCl, 0.1 M Tris-HCl (pH 8.0), 20 mM MgCl2, 2 mM dithiothreitol, 0.2% (vol/vol) Nonidet P-40, and 0.2 mM of deoxynucleoside triphosphates (dNTPs). After incubation at 37°C for the indicated period of time, total viral DNA in the EPR samples were extracted by adding equal volumes of HBV core DNA extraction buffer that contained 20 mM EDTA, 20 mM Tris-HCl (pH 8.0), 0.2% sodium dodecyl sulfate (SDS), and 1 mg/ml pronase, followed by incubation for 1 h at 37°C. The digestion mixture was extracted twice with phenol, and DNA was precipitated with ethanol and dissolved in TE buffer (10 mM Tris-HCl [pH 8.0], 1 mM EDTA), followed by Southern blot analysis.

## Supporting information

S1 Fig The presence of HBV RNA virion-like-particles in HepAD38 cell culture fluid when HBV reverse transcription is inhibited by 3TC. HepAD38 Cells were seeded in 35-mm dishes and cultured in the presence of tetracycline (tet) (1 μg/ml) until cells became confluent, and tet was then removed from culture medium to induce HBV replication. One group of cells were mock treated with DMSO, another group of cells were treated with 10 μM of 3TC simultaneously upon tet withdrawal. Fresh culture media with or without 3TC were replenished at 2-day intervals. Cells continued to be cultured in tet-free medium for 14 days. Cells and culture fluids were harvested at the indicated time points after tet removal. (A) Cells were harvested at day 8 after withdrawal of tet, the intracellular total viral RNA and encapsidated pgRNA were analyzed by Northern blotting. (B) Cytoplasmic core DNA accumulated at indicated time points were analyzed by Southern blotting (upper panel), the viral DNA and RNA in extracellular HBV virions and naked capsids at indicated time points were detected by particle gel assay using (-) strand-specific probe (middle panel) and (+) strand-specific probe (lower panel), respectively.
(TIF)

S2 Fig Nuclease treatment of HBV nucleic acids extracted from cell culture fluid. The supernatant of induced HepAD38 (tet-) were either untreated or treated with NP40 (final concentration of 0.5%) for 10 min at room temperature, followed by MNase (20 units/μl) digestion in the presence of 5 mM CaCl2 for 15 min at 37°C or remain untreated. Viral DNA/RNA were co-purified by QIAamp MinElute Virus Spin Kit and subjected to DNaseI digestion alone, DNaseI plus RNaseA double digestion, or remain untreated. After digestion, the samples were subjected to Southern blotting (A) and Northern blotting (B) with (-) strand- and (+) strand-specific HBV probes, respectively. 5 μg of total RNA from pCMVHBV-transfected HepG2 cells served as a control for intracellular HBV RNA.
(TIF)

S3 Fig Supernatant HBV RNA are mainly spliced pgRNA in HBV-transfected cells. (A) Schematic illustration of the reported spliced pgRNA (Sp) variants. The full-length pgRNA and the overlapping ORFs are shown on the top, the Sp variants are aligned underneath, and introns are indicated with dotted lines. The numbering of Sp variants is according to literature [14, 22, 23]. Sp22 is a novel putative splicing variant (Fig 6C). (B) HepG2 cells were transfected with genotype D HBV plasmid pHBV1.3-gtD for 5 days. The pgRNA Sp variants of intracellular and supernatant RNA were analyzed by RT-PCR using genotype D-specific RT and pgRNA splicing primers (S1 Table). The distinct amplicon bands were cloned and sequenced, their corresponding Sp variants were indicated. FL: full-length amplicon from unspliced pgRNA. (C) HepAD38 cells were induced and treated with or without 3TC for the indicated time durations, the intracellular and extracellular Sp variants were determined by RT-PCR and sequencing.
(TIF)

S4 Fig Preparation of HBV RNA-containing naked capsids from the supernatant of 3TC-treated HepDES19 cells. HepDES19 Cells were cultured in the presence of tet (1 μg/ml) until cells became confluent, and tet was then removed from culture medium to induce pgRNA transcription. One group of cells were treated with DMSO and the other group of cells were treated with 10 μM of 3TC simultaneously with tet withdrawal. Cells were harvested at day 14 after withdrawal of tet. (A) Intracellular total viral RNA and encapsidated pgRNA, core DNA were analyzed by Northern and Southern blot, respectively. (B) Extracellular HBV DNA- and RNA-containing capsids were analyzed by particle gel and hybridization.
(TIF)

S5 Fig Sucrose gradient separation and analysis of viral particles from 3TC- treated HepAD38 cells. (A) Distribution of intracellular HBV particle-associated core protein and DNA in sucrose gradient. HBV particles prepared from the lysate of 3TC-treated HepAD38 cell were overlaid on a 5% to 55% (wt/wt) sucrose gradient for ultracentrifugation separation. Fractions were collected from bottom to top and spotted onto nitrocellulose membrane, followed by immunoblotting of HBV capsids with anti-HBcAg antibodies. The nitrocellulose membranes were then denatured for HBV DNA hybridization. (B) Distribution of extracellular HBV particle-associated antigens and DNA/RNA in sucrose gradient. HBV particles were concentrated from 3TC-treated HepAD38 cell culture supernatant by PEG-8000, and were layered over a 5% to 55% (wt/wt) sucrose gradient for ultracentrifugation separation. Fractions were collected from bottom to top and spotted onto a nitrocellulose membrane. HBV envelope and core proteins were detected by immunoblotting with anti-HBsAg and anti-HBcAg antibodies. Virus particle-associated DNA/RNA were detected by hybridization as described in Materials and Methods. Fractions 9–12 were collected as 3TC-arrested RNA virions.
(TIF)

S6 Fig Serum HBV RNA are mainly devoid of poly(A) tail. Serum RNA purified from the genotype C HBV-infected CHB patient were subjected to RT-PCR with the indicated primers (A). The PCR products were resolved by electrophoresis and stained by ethidium bromide (B).
(TIF)

S7 Fig Mapping the 3’ terminus of supernatant HBV-Y63D pgRNA. HepG2 cells in 6-well plate were transfected with the pCMVHBV-Y63D (Y63D) for 5 days. The extracellular total HBV RNA was analyzed by 3’ RACE and clone sequencing as described in Materials and Methods. The positions of DR1, DR2, and gtD HBV gene specific primers for nested PCR (GSPD1 and GSPD2) are indicated. The nucleotide positions and numbers of mapped 3’ termini of HBV RNA are indicated with solid dots underneath the illustrated full-length pgRNA and listed in the table underneath. The RNA molecules with 3’ end position mapped at nt1937 are polyadenylated.
(TIF)

S8 Fig Northern blotting of intracellular HBV total RNA and encapsidated pgRNA. HepG2 cells in 12-well plate were transfected with 1.6 μg of pHBV1.3. Cells were harvested at day 5 post-transfection and the intracellular viral total RNA and encapsidated pgRNA (capsid pgRNA) were extracted and analyzed by Northern blotting as described in Materials and Methods. 5 μg of total RNA (lane 1) and the capsid pgRNA extracted from one well of a 12-well plate (lane 2) were hybridized with a plus (+) strand-specific full length HBV riboprobe. Ribosomal RNA (28S and 18S rRNA) served as total RNA loading control (lane 1), and the loss of rRNA indicated a complete digestion of unencapsidated RNA by MNase (lane 2). HBV 3.5kb, 2.4kb, 2.1kb RNAs, and capsid pgRNA are labeled. A longer exposure of the blot is included.
(TIF)

S9 Fig HBV particles produced under 3TC treatment were unable to infect HepG2-NTCP cells. (A) HepG2-NTCP cells were left uninfected (mock) or infected with HBV particles collected from the supernatant of induced HepAD38 cells or HepDES19 cells with or without 3TC treatment at indicated MOI. The composition of viral particles in each inoculum was indicted by illustrations. At day 10 post-inoculation, the expression of intracellular HBcAg was analyzed by immunofluorescence. (B) HBeAg in the supernatant was detected by ELISA.
(TIF)

S10 Fig The L-FMAU-arrested HBV nucleocapsid is replication-competent in EPR. Cytoplasmic HBV nucleocapsids were purified from untreated and 3TC- or L-FMAU-treated HepAD38 cells, and subjected to EPR assay as described in Materials and Methods. The reaction was terminated at indicated time points and HBV core DNA were analyzed by Southern blot hybridization.
(TIF)

S11 Fig The presence of HBV RNA virion-like particles in cell culture fluid of L-FMAU-treated HepAD38 cells. Upon the withdrawal of tet, HepAD38 cells were treated with 40 μM of L-FMAU simultaneously. L-FMAU treatment was replenished at 2-day intervals for 16 days. Cell culture fluids were harvested at the indicated time points after tet removal. The extracellular accumulation of HBV RNA virion and capsid particles were analyzed by particle gel assay and hybridization with (+) strand-specific HBV probe.
(TIF)

S1 Table Primers used in the study. (DOCX)
