# Correction: Biogenesis and molecular characteristics of serum hepatitis B virus RNA

**DOI:** 10.1371/journal.ppat.1014527

**Published:** 2026-08-20

**Authors:** Sheng Shen, Zhanglian Xie, Dawei Cai, Xiaoyang Yu, Hu Zhang, Elena S. Kim, Bin Zhou, Jinlin Hou, Xiaoyong Zhang, Qi Huang, Jian Sun, Haitao Guo

After this article [1] was published, co-corresponding author HG notified PLOS of errors in Figs 3A and S9A. Specifically:

- The rRNA panel in Fig 3A overlaps with the first four lanes in the rRNA panel of Fig 2A.
- In Fig S9A, the RNA capsid from HepDES19 (tet-, 3TC+), 600MOI panel partially overlaps with the RNA and DNA capsid from HepDES19 (tet-), 600MOI panel.

Further editorial review noted vertical discontinuities between lanes 6–7 and 9–10 of Fig 5B and lanes 1–2 of Fig S6B.

The author stated that the rRNA panel of Fig 3A and the RNA and DNA capsid from HepDES19 (tet-), 600MOI panel of Fig S9A are incorrect and provided corrected versions of Figs 3 (S1 File) and S9. They further stated that the HepAD38 (tet-) lanes in Fig 5B (lanes 7–9) were obtained from a second gel generated as part of the original experiments and were arranged in Fig 5B to match the loading order in Fig 5A to facilitate qualitative visualization and comparison. The author stated that all lanes shown in Fig S6B originate from the same gel and were placed adjacent to one another for qualitative visualization and comparison purposes. Corrected versions of, and captions for, Figs 5 and S6, with splice lines indicated with vertical black lines, are provided with this notice.

The original images underlying all panels in Figs 2A, 3A, 5B, S6B and S9A have been included with this notice as S2 File. At the authors’ request, the first four lanes of the original underlying blots associated with Fig 3A have been removed. The original quantitative data underlying Figs 9 and S9 have been included as S3 File.

**Fig 5:**
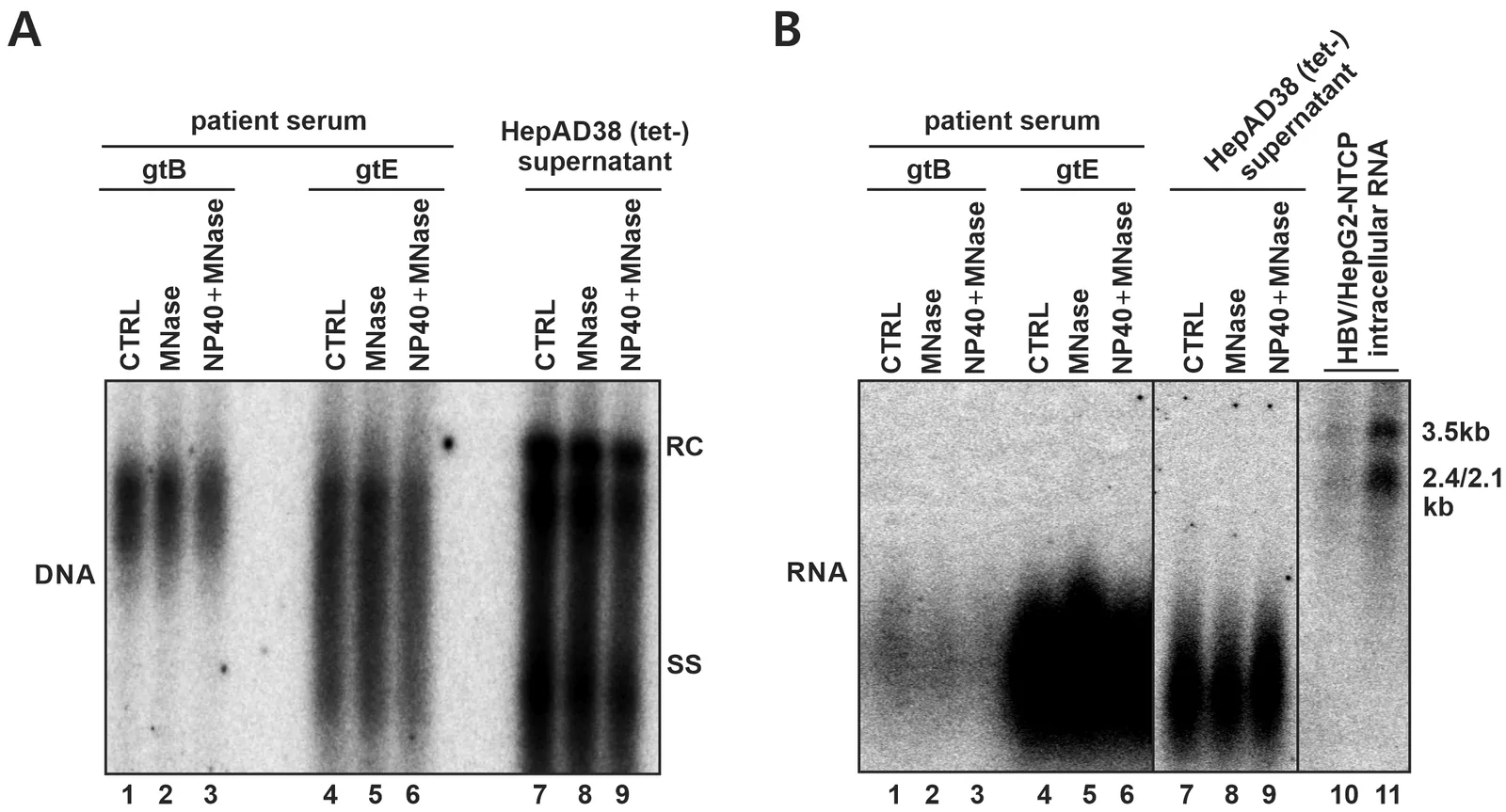
Supernatant/serum HBV RNA are short heterogeneous RNA species. Naïve HBV genotype (gt) B and E patient sera and the supernatant of induced HepAD38 (tet-) were either untreated or treated with NP40 (final concentration of 0.5%) for 10 min at room temperature, followed by micrococcal nuclease (MNase, 20 units/μl) digestion in the presence of 5mM CaCl2 for 15 min at 37°C or remain untreated. Viral DNA/RNA were co-purified by QIAamp MinElute Virus Spin Kit and analyzed by Southern blotting **(A)**. After DNase I digestion, the samples were subjected to Northern blot analysis **(B)**. 100 ng and 500 ng total RNA from gtD HBV infected HepG2-NTCP cells (MOI of 100 viral genome equivalent (vge), 4 days post-infection) served as positive control of intracellular HBV RNA. Southern blot and Northern blot were hybridized with (-) strand- and (+) strand-specific HBV probe, respectively. In **(B)**, lanes 7-9 originate from a separate gel; vertical black lines are used to indicate the splice lines.

## Supporting information

S6 Fig Serum HBV RNA are mainly devoid of poly(A) tail. Serum RNA purified from the genotype C HBV-infected CHB patient were subjected to RT-PCR with the indicated primers (A). The PCR products were resolved by electrophoresis and stained by ethidium bromide (B). In (B), lanes 1 and 2 originate from separate regions of the same gel; a vertical black line is used to indicate the splice line.
(TIF)

S9 Fig HBV particles produced under 3TC treatment were unable to infect HepG2-NTCP cells. (A) HepG2-NTCP cells were left uninfected (mock) or infected with HBV particles collected from the supernatant of induced HepAD38 cells or HepDES19 cells with or without 3TC treatment at indicated MOI. The composition of viral particles in each inoculum was indicated by illustrations. At day 10 post-inoculation, the expression of intracellular HBcAg was analyzed by immunofluorescence. (B) HBeAg in the supernatant was detected by ELISA.
(TIF)

S1 File Corrected Fig 3. (TIF)

S2 File Original images underlying all panels in Figs 2A, 3A, 5B, S6B and S9A. (ZIP)

S3 File Original quantitative data underlying Fig 9 and Fig S9. (ZIP)
