# Supplementary figures and images for: Biogenesis and molecular characteristics of serum hepatitis B virus RNA
Source: PLoS Pathog. 2020 Oct 20;16(10):e1008945. doi: 10.1371/journal.ppat.1008945 (PMC7575114; doi:10.1371/journal.ppat.1008945)

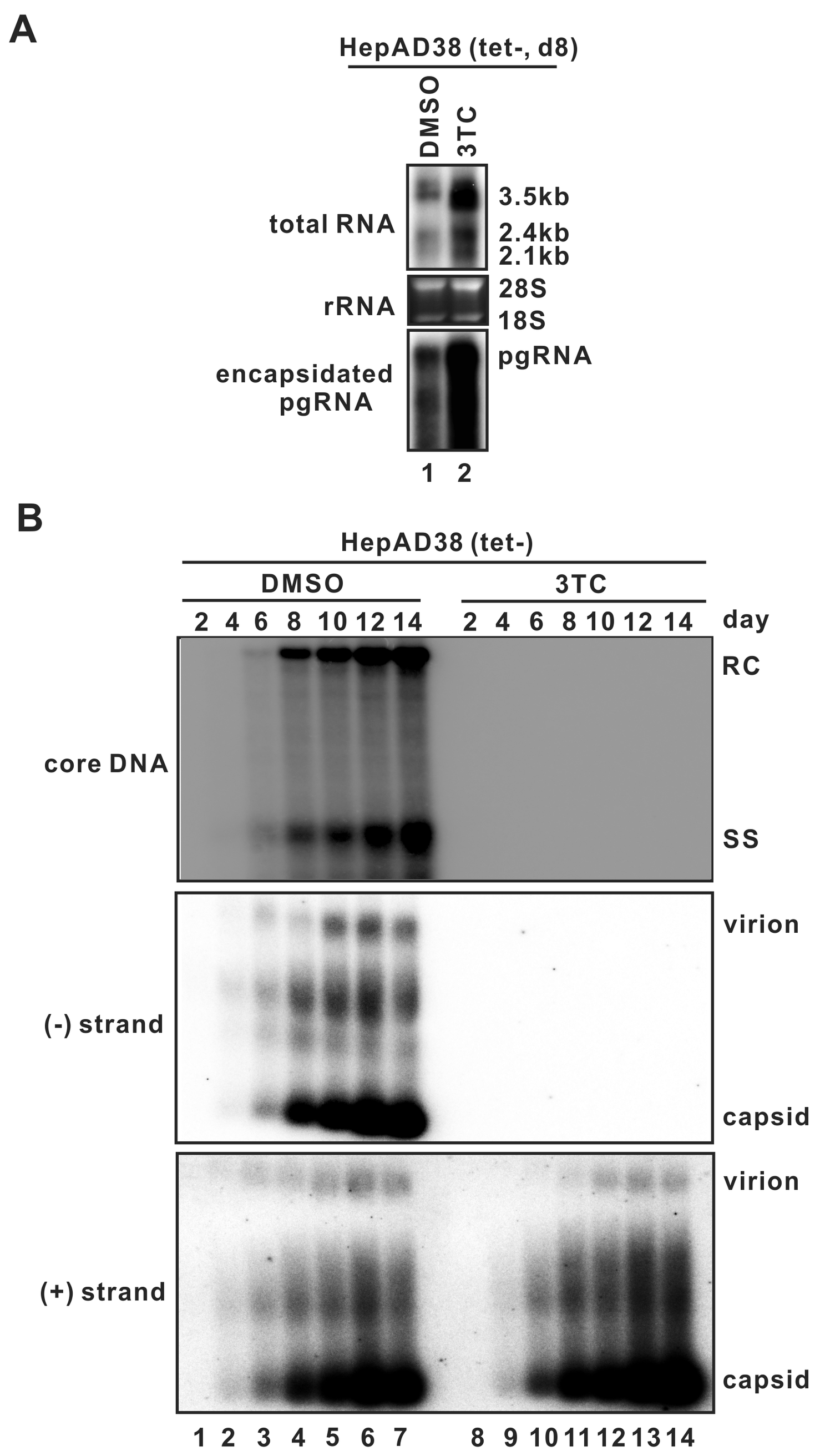

Supplement: S1 Fig — HepAD38 Cells were seeded in 35-mm dishes and cultured in the presence of tetracycline (tet) (1 μg/ml) until cells became confluent, and tet was then removed from culture medium to induce HBV replication. One group of cells were mock treated with DMSO, another group of cells were treated with 10 μM of 3TC simultaneously upon tet withdrawal. Fresh culture media with or without 3TC were replenished at 2-day intervals. Cells continued to be cultured in tet-free medium for 14 days. Cells and culture fluids were harvested at the indicated time points after tet removal. (A) Cells were harvested at day 8 after withdrawal of tet, the intracellular total viral RNA and encapsidated pgRNA were analyzed by Northern blotting. (B) Cytoplasmic core DNA accumulated at indicated time points were analyzed by Southern blotting (upper panel), the viral DNA and RNA in extracellular HBV virions and naked capsids at indicated time points were detected by particle gel assay using (-) strand-specific probe (middle panel) and (+) strand-specific probe (lower panel), respectively. (TIF) [file ppat.1008945.s001.tif]

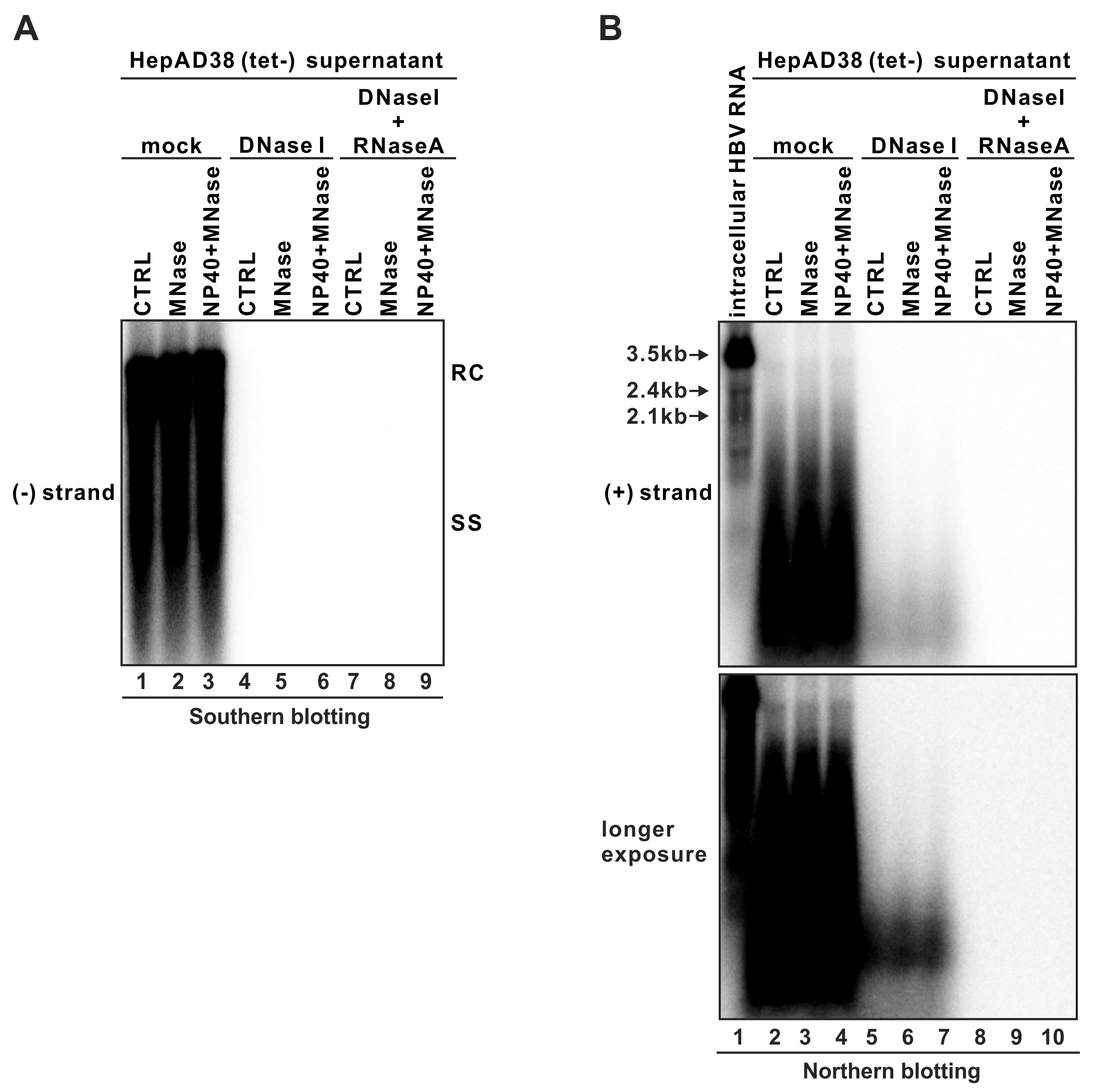

Supplement: S2 Fig — The supernatant of induced HepAD38 (tet-) were either untreated or treated with NP40 (final concentration of 0.5%) for 10 min at room temperature, followed by MNase (20 units/μl) digestion in the presence of 5 mM CaCl2 for 15 min at 37°C or remain untreated. Viral DNA/RNA were co-purified by QIAamp MinElute Virus Spin Kit and subjected to DNaseI digestion alone, DNaseI plus RNaseA double digestion, or remain untreated. After digestion, the samples were subjected to Southern blotting (A) and Northern blotting (B) with (-) strand- and (+) strand-specific HBV probes, respectively. 5 μg of total RNA from pCMVHBV-transfected HepG2 cells served as a control for intracellular HBV RNA. (TIF) [file ppat.1008945.s002.tif]

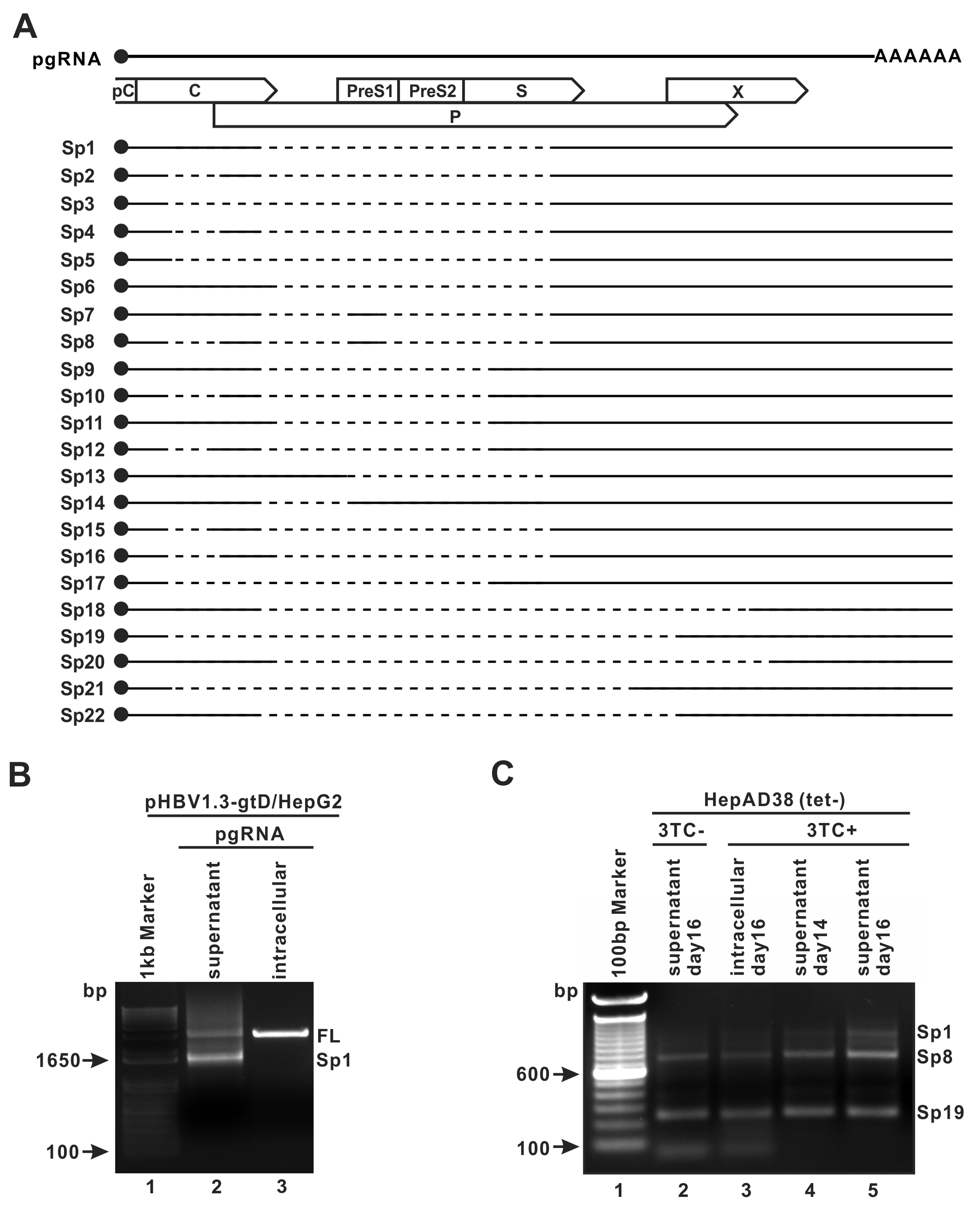

Supplement: S3 Fig — (A) Schematic illustration of the reported spliced pgRNA (Sp) variants. The full-length pgRNA and the overlapping ORFs are shown on the top, the Sp variants are aligned underneath, and introns are indicated with dotted lines. The numbering of Sp variants is according to literature [14, 22, 23]. Sp22 is a novel putative splicing variant (Fig 6C). (B) HepG2 cells were transfected with genotype D HBV plasmid pHBV1.3-gtD for 5 days. The pgRNA Sp variants of intracellular and supernatant RNA were analyzed by RT-PCR using genotype D-specific RT and pgRNA splicing primers (S1 Table). The distinct amplicon bands were cloned and sequenced, their corresponding Sp variants were indicated. FL: full-length amplicon from unspliced pgRNA. (C) HepAD38 cells were induced and treated with or without 3TC for the indicated time durations, the intracellular and extracellular Sp variants were determined by RT-PCR and sequencing. (TIF) [file ppat.1008945.s003.tif]

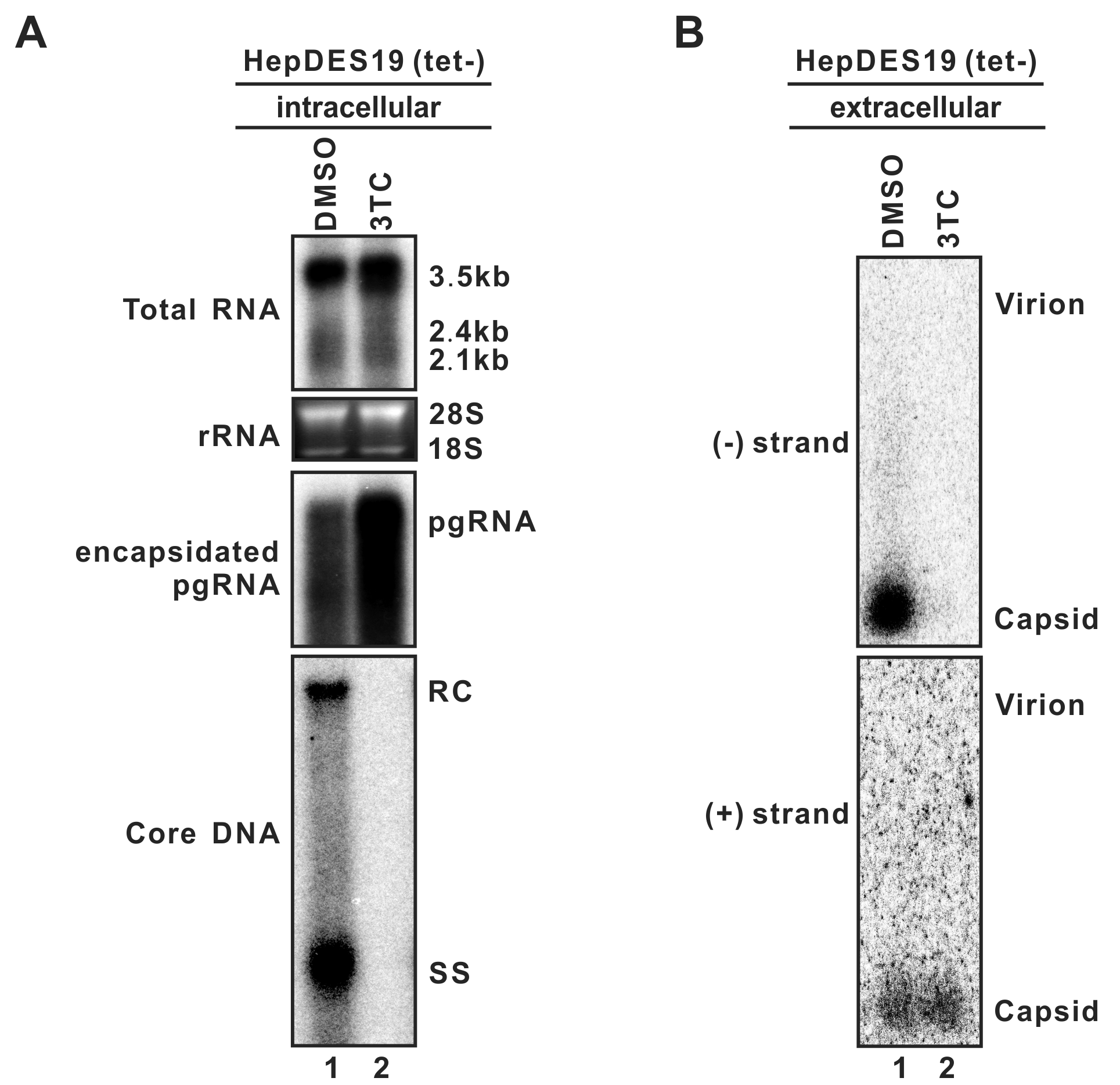

Supplement: S4 Fig — HepDES19 Cells were cultured in the presence of tet (1 μg/ml) until cells became confluent, and tet was then removed from culture medium to induce pgRNA transcription. One group of cells were treated with DMSO and the other group of cells were treated with 10 μM of 3TC simultaneously with tet withdrawal. Cells were harvested at day 14 after withdrawal of tet. (A) Intracellular total viral RNA and encapsidated pgRNA, core DNA were analyzed by Northern and Southern blot, respectively. (B) Extracellular HBV DNA- and RNA-containing capsids were analyzed by particle gel and hybridization. (TIF) [file ppat.1008945.s004.tif]

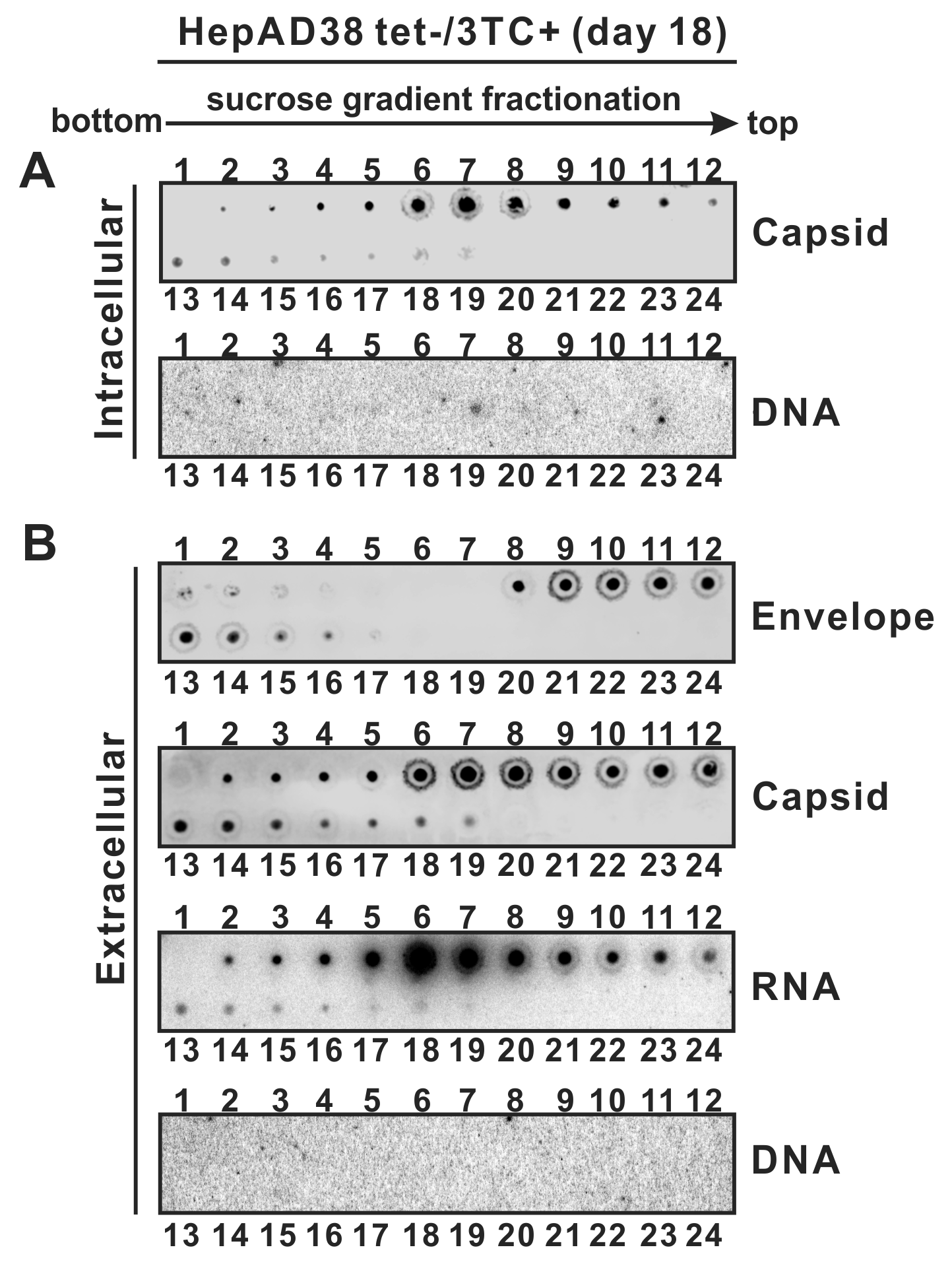

Supplement: S5 Fig — (A) Distribution of intracellular HBV particle-associated core protein and DNA in sucrose gradient. HBV particles prepared from the lysate of 3TC-treated HepAD38 cell were overlaid on a 5% to 55% (wt/wt) sucrose gradient for ultracentrifugation separation. Fractions were collected from bottom to top and spotted onto nitrocellulose membrane, followed by immunoblotting of HBV capsids with anti-HBcAg antibodies. The nitrocellulose membranes were then denatured for HBV DNA hybridization. (B) Distribution of extracellular HBV particle-associated antigens and DNA/RNA in sucrose gradient. HBV particles were concentrated from 3TC-treated HepAD38 cell culture supernatant by PEG-8000, and were layered over a 5% to 55% (wt/wt) sucrose gradient for ultracentrifugation separation. Fractions were collected from bottom to top and spotted onto a nitrocellulose membrane. HBV envelope and core proteins were detected by immunoblotting with anti-HBsAg and anti-HBcAg antibodies. Virus particle-associated DNA/RNA were detected by hybridization as described in Materials and Methods. Fractions 9–12 were collected as 3TC-arrested RNA virions. (TIF) [file ppat.1008945.s005.tif]

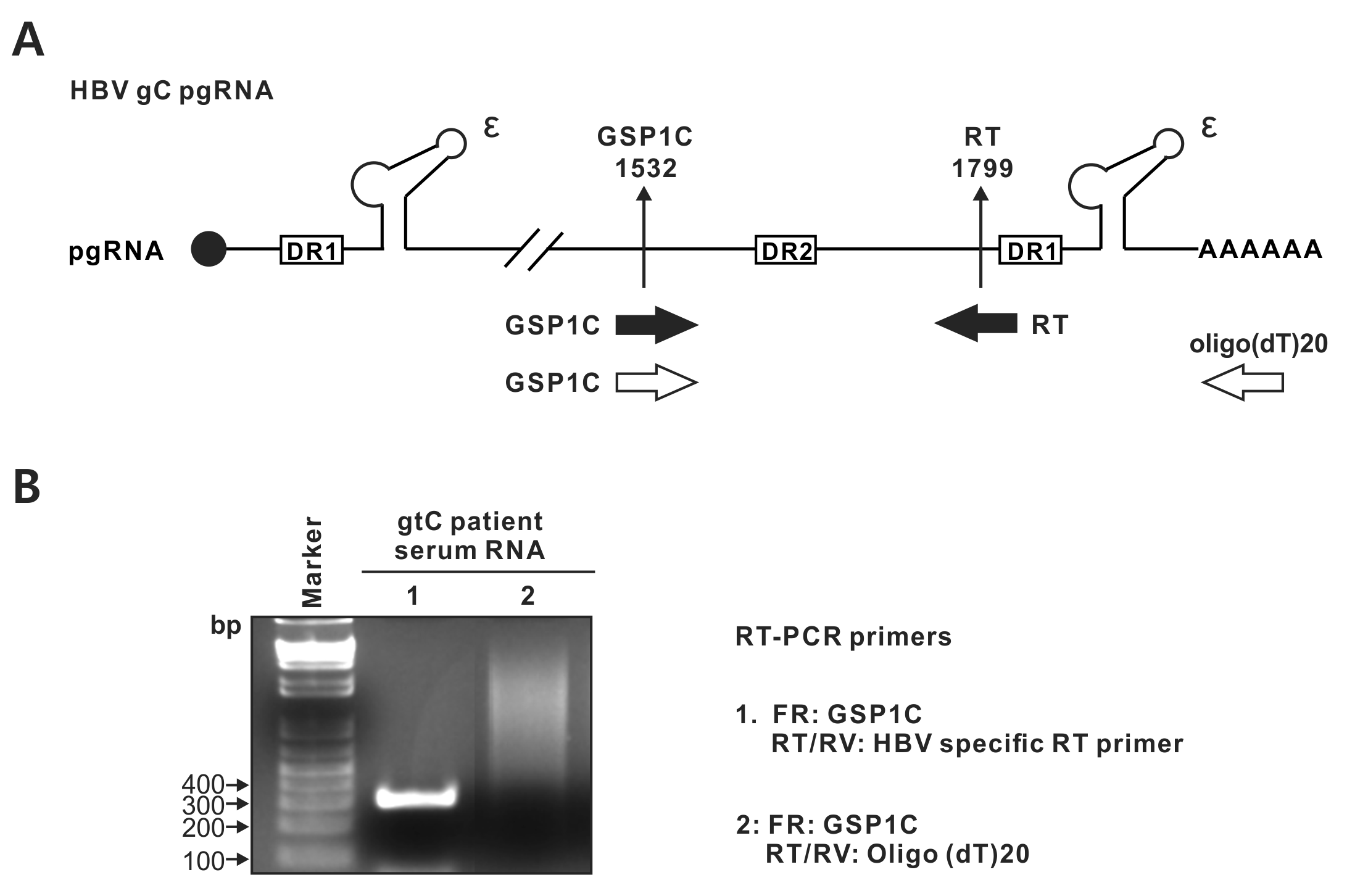

Supplement: S6 Fig — Serum RNA purified from the genotype C HBV-infected CHB patient were subjected to RT-PCR with the indicated primers (A). The PCR products were resolved by electrophoresis and stained by ethidium bromide (B). (TIF) [file ppat.1008945.s006.tif]

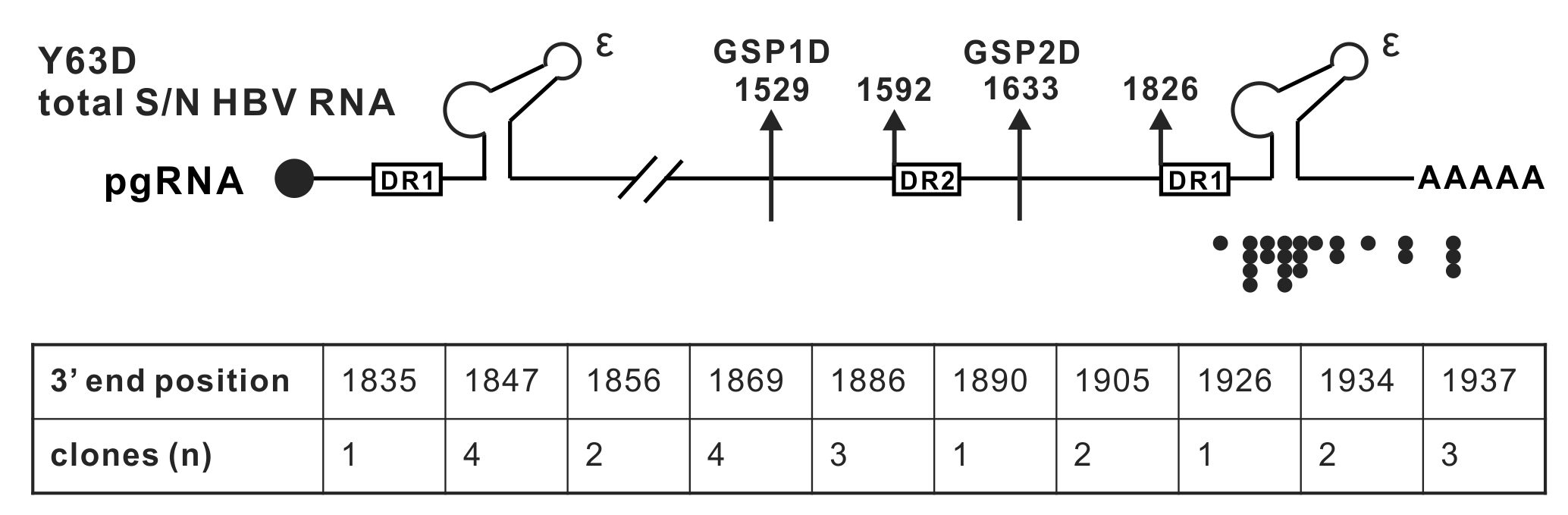

Supplement: S7 Fig — HepG2 cells in 6-well plate were transfected with the pCMVHBV-Y63D (Y63D) for 5 days. The extracellular total HBV RNA was analyzed by 3’ RACE and clone sequencing as described in Materials and Methods. The positions of DR1, DR2, and gtD HBV gene specific primers for nested PCR (GSPD1 and GSPD2) are indicated. The nucleotide positions and numbers of mapped 3’ termini of HBV RNA are indicated with solid dots underneath the illustrated full-length pgRNA and listed in the table underneath. The RNA molecules with 3’ end position mapped at nt1937 are polyadenylated. (TIF) [file ppat.1008945.s007.tif]

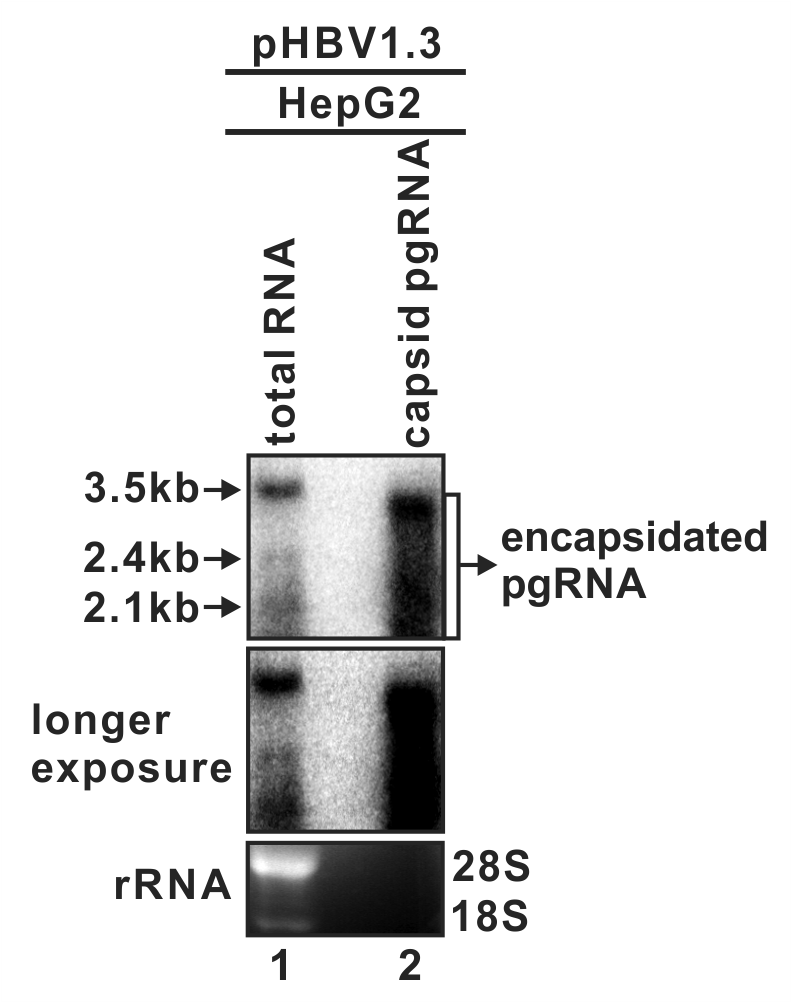

Supplement: S8 Fig — HepG2 cells in 12-well plate were transfected with 1.6 μg of pHBV1.3. Cells were harvested at day 5 post-transfection and the intracellular viral total RNA and encapsidated pgRNA (capsid pgRNA) were extracted and analyzed by Northern blotting as described in Materials and Methods. 5 μg of total RNA (lane 1) and the capsid pgRNA extracted from one well of a 12-well plate (lane 2) were hybridized with a plus (+) strand-specific full length HBV riboprobe. Ribosomal RNA (28S and 18S rRNA) served as total RNA loading control (lane 1), and the loss of rRNA indicated a complete digestion of unencapsidated RNA by MNase (lane 2). HBV 3.5kb, 2.4kb, 2.1kb RNAs, and capsid pgRNA are labeled. A longer exposure of the blot is included. (TIF) [file ppat.1008945.s008.tif]

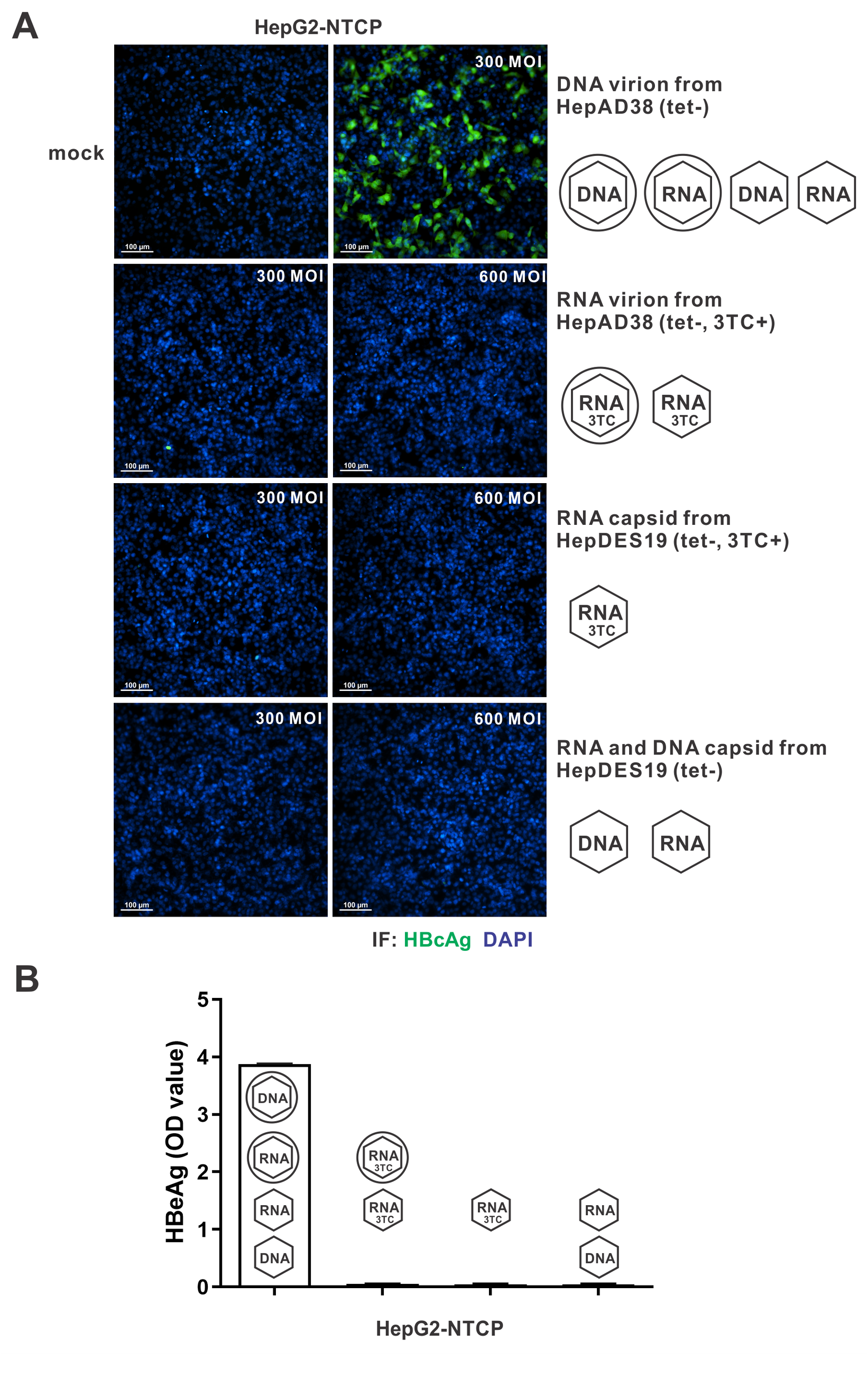

Supplement: S9 Fig — (A) HepG2-NTCP cells were left uninfected (mock) or infected with HBV particles collected from the supernatant of induced HepAD38 cells or HepDES19 cells with or without 3TC treatment at indicated MOI. The composition of viral particles in each inoculum was indicted by illustrations. At day 10 post-inoculation, the expression of intracellular HBcAg was analyzed by immunofluorescence. (B) HBeAg in the supernatant was detected by ELISA. (TIF) [file ppat.1008945.s009.tif]

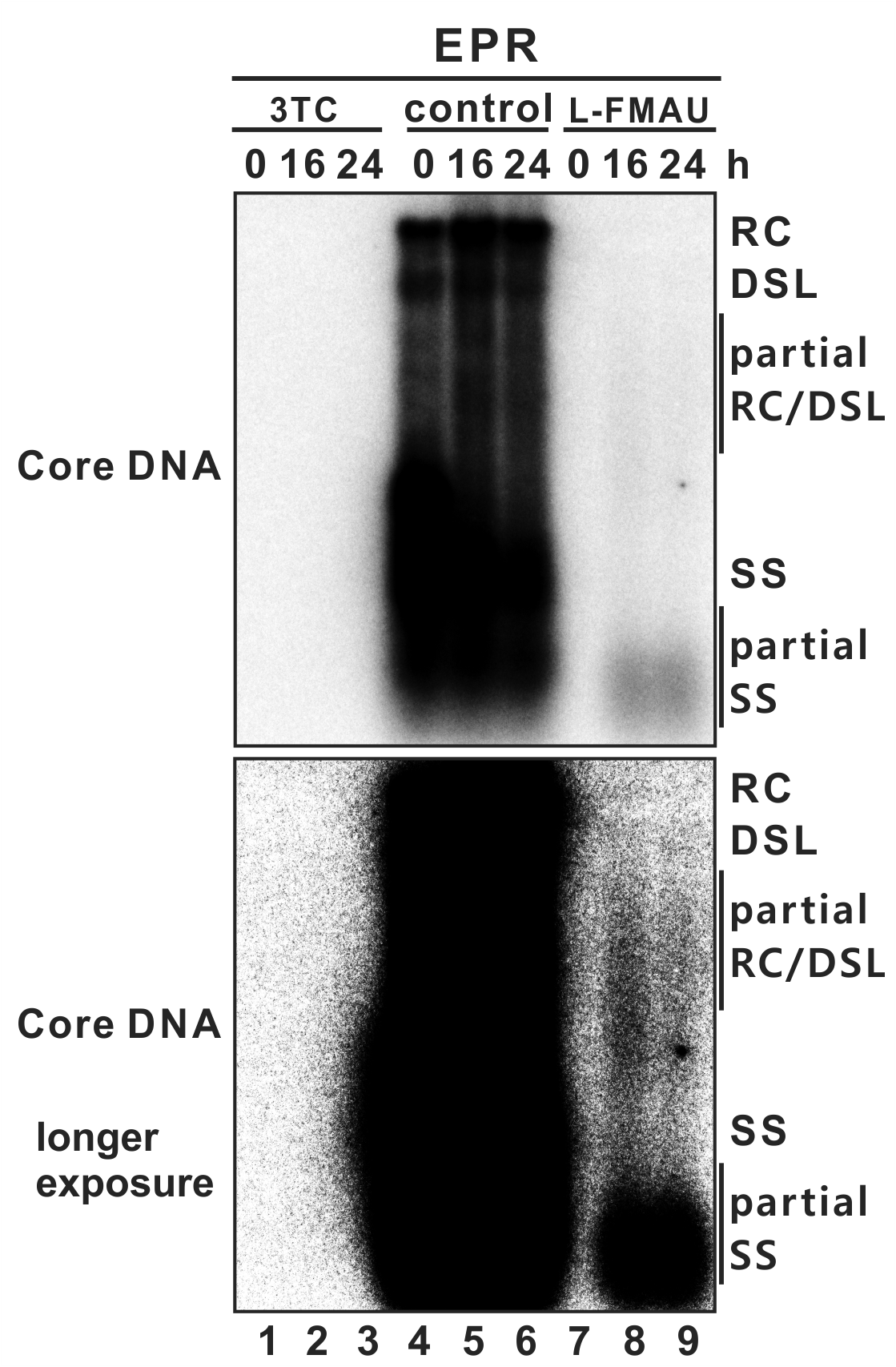

Supplement: S10 Fig — Cytoplasmic HBV nucleocapsids were purified from untreated and 3TC- or L-FMAU-treated HepAD38 cells, and subjected to EPR assay as described in Materials and Methods. The reaction was terminated at indicated time points and HBV core DNA were analyzed by Southern blot hybridization. (TIF) [file ppat.1008945.s010.tif]

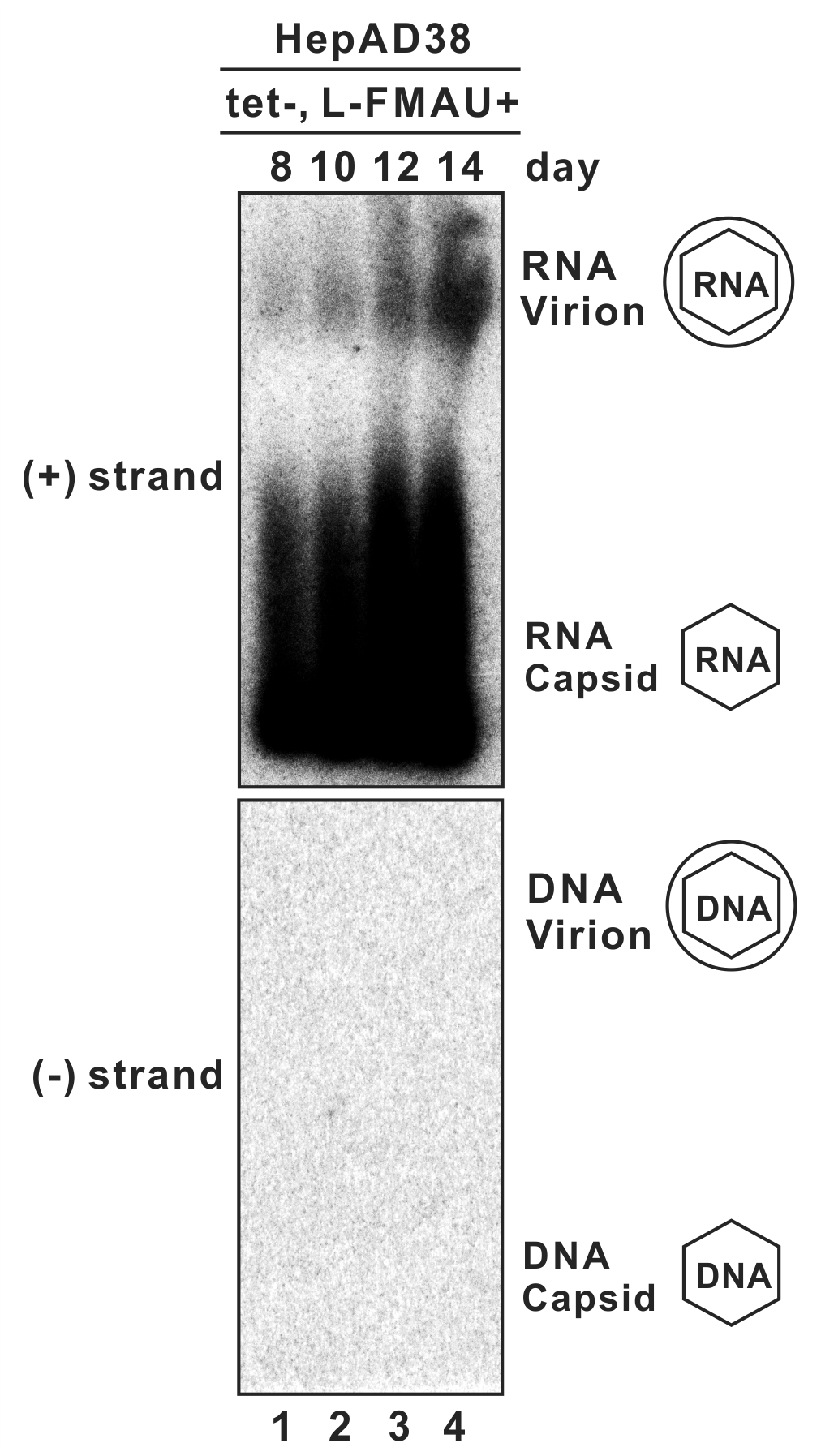

Supplement: S11 Fig — Upon the withdrawal of tet, HepAD38 cells were treated with 40 μM of L-FMAU simultaneously. L-FMAU treatment was replenished at 2-day intervals for 16 days. Cell culture fluids were harvested at the indicated time points after tet removal. The extracellular accumulation of HBV RNA virion and capsid particles were analyzed by particle gel assay and hybridization with (+) strand-specific HBV probe. (TIF) [file ppat.1008945.s011.tif]
