# Supplementary material for: Biogenesis and molecular characteristics of serum hepatitis B virus RNA
Source: PLoS Pathog. 2020 Oct 20;16(10):e1008945. doi: 10.1371/journal.ppat.1008945 (PMC7575114; doi:10.1371/journal.ppat.1008945)
Supplement: S1 Table — (DOCX) [file ppat.1008945.s012.docx]

**S1 Table. Primers used in the study.**

| HBV Genotype | Primers | | |
| --- | --- | --- | --- |
|  | Name | Forward (5’→3’) | Reverse (5’→3’) |
| C | Precore | nt 1799-1819  GTCTGTTCACCAGCACCATGC | nt 2002-1980  CGGTGTCGAGGAGATCTCGAATA |
|  | Splicing pgRNA | nt 1827-1849  TCACCTCTGCCTAATCATCTCAT | nt 1471-1456  CCCCGAGACGGGTCGT |
|  | RT | n/a | nt 1819-1799  GCATGGTGCTGGTGAACAGAC |
|  | GSP1C | nt1532-1551  CGACTCTCTTTACGCGGTCTCCC | n/a |
|  | GSP2C | nt1638-1654  CGATCTTGCCCAAGGTCTTA | n/a |
| D | Splicing pgRNA | nt1824-1848  TTTTCACCTCTGCCTAATCATCTCT | nt 1491-1473  GACGAGAGAGTCCCAAGCG |
|  | RT | n/a | nt 1824-1806  AAGTTGCATGGTGCTGGTG |
|  | GSP1D | nt1529-1548  CGACACCTCTCTTTACGCGGACT | n/a |
|  | GSP2D | nt1633-1652  CGACCGAATGTTGCCCAAGGTCT | n/a |
