# Supplementary material for: Correction: Biogenesis and molecular characteristics of serum hepatitis B virus RNA
Source: PLoS Pathog. 2026 Aug 20;22(8):e1014527. doi: 10.1371/journal.ppat.1014527 (PMC13492790; doi:10.1371/journal.ppat.1014527)
Supplement: Supplementary file 1 [file ppat.1014527.s004.zip › S1 File/S1 File/Figure 2A_gel image and blots.pdf]

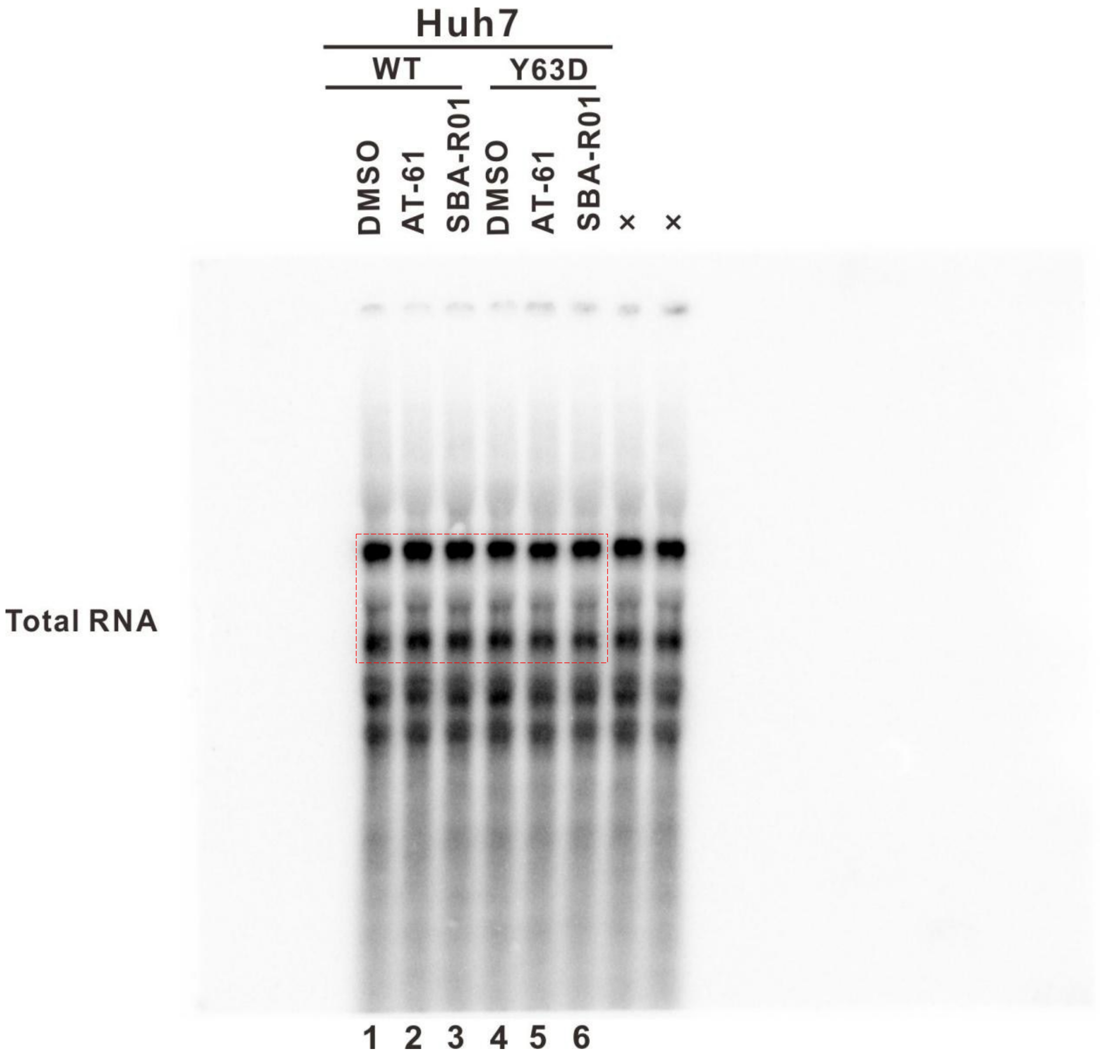
Huh7
WT
Y63D
DMSO
AT-61
SBA-R01
DMSO
AT-61
SBA-R01
×
×
Total RNA
1 2 3 4 5 6

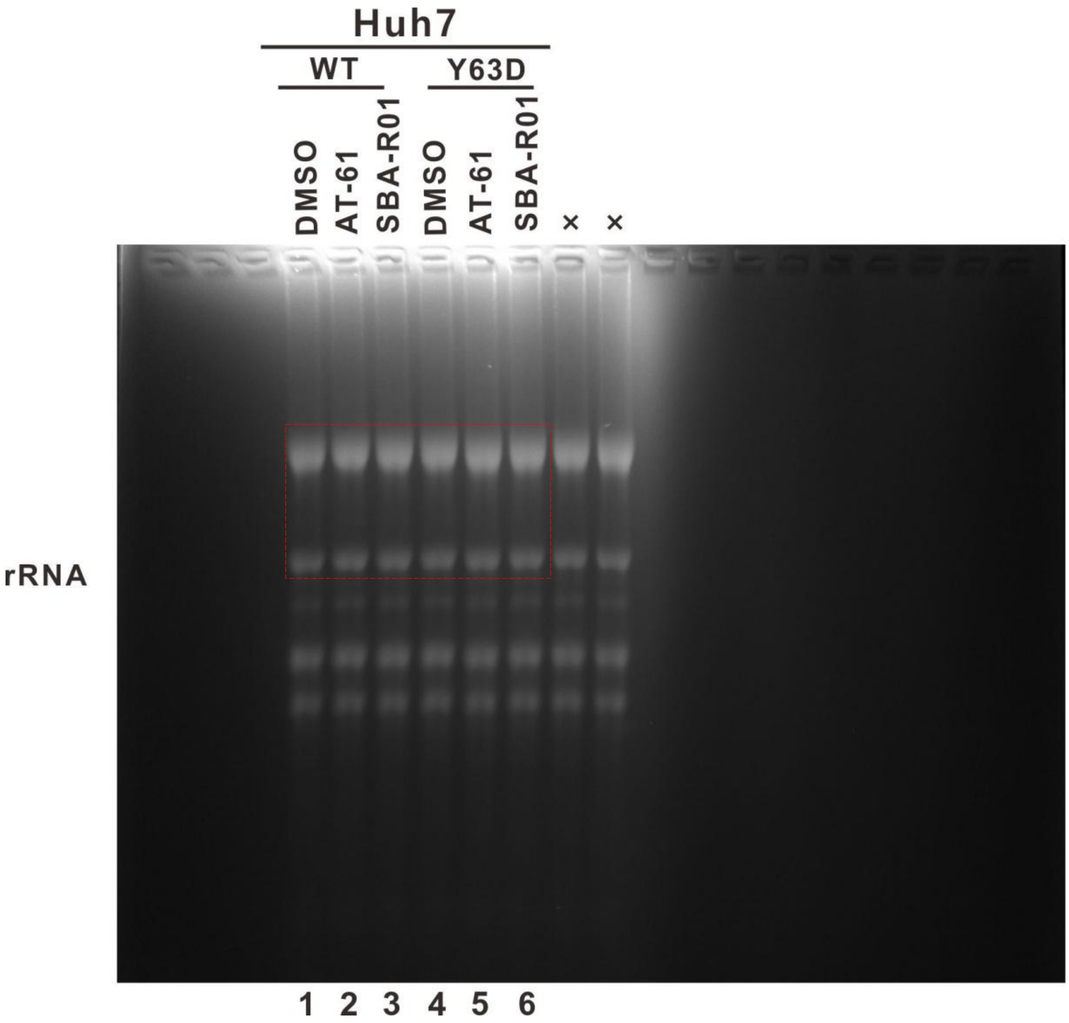
Huh7
WT
Y63D
DMSO
AT-61
SBA-R01
DMSO
AT-61
SBA-R01
×
×
rRNA
1
2
3
4
5
6

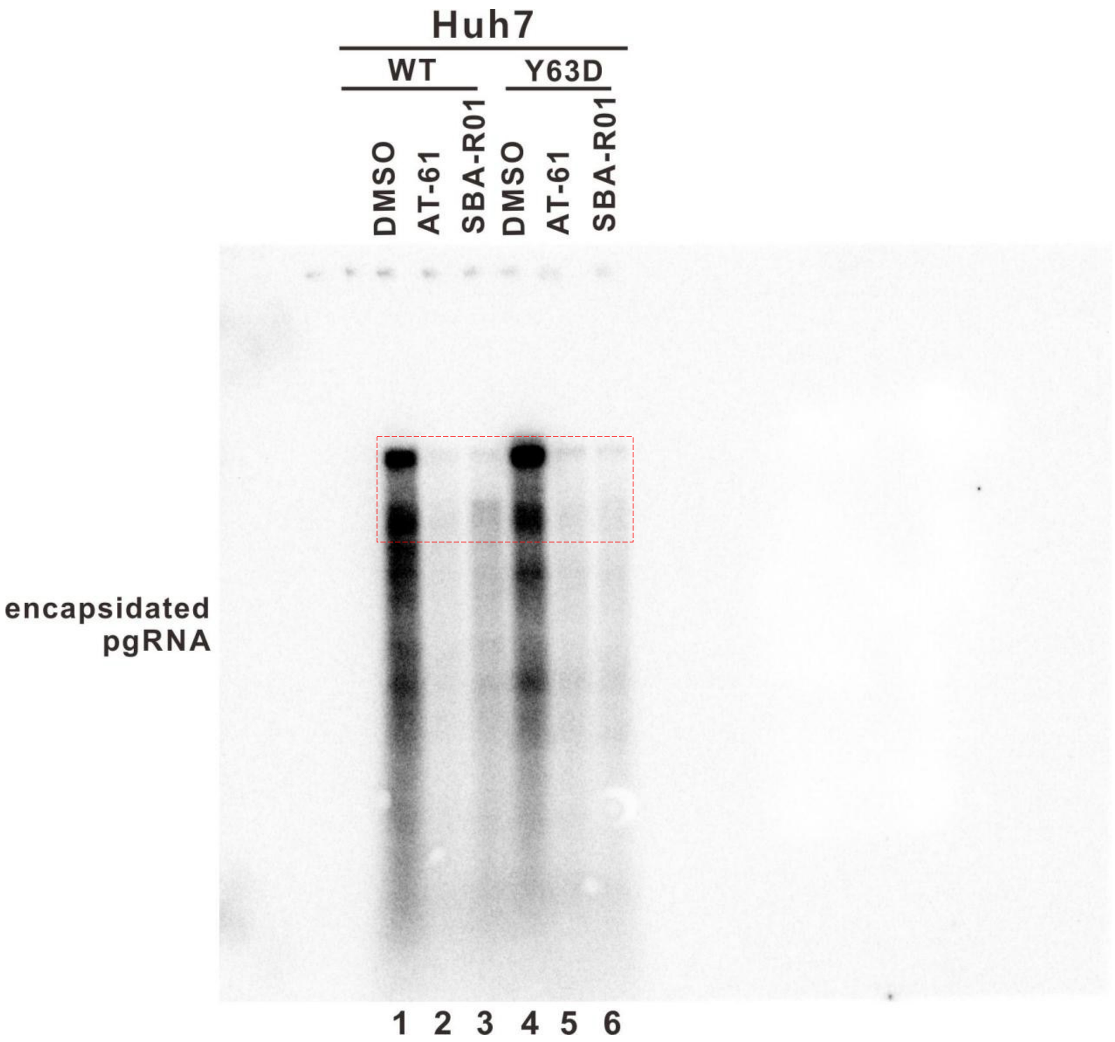
Huh7
WT
Y63D
DMSO
AT-61
SBA-R01
DMSO
AT-61
SBA-R01
encapsidated pgRNA
1
2
3
4
5
6

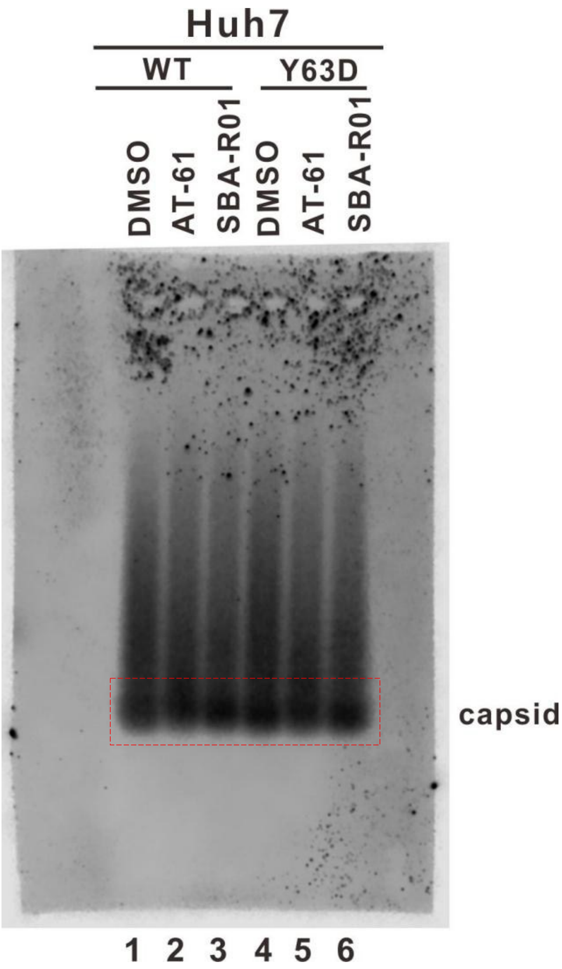
Huh7
WT
Y63D
DMSO
AT-61
SBA-R01
DMSO
AT-61
SBA-R01
capsid
1 2 3 4 5 6

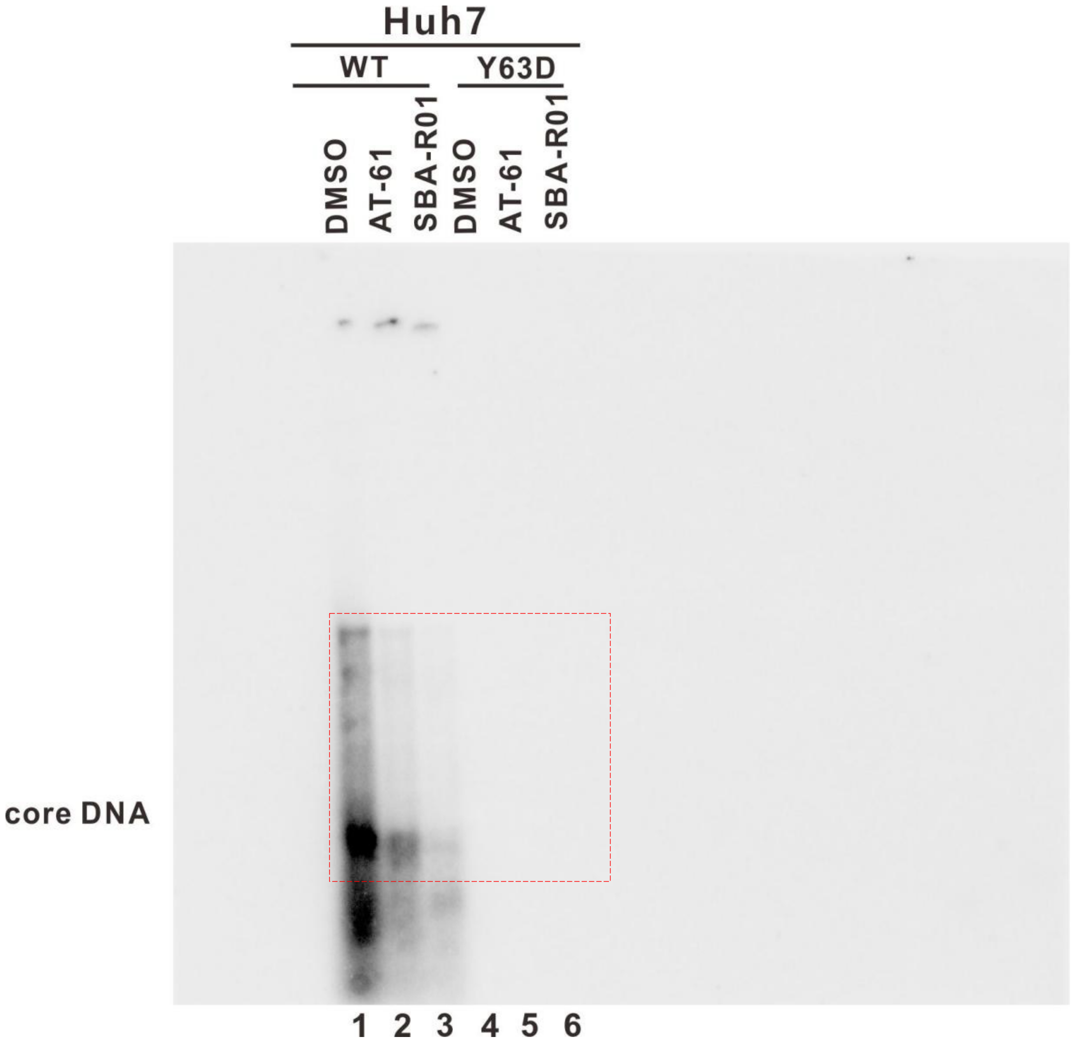
Huh7
WT
Y63D
DMSO
AT-61
SBA-R01
DMSO
AT-61
SBA-R01
core DNA
1
2
3
4
5
6

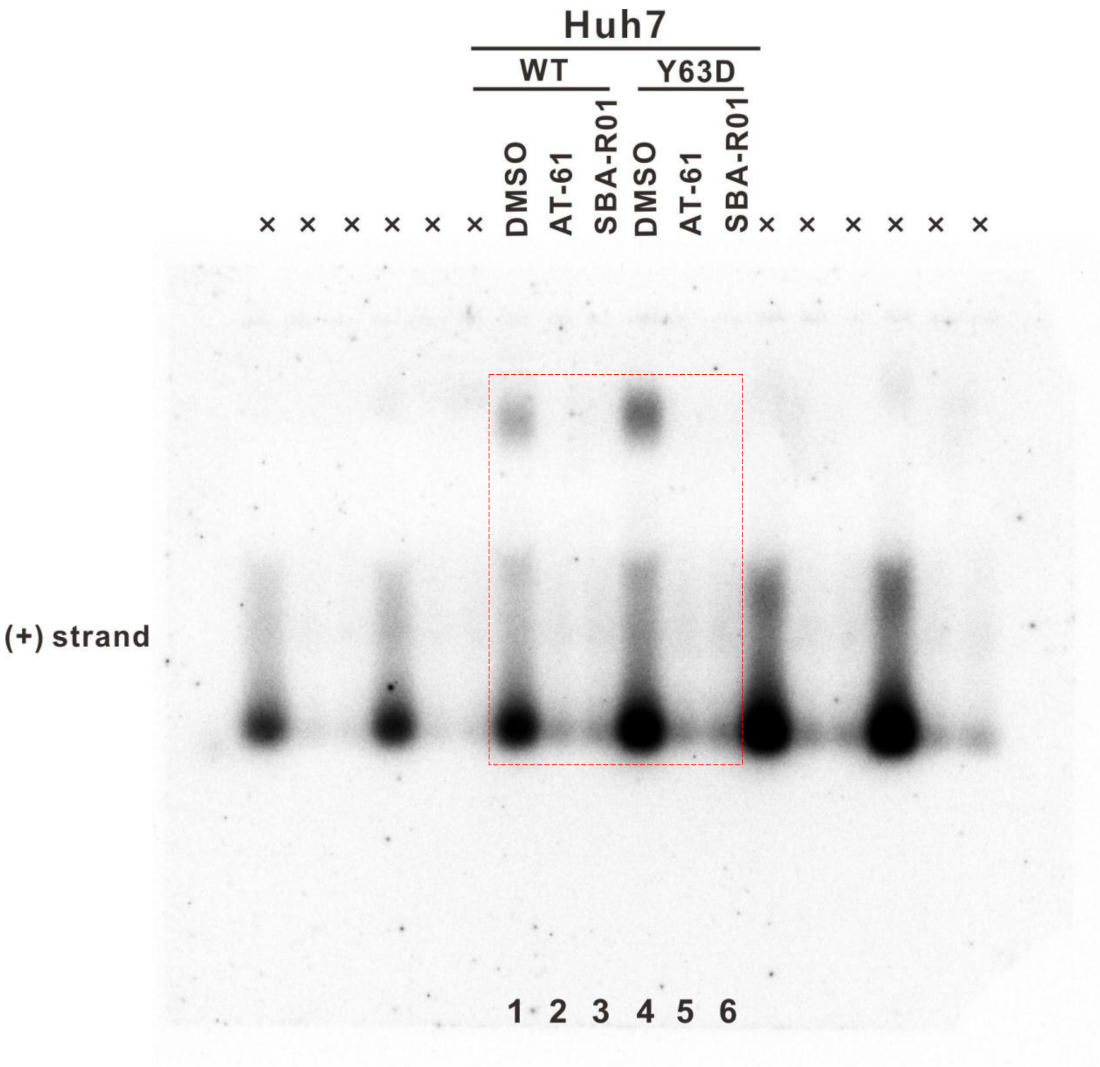
Huh7
WT
Y63D
DMSO
AT-61
SBA-R01
DMSO
AT-61
SBA-R01
(+) strand
1 2 3 4 5 6
