# Supplementary material for: Correction: Biogenesis and molecular characteristics of serum hepatitis B virus RNA
Source: PLoS Pathog. 2026 Aug 20;22(8):e1014527. doi: 10.1371/journal.ppat.1014527 (PMC13492790; doi:10.1371/journal.ppat.1014527)
Supplement: Supplementary file 1 [file ppat.1014527.s004.zip › S1 File/S1 File/Figure 3A_gel image and blots.pdf]

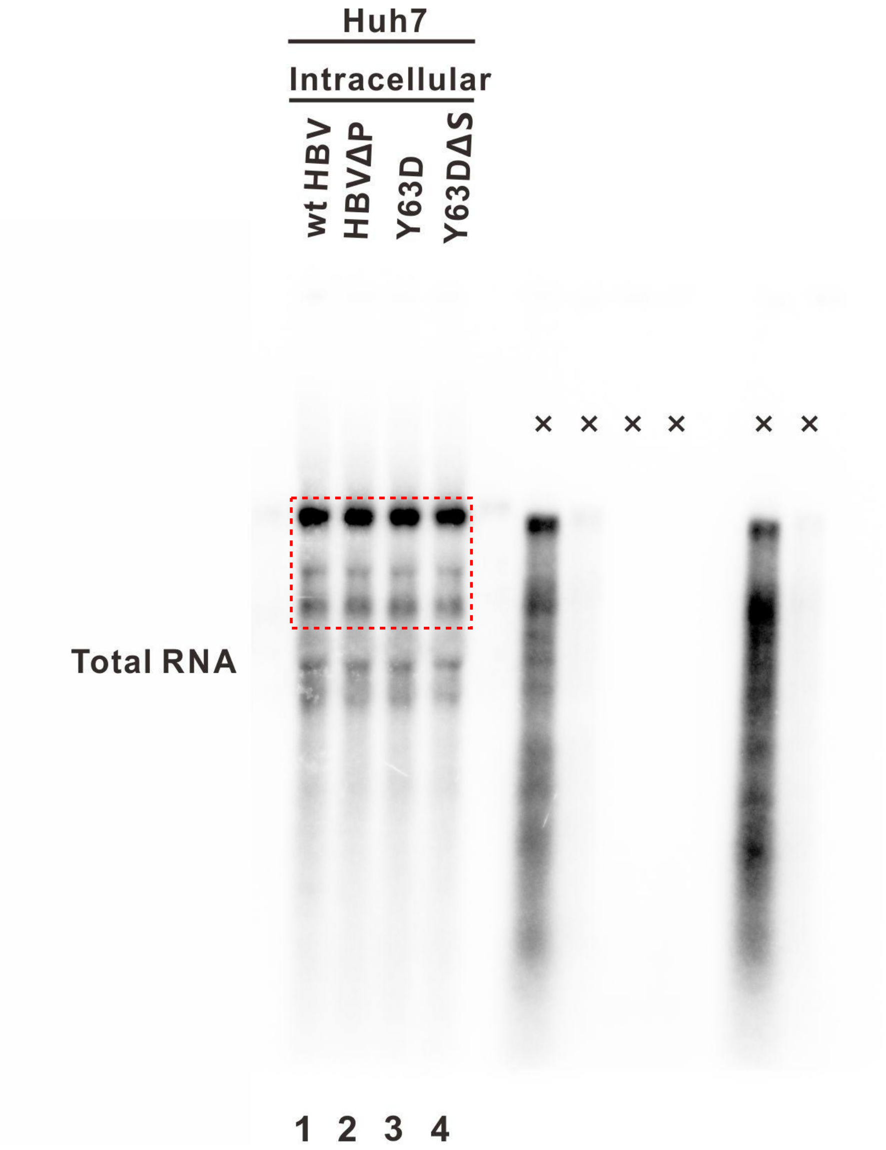
Huh7
Intracellular
wt HBV
HBVΔP
Y63D
Y63DΔS
Total RNA
× × × ×
× ×
1
2
3
4

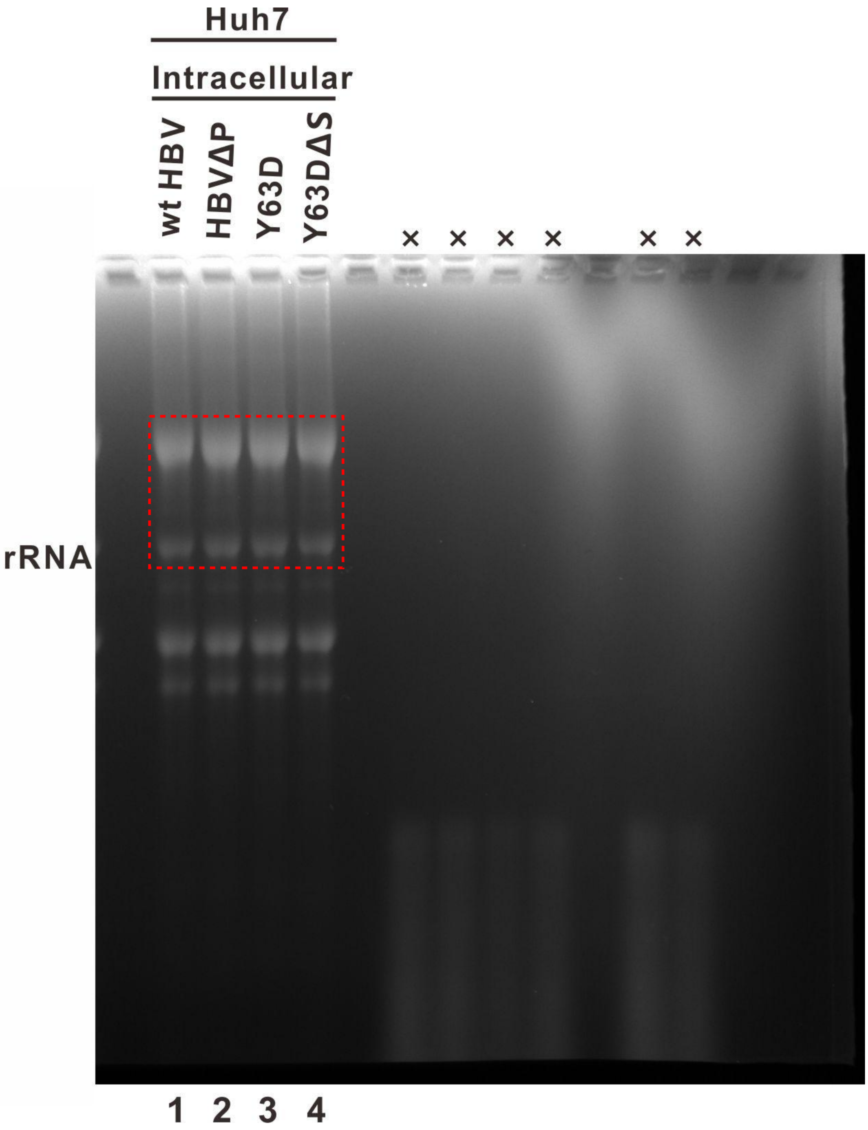
Huh7
Intracellular
wt HBV
HBVΔP
Y63D
Y63DΔS
rRNA
× × × × × ×
1 2 3 4

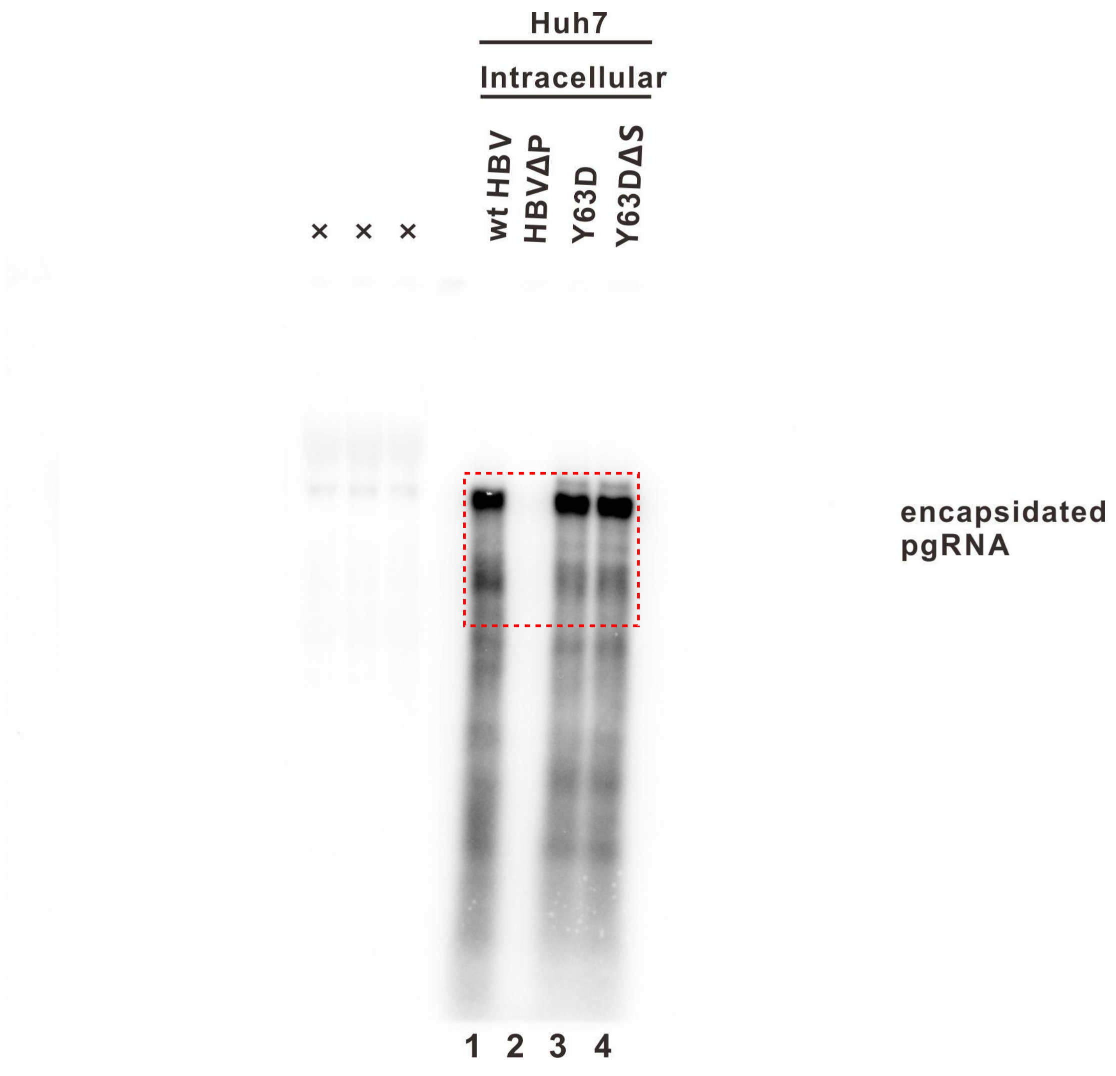

Huh7
Intracellular
×
×
×
wt HBV
HBVΔP
Y63D
Y63DΔS
encapsidated pgRNA
1
2
3
4

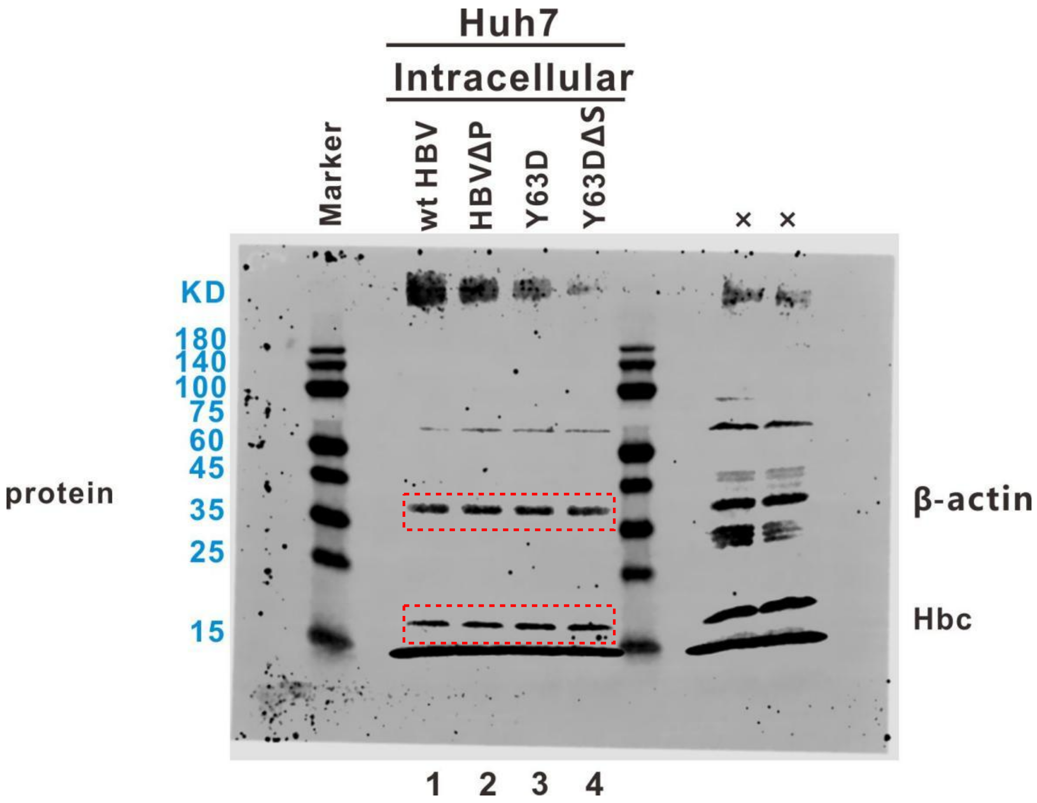

Huh7
Intracellular
Marker
wt HBV
HBVΔP
Y63D
Y63DΔS
×
×
KD
180
140
100
75
60
45
35
25
15
protein
β-actin
Hbc
1
2
3
4

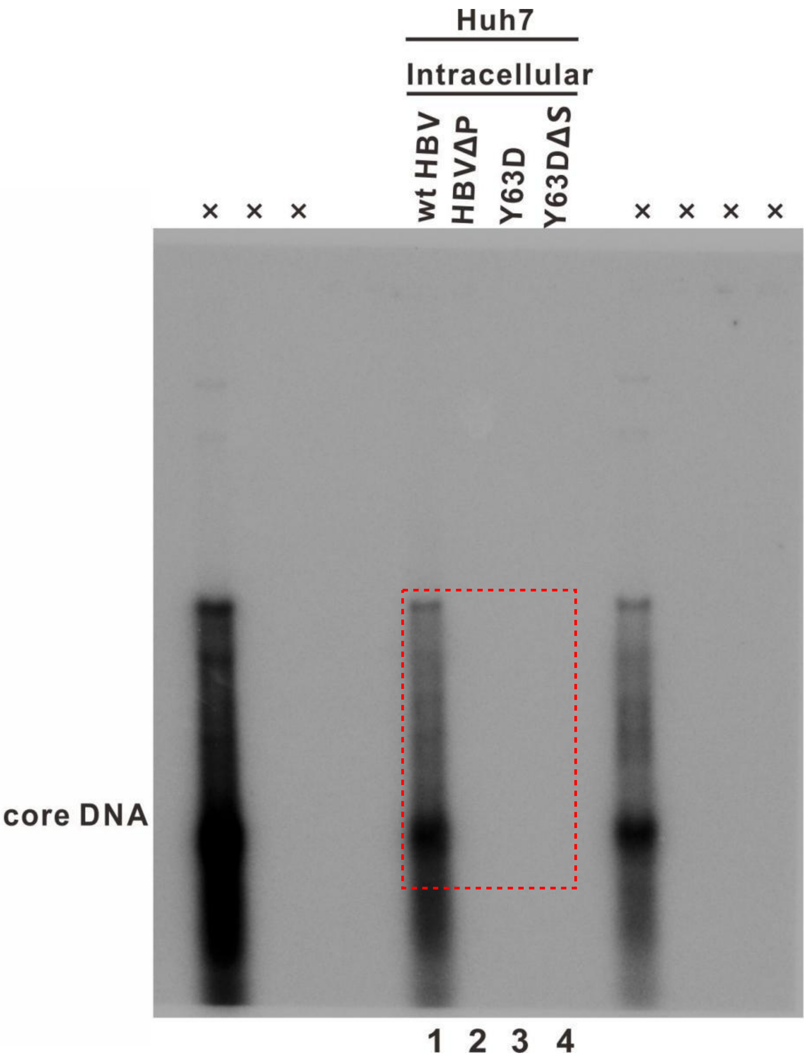
Huh7
Intracellular
wt HBV
HBVΔP
Y63D
Y63DΔS
× × ×
× × × ×
core DNA
1 2 3 4
