# Supplementary material for: Correction: Biogenesis and molecular characteristics of serum hepatitis B virus RNA
Source: PLoS Pathog. 2026 Aug 20;22(8):e1014527. doi: 10.1371/journal.ppat.1014527 (PMC13492790; doi:10.1371/journal.ppat.1014527)
Supplement: Supplementary file 1 [file ppat.1014527.s004.zip › S1 File/S1 File/Figure 5B_blots.pdf]

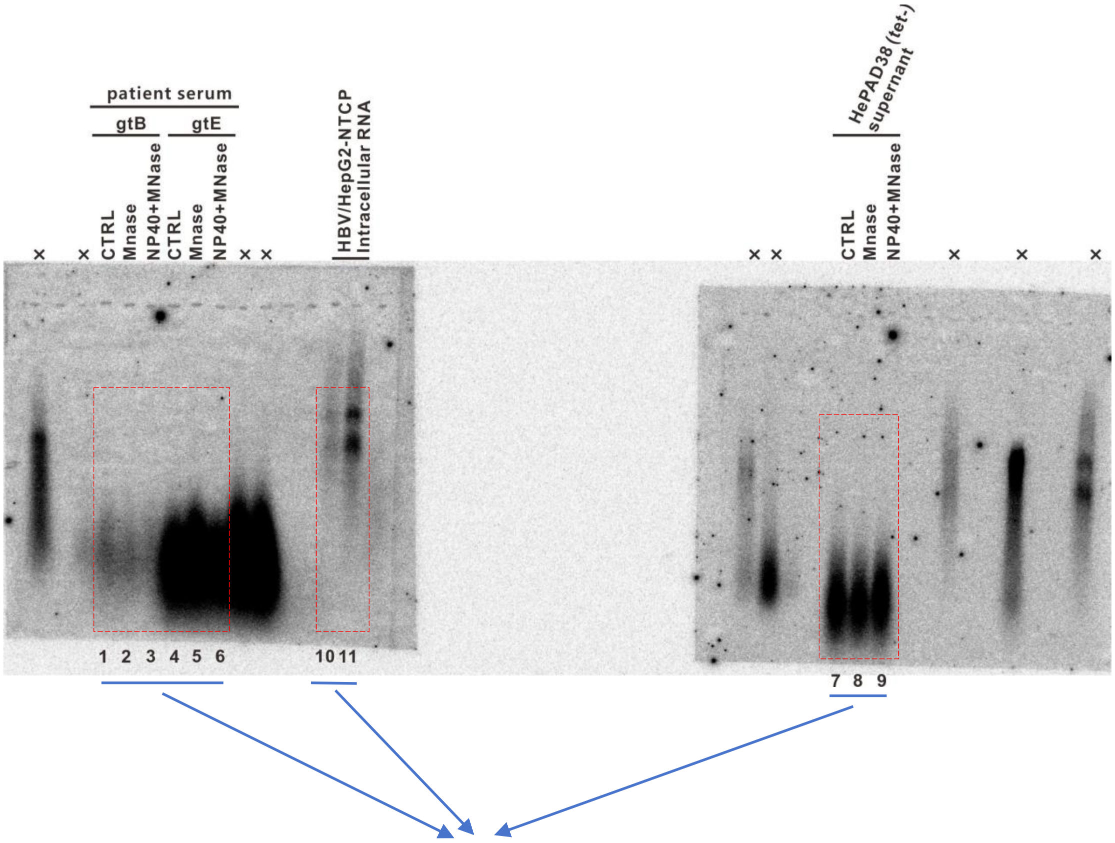


Lane# in the assembled Fig.5B
