# Supplementary material for: Correction: Biogenesis and molecular characteristics of serum hepatitis B virus RNA
Source: PLoS Pathog. 2026 Aug 20;22(8):e1014527. doi: 10.1371/journal.ppat.1014527 (PMC13492790; doi:10.1371/journal.ppat.1014527)

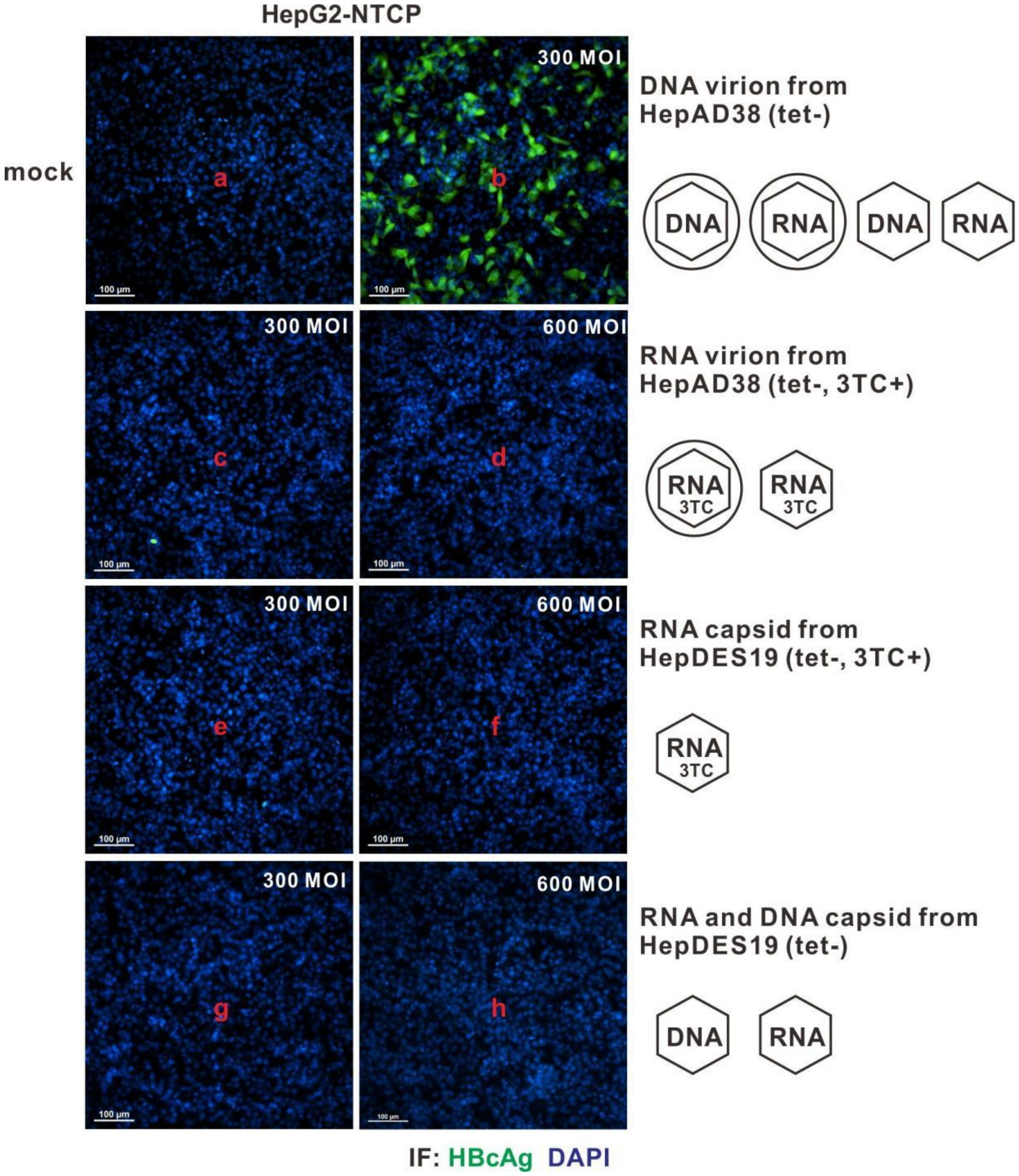
HepG2-NTCP
mock
a
b
300 MOI
DNA virion from
HepAD38 (tet-)
DNA
RNA
DNA
RNA
c
d
300 MOI
600 MOI
RNA virion from
HepAD38 (tet-, 3TC+)
RNA
3TC
RNA
3TC
e
f
300 MOI
600 MOI
RNA capsid from
HepDES19 (tet-, 3TC+)
RNA
3TC
g
h
300 MOI
600 MOI
RNA and DNA capsid from
HepDES19 (tet-)
DNA
RNA
100 μm
IF: HBcAg DAPI

a

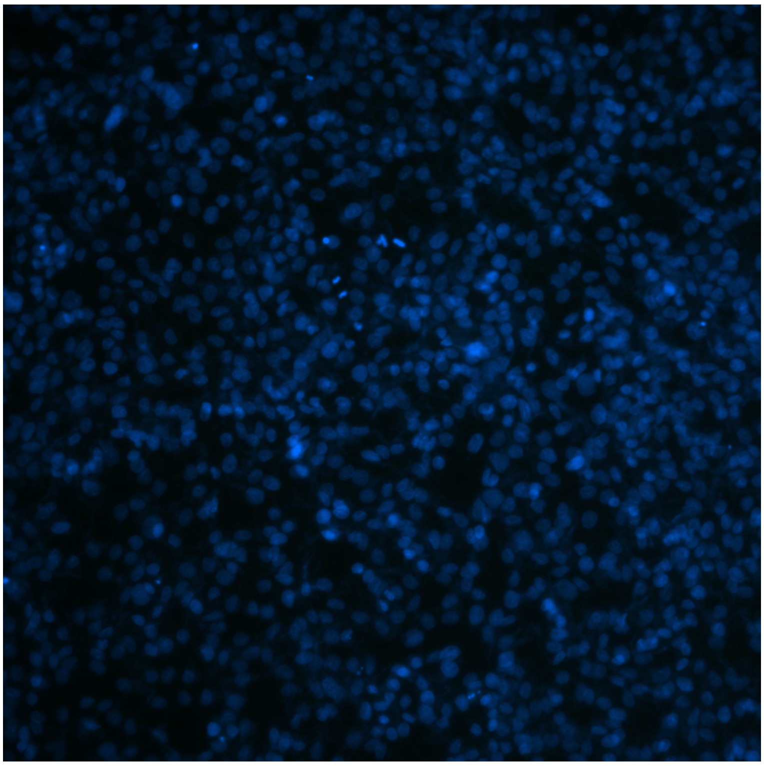

b

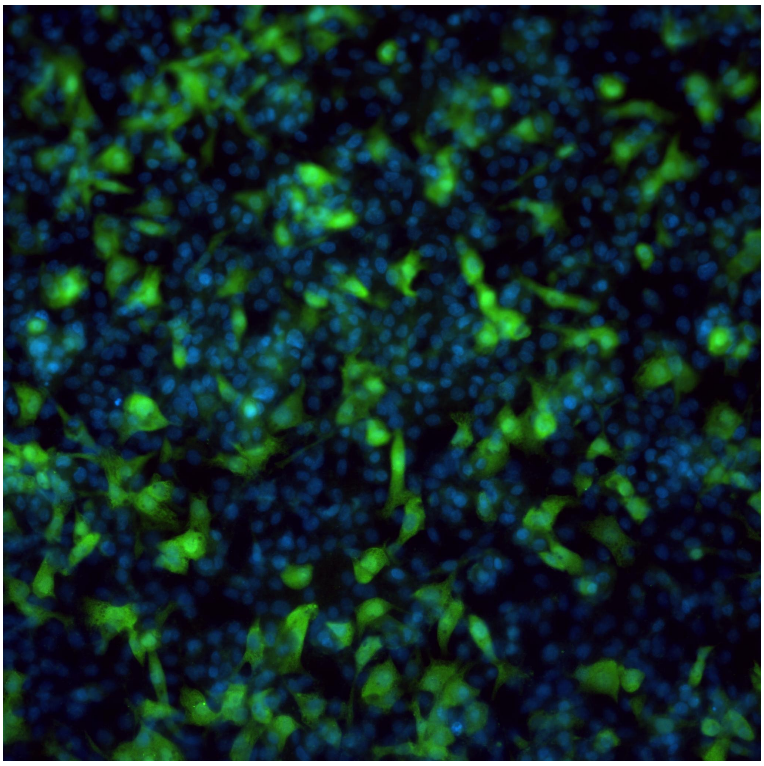

c

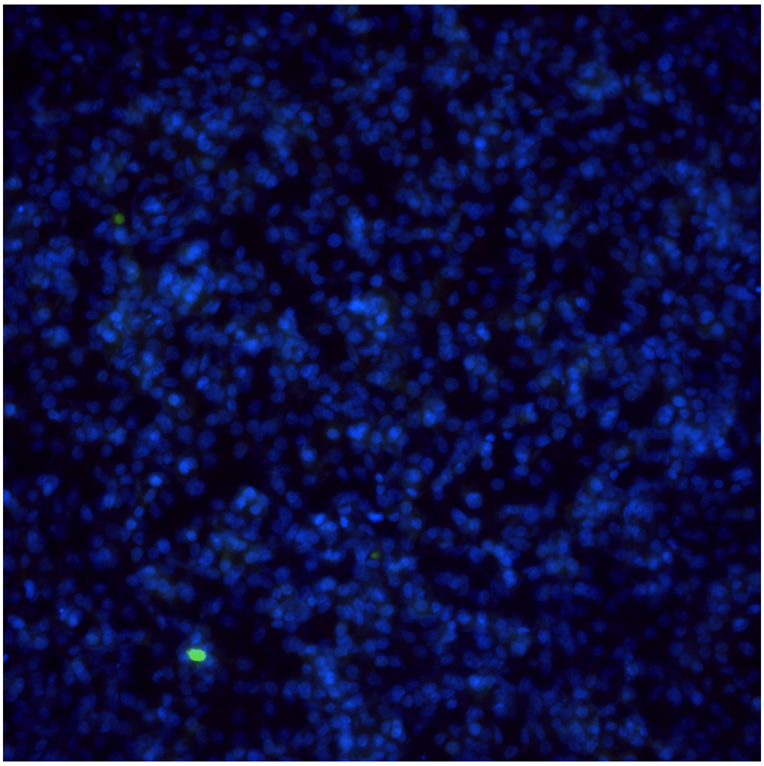

d

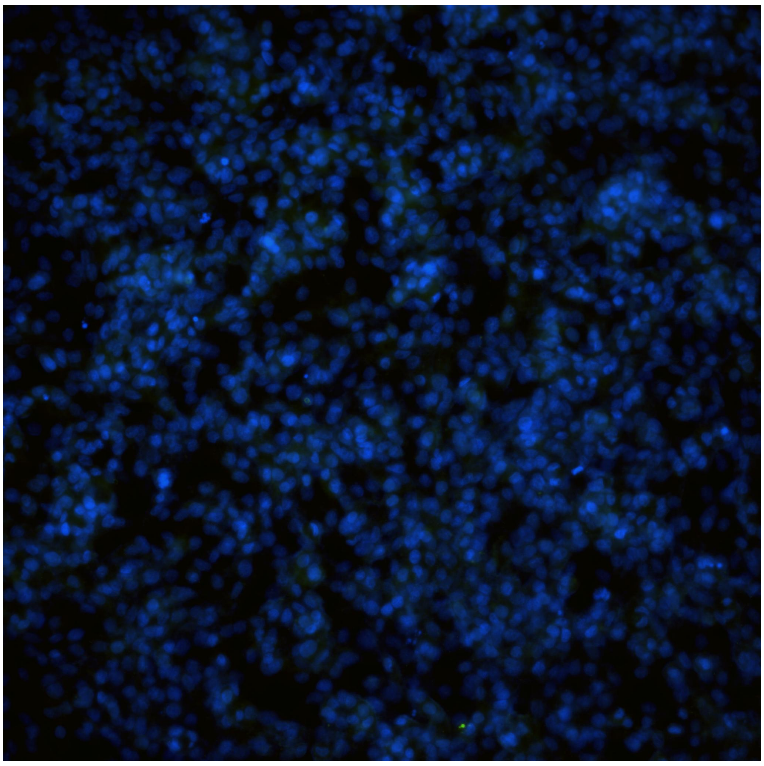

e

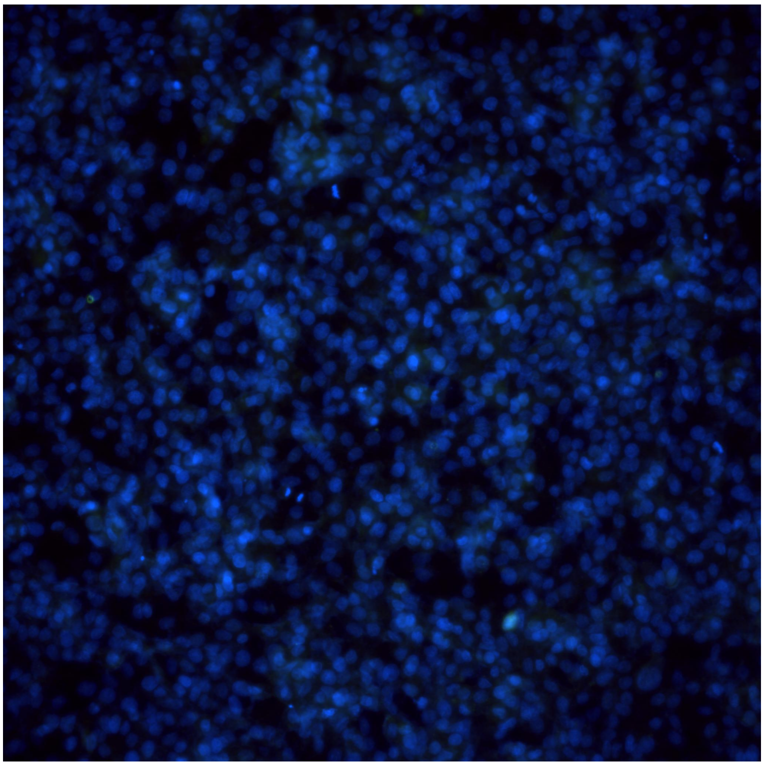

f

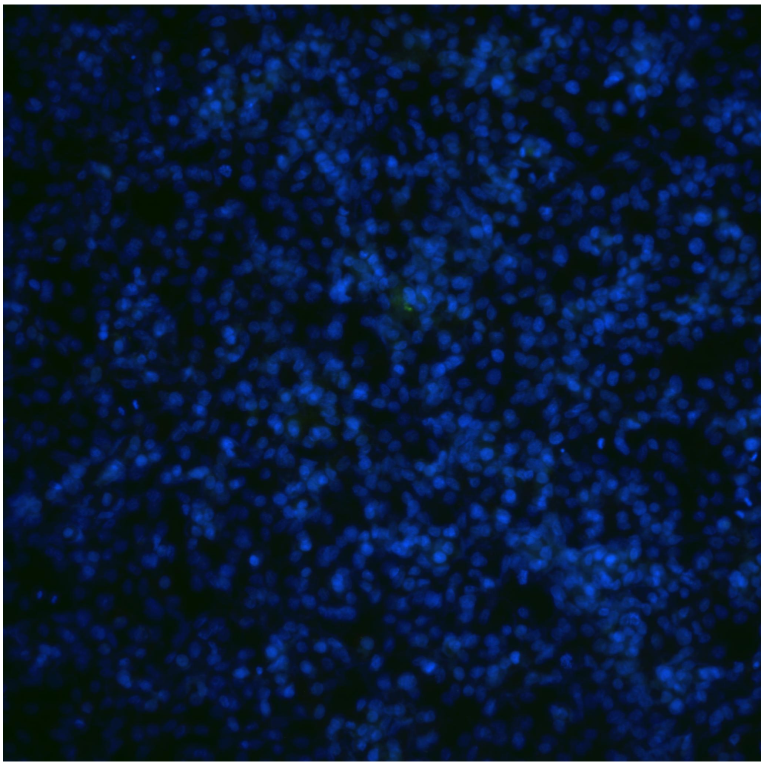

g

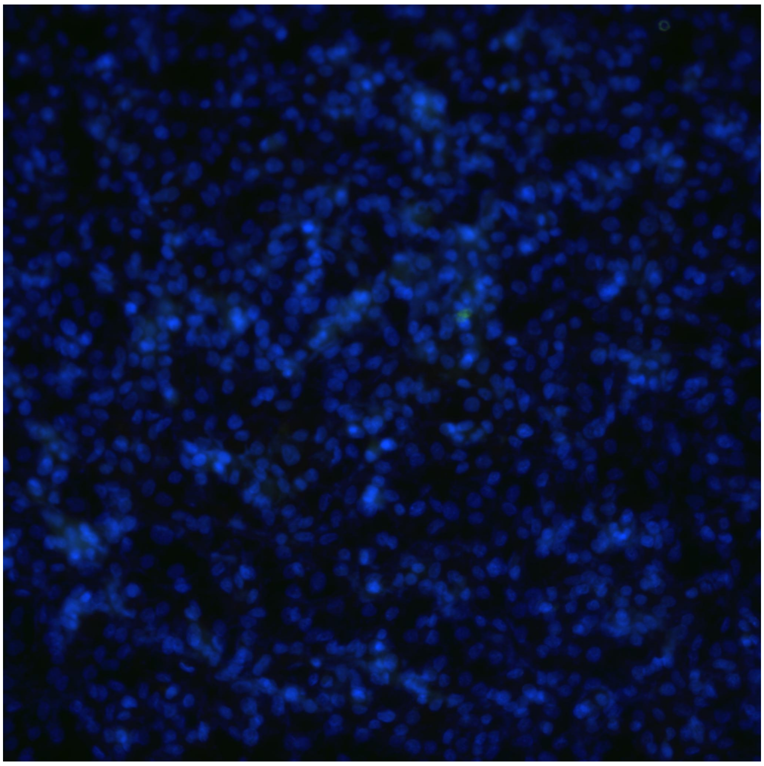

h

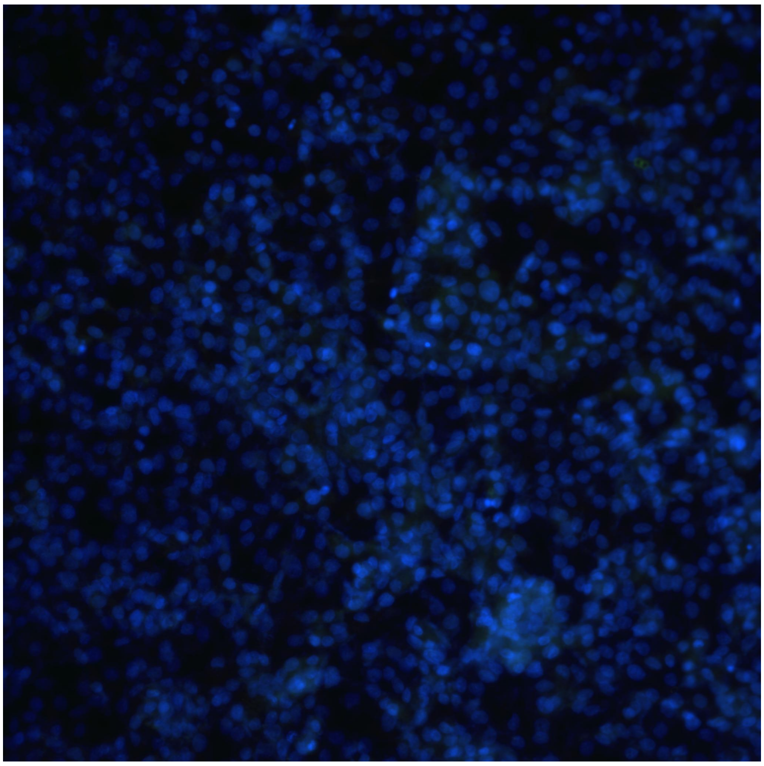

Supplement: Supplementary file 1 [file ppat.1014527.s004.zip › S1 File/S1 File/Figure S9A_immunofluorescences.pdf]
